# Development and validation of a risk prediction tool for the diagnosis of inflammatory bowel disease in patients presenting in primary care with abdominal symptoms

**DOI:** 10.1093/ecco-jcc/jjaf044

**Published:** 2025-03-18

**Authors:** Nosheen Umar, Steven Wambua, Phil Harvey, Samuel Cusworth, Krish Nirantharakumar, Shamil Haroon, Nigel Trudgill, Nicola J Adderley

**Affiliations:** Department of Gastroenterology, Sandwell and West Birmingham NHS Trust, West Bromwich, United Kingdom; Institute of Applied Health Research, University of Birmingham, Birmingham, United Kingdom; Institute of Applied Health Research, University of Birmingham, Birmingham, United Kingdom; Department of Gastroenterology, New Cross Hospital, Wolverhampton, United Kingdom; Institute of Applied Health Research, University of Birmingham, Birmingham, United Kingdom; Institute of Applied Health Research, University of Birmingham, Birmingham, United Kingdom; Institute of Applied Health Research, University of Birmingham, Birmingham, United Kingdom; Department of Gastroenterology, Sandwell and West Birmingham NHS Trust, West Bromwich, United Kingdom; Institute of Cancer and Genomic Sciences, University of Birmingham, Birmingham, United Kingdom; Institute of Applied Health Research, University of Birmingham, Birmingham, United Kingdom; National Institute for Health and Care Research (NIHR) Birmingham Biomedical Research Centre, Birmingham, United Kingdom

**Keywords:** inflammatory bowel disease, ulcerative colitis, Crohn’s disease, prediction model, diagnosis

## Abstract

**Introduction:**

Patients with inflammatory bowel disease (IBD) may experience delays in their diagnosis. This study aimed to develop and validate a risk prediction tool for IBD.

**Methods:**

A retrospective cohort study was conducted using primary care data from 2010 to 2019, including symptomatic patients aged ≥18. UK-based primary care databases linked to hospital records were utilized for model development and validation. Cox proportional hazards models were used to derive risk equations for IBD, ulcerative colitis (UC), and Crohn’s disease (CD) in men and women. Candidate predictors included demographics, comorbidities, symptoms, extraintestinal manifestations, and laboratory results. Model performance was evaluated using measures of fit, discrimination, and calibration at 1, 2, 3, and 5 years after symptom onset.

**Results:**

In total, 2 054 530 patients were included in the derivation cohort and 673 320 in the validation cohort. In the derivation cohort, 0.7% were diagnosed with IBD (66.3% UC and 33.7% CD). Predictors in the final IBD model included age, smoking, body mass index, gastrointestinal symptoms, extraintestinal manifestations, comorbidities, family history of IBD, and laboratory investigations. The model demonstrated good discrimination and calibration; C-statistic 0.78 (95% confidence interval [CI], 0.77–0.79) in men and 0.78 (95% CI, 0.77–0.79) in women. In the validation cohort, the model tended to slightly overestimate IBD risk at higher risk thresholds.

**Conclusions:**

A risk model using patient demographics, symptoms, and laboratory results accurately predicted IBD, UC, and CD at 1, 2, 3, and 5 years after symptom onset, potentially aiding in prioritizing patients for a referral or fecal calprotectin testing in primary care.

## Graphical Abstract

**Graphical Abstract:**
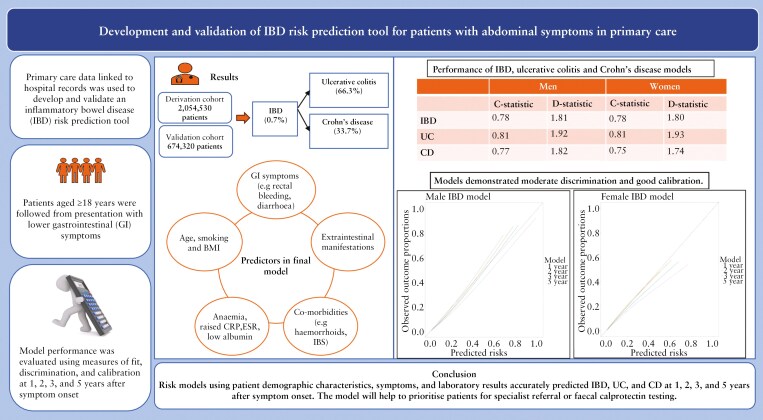

## 1. Introduction

Inflammatory bowel disease (IBD) includes ulcerative colitis (UC) and Crohn’s disease (CD). The prevalence of IBD in the United Kingdom is rising and is expected to reach 1% of the population by 2030.^1–3^ In primary care, patients with IBD may present initially with nonspecific gastrointestinal (GI) symptoms that are not always promptly recognized or appropriately followed up. Symptoms of IBD overlap with those of other GI disorders, such as irritable bowel syndrome (IBS) or infections, contributing to the diagnostic challenge.^4^ Median time from initial presentation in primary care to IBD diagnosis is 15.6 months.^5^ Longer times to IBD diagnosis may result in emergency admissions and surgery.^6–8^ More than 24 months to diagnosis was associated with twice the risk of surgery^6^ and 5 times the risk of emergency surgery in patients with CD.^9^

Studies suggest that IBD can be diagnosed earlier using symptoms and clinical investigations.^10–12^ However, most of these studies developed prediction models for CD alone.^13–15^ For example, a single-center study developed a risk prediction model based on blood, stool, and urine tests but this model is limited by small sample size, inclusion of both existing and new onset IBD patients, nongeneralizability as not population-based, and no internal or external validation.^16^

A recent systematic review of prediction models for the diagnosis and prognosis of IBD highlighted that no models currently predict a new diagnosis of IBD or differentiate UC from CD, in patients with suggestive symptomatology and biochemical abnormalities.^17^ A comprehensive approach to symptom evaluation and a high index of suspicion for IBD are important in primary care for timely and effective management. This highlights the need to develop a prediction model for IBD using routinely collected data in primary care. Such a model has the potential to reduce the duration of undiagnosed IBD symptoms and improve clinical outcomes. Furthermore, risk models are increasingly being integrated into clinical information systems, utilizing existing electronic health record data to enhance their implementation and effectiveness. A recent study highlighted sex-related differences in the clinical symptoms, extraintestinal manifestations, comorbidities, and disease severity in patients with IBD.^18,19^ Separate prediction models for men and women with IBD are therefore needed.

Our aim, therefore, was to develop and externally validate sex-specific risk prediction models for the diagnosis of IBD, UC, and CD for use in primary care.

## 2. Methods

### 2.1. Study design

A population-based retrospective open cohort study was undertaken between 1st January 2010 and December 31, 2019 including all patients aged 18 years or older with 1 or more symptoms associated with IBD, identified through SNOMED-CT codes, but no recorded diagnosis of IBD before entering the study. The first recorded presentation in primary care with GI symptoms during the study period was taken as the index date. The symptom codes used in the present study have been previously utilized in other IBD-related studies.^9^

### 2.2. Data source

The Clinical Practice Research Datalink (CPRD) Aurum primary care database linked to the Hospital Episode Statistics Admitted Patient Care (HES APC) database was used for the development and internal validation of the prediction models. The CPRD Gold primary care database linked to HES APC was used for external validation. These data were extracted using the Data Extraction for Epidemiological Research (DExtER) tool.^20^

CPRD Aurum is the largest UK primary care database, derived from routinely collected anonymized data from UK primary care providers using Egton Medical Information Systems Health electronic medical records (EMR) software, which is used by 56% of English primary care practices.^21^ It is representative of the England population in terms of demographics and the prevalence of key health conditions.^21^ Clinical data are largely recorded using SNOMED-CT codes, including information on symptoms, investigation findings, and diagnoses.^21^

In CPRD GOLD, approximately 6.9% of the UK population are included and patients are broadly representative of the UK general population in terms of age, sex, and ethnicity. It includes data from UK primary care practices that use Vision EMR software. It includes data from England, Scotland, Wales, and Northern Ireland.^22^

HES APC data provides information on elective and emergency care episodes in secondary care in England. Each recorded episode contains information on clinical diagnoses, procedures, and demographic, administrative, and geographical information. International Statistical Classification of Diseases and Related Health Problems, 10th Revision (ICD-10) codes are used for diagnoses and Office of Population Censuses and Surveys Classification of Interventions and Procedures version 4 (OPCS-4) codes for procedures.

### 2.3. Study participants

#### 2.3.1. Inclusion criteria

All practices contributing data to CPRD Aurum and CPRD GOLD were eligible for inclusion. Individual patients were eligible for inclusion from the latest of the following dates to ensure there was sufficient time for recording of baseline comorbidities: the date their practice began contributing to CPRD and a year after registration with their practice. Patients were included if they had GI symptoms recorded in primary care including diarrhea, rectal bleeding, change in bowel habit, weight loss, rectal mucus, bloating, abdominal pain, perianal pain, nausea, or vomiting.

#### 2.3.2. Exclusion criteria

The following patients were excluded from the study: those with less than 1 year of continuous data; below 18 years; a pre-existing IBD diagnosis before; and patients with no record of a consultation with GI symptoms. Practices with records in both datasets (CPRD Aurum and CPRD GOLD) were excluded from the external validation dataset (CPRD GOLD) based on their unique identifier to ensure no overlap between the development and validation cohorts.

### 2.4. Follow-up period

Patients were followed from the index date to the date of IBD diagnosis, death, transfer out of practice, last practice data collection, or study end (December 31, 2019), whichever occurred earliest. Patients were censored at 5 years because we aimed to predict IBD diagnosis within 5 years after the onset of symptoms, accounting for patients with milder disease.

### 2.5. Predictors for IBD prediction model

Candidate predictors were chosen based on clinical and published data.^23–25^ Patient-level candidate predictors included demographics (age, ethnicity, deprivation); clinical characteristics (body mass index [BMI], smoking status, Charlson comorbidity score); family history of IBD, diagnosis of IBS; existing mental health diagnoses (anxiety, depression); appendicectomy; GI symptoms (nausea and vomiting, abdominal pain, diarrhea, mucus in stool, anal symptoms, weight loss, bloating, rectal bleeding, and change in bowel habit) recorded at the index date; extraintestinal manifestations of IBD (mouth ulcers, dermatological [pyoderma gangrenosum, Sweet’s syndrome, psoriasis, erythema nodosum], ophthalmic [episcleritis, scleritis, uveitis], and [primary sclerosing cholangitis]) recorded any time prior to the index date; joint pain and swelling recorded in the 3 years prior to the index date; loperamide prescription recorded within a year prior to symptomatic presentation in primary care; laboratory investigations hemoglobin (Hb) (<12.9 g/dL in males and 11.9 g/dL in females), mean corpuscular volume (MCV) (<79 fl), platelet level (>400 × 10^9^/L), albumin level (<35 mg/dL), C-reactive protein (CRP) (>5 mg/dL), erythrocyte sedimentation rate (ESR) (>19 mm/h), ferritin (<20 mg/L), vitamin B12 level (<187 ng/L), and fecal calprotectin (FC) recorded 14 days before to 2 months after the index date. BMI recorded closest to the index date was categorized as <18 kg/m^2^ (underweight), 18–25 kg/m^2^ (normal weight), 25–30 kg/m^2^ (overweight), and >30 kg/m^2^ (obese). Smoking status was categorized as "smoker,” “ex-smoker,” and “nonsmoker.” A Charlson comorbidity score was calculated based on the comorbidities recorded at any time before the index date and categorized as “0” (no comorbidity), “1” (1 comorbidity), and “2” (2 and above comorbidities).^26,27^ Sex was not included as a predictor as sex-specific models were developed.

The same predictors were also considered when developing models for CD and UC. All candidate predictors were assessed for missing data, outliers/clinical plausibility of values and to ensure the correct measurement units were used.

### 2.6. Outcomes

The primary outcome was a recorded diagnosis of IBD (defined as either UC or CD). The secondary outcomes were a diagnosis of UC and CD separately. The SNOMED CT codes are in Table S1. A diagnosis of IBD was ascertained through a relevant clinical code in primary care or an IBD-related hospital admission in secondary care. The earliest date of a primary or secondary care record of IBD was assigned as the diagnosis date. Where patients had co-existing diagnoses of UC and CD, the latest secondary or primary care diagnostic code was used. Patients in whom the UC or CD diagnosis was unclear were included in the IBD analysis but excluded from the UC and CD analysis.

### 2.7. Sample size

A sample size calculation was performed using the pmsampsize package in R. Assuming a conservative overall model fit of Nagelkerke’s *R*^2^ of 0.15^28^ the incorporation of 50 predictor parameters required 40 659 participants (264 outcome events), or equivalently 5.27 events per predictor parameter, based on an event rate of 1.8/1000 person-years. The present study had substantially more participants and the events per predictor exceeded the prespecified number.

### 2.8. Missing data

For both development and external validation datasets, missing values for BMI were categorized as a separate missing category and included to facilitate the utilization of the model in patients with no BMI record. Missing values for smoking status were allocated to the nonsmoking category, as validated in a previous study.^29^ No entry of a health condition was taken to indicate the absence of the condition.

### 2.9. Statistical analysis

#### 2.9.1. Development and internal validation of risk prediction model

The risk prediction models were developed and validated using established methods.^30–32^ Baseline characteristics of categorical variables were presented as counts and percentages, while continuous variables were reported as mean and standard deviation (SD) or median and interquartile range (IQR).

Cox proportional hazards regression was used for the development of sex-specific risk prediction models for IBD and separately for UC and CD. The initial model for each outcome included all the candidate predictor variables and then the backward stepwise elimination method was used to select variables included in the final model. Backward elimination starts with a full model including all the potential candidate predictors and then the least significant predictors are eliminated one by one until only predictors with a significant *P*-value below the prespecified value (in this case *P* > .1) remain or no variable is left in the model. Fractional polynomials were used for age as it is a continuous variable.

Internal validation using 1000 bootstrap sampling repetitions was used to estimate the optimism-adjusted measures of predictive performance. The predictive performance of the developed models was assessed using model discrimination (how well it discriminates between patients with and without IBD) using the time-dependent C-statistic (range 0.5–1) and Royston’s D-statistic (range <1–>1), overall model fit using the *R*-squared statistic (a measure of explained variation, with higher values [closer to 1] indicating better performance), and calibration (agreement between predicted and observed risk) using calibration plots (plotting the predicted probability of the outcome against observed probability) at time points of interest (1, 2, 3, and 5 years after symptom onset) using the pseudo-value approach^33^ and by estimating the calibration slope. Regression coefficients of the final selected variables were then used to calculate the predicted probability of the outcome.

### 2.10. Sensitivity analysis

In a sensitivity analysis, whether FC improved model performance was assessed. FC was recorded 14 days before to 60 days after the index date. Categorized as “no record”, “<100 ug/g”, “100–199 ug/g”, “200–299 ug/g”, “500–1000 ug/g”, “>1000 ug/g,” and “missing” (a date for a calprotectin test recorded, but the value was missing). The model development process was repeated after the inclusion of FC as a candidate predictor. A complete case analysis was also performed.

Age interaction with diarrhea and rectal bleeding (the most common presenting symptoms) was examined. No difference in model performance was seen after including these interaction terms.

### 2.11. External validation of the prediction model

The 5-year risk equations derived from the development cohort for IBD, UC, and CD in men and women were applied to the validation cohort (CPRD GOLD) of patients aged >18 years with at least one of the relevant GI symptoms to assess model performance (C-statistic, D-statistic, *R*-squared, and calibration plots) at 5 years, and also at 1, 2, and 3 years after symptom onset.

Statistical analysis was performed using STATA, release 17, and R statistical software, R version 4.2.1.

This study has been reported following the recommended Transparent Reporting of a Multivariable Prediction Model for Individual Prognosis or Diagnosis (TRIPOD) guidelines (Supplementary Materials).

### 2.12. Personal and public involvement

Patients were involved in reviewing the lay summary and identifying potential candidate variables. The results were disseminated by presenting the findings to a GUT UK audience including patients and an expert-by-experience patient panel.

## 3. Results

### 3.1. Study population

**Figure 1.**
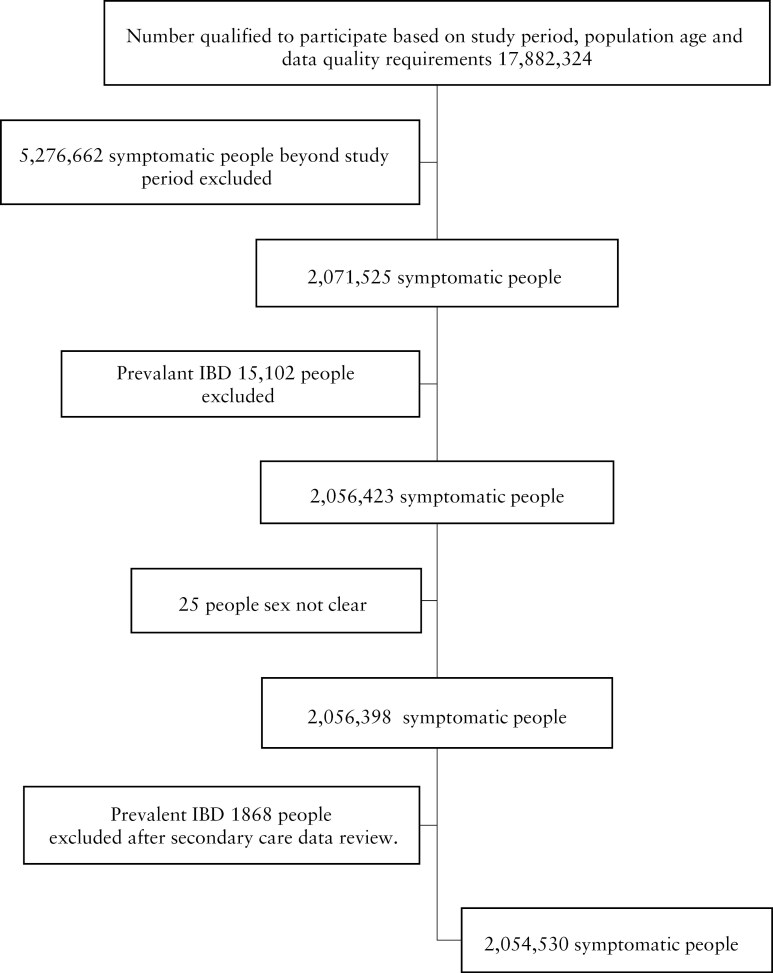
Study consort diagram for development cohort.

A total of 2 054 530 people from CPRD Aurum were included in the development cohort, and 673 320 people from CPRD GOLD were included in the external validation cohort (Figures 1 and S1).

### 3.2. Baseline characteristics

The baseline characteristics of people in the development and validation cohorts are described in Table 1. In the development cohort, 43.8% were male with a median age of 48.8 (IQR 34.8–64.7) years, and in the validation cohort, 44.2% were male with a median age of 49.9 (IQR 35.3–65.2) years. The population characteristics were similar in both the development and validation cohorts.

**Table 1.** Baseline characteristics of the study population with gastrointestinal symptoms.

	Development cohort				Validation cohort			
	Males	%	Females	%	Males	%	Females	%
Total	899 016	43.8	1 155 514	56.2	299 879	44.2	375 605	55.8
Age, median (IQR)	48.8 (34.8–64.7)		45.5 (31.0–63.8)		49.9 (35.3–65.2)		46.8 (31.4–64.4)	
Body mass index								
<18	18 421	2.0	40 332	3.5	5119	1.7	12 138	3.2
18.5–24.9	262 812	29.2	446 901	38.7	80 773	26.9	137 453	36.6
25–30	287 400	32.0	285 265	24.7	94 754	31.6	93 511	24.9
>30	177 620	19.8	254 202	22.0	62 414	20.8	87 599	23.3
Missing	152 763	17.0	128 814	11.1	56 819	18.9	44 904	12.0
Smoking status								
Never smoked	281 253	31.3	465 131	40.3	112 759	37.6	177 398	47.2
Ex-smoker	302 900	33.7	366 819	31.7	100 566	33.5	111 202	29.6
Current smoker	293 340	32.6	307 695	26.6	79 640	26.6	82 089	21.9
Missing	21 523	2.4	15 869	1.4	6914	2.3	4916	1.3
Symptoms								
Nausea and vomiting	68 099	7.6	128 340	11.1	28 539	9.5	50 746	13.5
Abdominal pain	357 222	39.7	576 787	49.9	121 008	40.4	189 019	50.3
Diarrhea	201 218	22.4	206 139	17.8	61 920	20.6	60 214	16.0
Mucus in stool	524	0.1	756	0.1	110	0.0	193	0.1
Anal symptoms	50 687	5.6	34 352	3.0	15 655	5.2	9939	2.6
Weight loss	52 018	5.8	51 202	4.4	14 973	5.0	13 617	3.6
Bloating	23 661	2.6	53 074	4.6	6533	2.2	14 970	4.0
Rectal bleeding	122 655	13.6	84 714	7.3	41 187	13.7	27 584	7.3
Change in bowel habit	30 307	3.4	29 754	2.6	11 456	3.8	10 895	2.9
Miscellaneous gastrointestinal symptoms	6394	0.7	6733	0.6	2747	0.9	2835	0.8
Extraintestinal manifestation								
Mouth ulcers	17 633	2.0	28 236	2.4	5658	1.9	8904	2.4
Primary sclerosing cholangitis	41	0.005	41	0.004	14	0.005	13	0.003
Joint swelling	43 279	4.8	64 021	5.5	1039	0.3	1606	0.4
Joint pain	232 578	25.9	303 765	26.3	10 444	3.5	16 741	4.5
Ophthalmic EIM	8872	1.0	11 381	1.0	3112	1.0	3864	1.0
Dermatological EIM	39 801	4.4	49 867	4.3	480	0.2	1363	0.4
Comorbidity score								
0	629 654	70.0	854 728	74.0	213 681	71.3	284 519	75.7
1	60 165	6.7	60 513	5.2	30 037	10.0	26 534	7.1
2	68 524	7.6	105 501	9.1	22 793	7.6	32 723	8.7
>2	140 673	15.6	134 772	11.7	33 368	11.1	31 829	8.5
Coexisting conditions								
Anxiety	103 763	11.5	193 415	16.7	30 821	10.3	55 651	14.8
Depression	131 439	14.6	257 662	22.3	49 987	16.7	95 917	25.5
Irritable bowel syndrome	27 675	3.1	75 885	6.6	8709	2.9	24 702	6.6
Hemorrhoids	81 286	9.0	93 183	8.1	23 024	7.7	25 573	6.8
Appendicectomy	40 390	4.5	52 875	4.6	14 036	4.7	17 436	4.6
Family history of IBD	470	0.1	655	0.1	130	0.04	183	0.05
Drugs								
Loperamide	27 106	3.0	29 550	2.6	11 853	4.0	12 298	3.3
Stool and blood tests								
Fecal calprotectin	10 095	1.1	10 981	1.0	1966	0.7	2223	0.6
Low hemoglobin	49 244	5.5	71 924	6.2	15 306	5.1	21 297	5.7
MCV (<79 fl)	8643	1.0	18 511	1.6	2083	0.7	4351	1.2
Ferritin (<20 mg/L)	4366	0.5	26 439	2.3	1393	0.5	7193	1.9
Vitamin B12 (<187 ng/L)	4525	0.5	6130	0.5	1785	0.6	2612	0.7
Albumin (<35 mg/dL)	24 835	2.8	33 257	2.9	8348	2.8	11 088	3.0
Platelets (>400 × 109/L)	12 276	1.4	26 015	2.3	3867	1.3	8195	2.2
CRP(>5 mg/dL)	46 808	5.2	59 723	5.2	16 940	5.6	21 436	5.7
ESR(>19 mm/h)	19 123	2.1	37 019	3.2	5522	1.8	10 363	2.8

Abbreviations: CRP, C-reactive protein; ESR, erythrocyte sedimentation rate; EIM, extraintestinal manifestations; Hb: hemoglobin; IBD: inflammatory bowel disease; MCV: mean corpuscular volume.

Dermatological EIM (psoriasis, Sweet’s syndrome, pyoderma gangreosum, erythema nodosum); ophthalmic EIM (iritis, episcleritis, scleritis, and uveitis).

The commonest presenting symptoms were abdominal pain in 39.7% of men and 49.9% of women, diarrhoa in 22.4% and 17.8%, and rectal bleeding in 13.6% and 7.3%, respectively. FC was recorded in only 1% of people.

The demographic details of people with and without IBD in both sexes are shown in Supplementary Tables 2a and 2b.

### 3.3. Incident IBD, ulcerative colitis, and Crohn’s disease

Of 15 105 patients with an incident IBD diagnosis in the development cohort, 10 014 (66.3%) had UC, 5088 (33.7%) CD, and 3 (0.1%) undifferentiated IBD. The median follow-up in the development cohort was 3.6 years (IQR 1.5–6.3) and 741 228 (36%) patients had more than 5 years follow-up. In the validation cohort median, follow-up was 3.4 years (IQR 1.4–6.1) and 232 069 (34%) patients had more than 5 years of follow-up.

### 3.4. Model development

#### 3.4.1. Predictor variables

Tables 2 and 3 show the predictor variables included in the models along with adjusted hazard ratios for women and men for incident IBD, UC, and CD in the development cohort. Important predictors in the final model included smoking, symptoms (diarrhea, rectal bleeding, change in bowel habit, and mucus per rectum), extraintestinal manifestations (dermatological, ophthalmic, and primary sclerosing cholangitis), coexisting conditions (IBS and hemorrhoids), family history of IBD, appendicectomy, loperamide prescription, anemia (low Hb, MCV, and ferritin), low albumin, and raised inflammatory markers (CRP, ESR, and platelets). In addition to these predictors, the final IBD model for women included anxiety, low vitamin B12 levels, and joint swelling, while the final model for men included depression and joint pain.

**Table 2.** Adjusted hazard ratios (95% CI) for incident IBD, ulcerative colitis and Crohn’s disease in women in the development cohort.

Predictors	IBD model	Ulcerative colitis model	Crohn’s disease model
Smoking status (reference nonsmoker)			
Ex-smoker	1.37 (1.29−1.46)	1.40(1.30−**1.51**)	1.29 (1.16−1.43)
Current smoker	1.31 (1.23−1.39)	1.11(**1.03**−**1.21**)	1.66 (1.50−1.83)
Body mass index (reference normal BMI)			
Underweight	0.98 (0.85−1.12)	1.03 (0.86−1.23)	0.88 (0.70−1.11)
Overweight	0.90 (0.84−0.95)	0.87 (0.80−0.94)	0.95 (0.86−1.06)
Obese	0.74 (0.69−0.79)	0.74 (0.68−0.80)	0.75 (0.67−0.84)
Missing	0.89 (0.82−0.97)	0.89 (0.80−0.99)	0.89 (0.77−1.02)
Comorbidity score (reference 0 score)			
1	1.00 (0.89−1.12)	0.92 (0.79−1.07)	1.15 (0.96−1.39)
2	0.89 (0.81−0.98)	0.91 (0.81−1.02)	0.88 (0.74−1.04)
>2	0.81 (0.73−0.89)	0.86 (0.75−0.97)	0.74 (0.61−0.88)
Symptoms			
Change in bowel habit	3.56 (3.09−4.12)	3.71 (3.01−4.58)	3.77 (3.02−4.70)
Abdominal pain	0.75 (0.66−0.84)	0.58 (0.48−0.70)	1.22 (1.05−1.43)
Nausea and vomiting	0.57 (0.49−0.67)	0.52 (0.41−0.65)	0.79 (0.64−0.98)
Diarrhea	2.10 (1.87−2.37)	2.10 (1.74−2.54)	2.46 (2.10−2.89)
Mucus per rectum	9.02 (6.46−12.60)	9.57 (6.37−14.37)	8.95 (4.92−16.27)
Weight loss	0.58 (0.47−0.71)	0.44 (0.32−0.60)	^*^
Bloating	0.73 (0.61−0.88)	0.70 (0.54−0.91)	^*^
Rectal bleeding	5.15 (4.57−5.80)	6.82 (5.66−8.23)	2.45 (2.03−2.94)
Anal symptoms	^*^	0.72 (0.53−0.97)	1.88 (1.43−2.46)
Extraintestinal manifestation (EIM)			
Mouth ulcer	^*^	^*^	1.28 (1.03−1.60)
Ophthalmic EIM	1.60 (1.31−1.95)	1.45 (1.12−1.88)	1.74 (1.27−2.38)
Primary sclerosing cholangitis	12.21 (3.93−37.94)	20.91 (6.72−65.06)	^*^
Dermatological EIM	1.28 (1.15−1.42)	1.14 (0.99−1.31)	1.48 (1.26−1.75)
Joint swelling	0.87 (0.77−0.98)	0.87 (0.75−1.02)	^*^
Joint pain	^*^	0.89 (0.82−0.96)	1.09 (0.99−1.20)
Coexisting conditions			
Anxiety	0.86 (0.81−0.93)	0.83 (0.76−0.91)	^*^
Irritable bowel syndrome	1.29 (1.18−1.41)	1.11 (0.99−1.24)	1.62 (1.42−1.85)
Hemorrhoids	1.26 (1.16−1.36)	1.24 (1.13−1.36)	1.27 (1.10−1.46)
Family history of IBD	5.01 (3.40−7.36)	4.19 (2.48−7.09)	4.95 (2.80−8.76)
Loperamide	1.45 (1.29−1.64)	1.34 (1.14−1.57)	1.66 (1.37−2.00)
Appendicectomy	0.71 (0.62−0.82)	0.55 (0.45−0.66)	^*^
Blood tests			
Low hemoglobin	1.45 (1.33−1.59)	1.45 (1.30−1.62)	1.46 (1.27−1.69)
Low MCV	1.26 (1.10−1.44)	^*^	1.64 (1.35−2.00)
Low ferritin	1.47 (1.31−1.65)	1.62 (1.41−1.86)	1.28 (1.05−1.55)
Raised platelets	2.77 (2.52−3.04)	2.21 (1.94−2.52)	3.51 (3.05−4.03)
Raised CRP	2.64 (2.44−2.86)	2.37 (2.13−2.63)	3.04 (2.69−3.44)
Raised ESR	1.89 (1.73−2.08)	1.83 (1.62−2.07)	1.94 (1.68−2.24)
Low albumin	2.18 (1.97−2.41)	1.80 (1.57−2.07)	2.68 (2.31−3.11)
Low vitamin B12	1.37 (1.08−1.72)	^*^	2.05 (1.51−2.78)

Abbreviations: CRP, C−reactive protein; ESR, erythrocyte sedimentation rate; EIM, extraintestinal manifestations; Hb, hemoglobin; IBD, inflammatory bowel disease; MCV, mean corpuscular volume.

^*^Not included in final model as not associated.

**Table 3.** Adjusted hazard ratios (95% CI) for incident IBD, ulcerative colitis and Crohn’s disease in men in the development cohort.

Predictors	IBD model	Ulcerative colitis model	Crohn’s disease model
Smoking status (reference nonsmoker)			
Ex-smoker	1.39 (1.31−1.47)	1.47 (1.38−1.58)	1.18 (1.06−1.32)
Current smoker	1.09 (1.03−1.16)	1.01 (0.94−1.09)	1.27 (1.16−1.41)
Body mass index (reference normal BMI)			
Underweight	1.03 (0.89−1.20)	0.95 (0.77−1.16)	1.14 (0.90−1.45)
Overweight	0.95 (0.89−1.01)	0.92 (0.86−0.99)	0.99 (0.89−1.11)
Obese	0.83 (0.78−0.90)	0.82 (0.75−0.89)	0.86 (0.75−0.98)
Missing	0.99 (0.92−1.06)	0.96 (0.89−1.05)	1.03 (0.92−1.16)
Comorbidity score (reference 0 score)			
1	1.08 (0.97−1.19)	1.13 (1.00−1.27)	0.98 (0.81−1.18)
2	0.92 (0.83−1.02)	0.95 (0.84−1.07)	0.85 (0.69−1.04)
>2	0.77(0.70−0.84)	0.82 (0.74−0.92)	0.68 (0.57−0.81)
Symptoms			
Change in bowel habit	3.54 (3.13−4.01)	4.34 (3.68−5.11)	2.60 (2.17−3.11)
Abdominal pain	0.80 (0.72−0.88)	0.70 (0.61−0.82)	^*^
Nausea and vomiting	0.40 (0.34−0.48)	0.39 (0.31−0.51)	0.45 (0.35−0.57)
Diarrhea	2.00 (1.81−2.21)	2.51 (2.17−2.91)	1.41 (1.27−1.57)
Mucus per rectum	4.71 (2.96−7.51)	6.50 (3.90−10.82)	^*^
Weight loss	0.45 (0.37−0.54)	0.39 (0.30−0.52)	0.57 (0.45−0.72)
Bloating	0.73 (0.59−0.92)	^*^	0.61 (0.42−0.89)
Rectal bleeding	3.70 (3.34−4.09)	5.51 (4.77−6.36)	1.43 (1.26−1.61)
Anal symptoms	^*^	0.75 (0.60−0.94)	1.47 (1.24−1.75)
Extraintestinal manifestation (EIM)			
Mouth ulcer	1.28 (1.11−1.48)	1.39 (1.17−1.64)	^*^
Ophthalmic EIM	1.30 (1.06−1.61)	^*^	1.44 (1.00−2.07)
Primary sclerosing cholangitis	7.21 (3.20−16.21)	6.82 (2.53−18.40)	11.33 (2.79−45.93)
Dermatological EIM	1.13 (1.02−1.26)	^*^	1.30 (1.09−1.56)
Joint pain	0.82 (0.77−0.87)	0.81 (0.75−0.87)	0.85 (0.74−0.98)
Coexisting conditions			
Depression	0.89 (0.83−0.95)	0.83 (0.77−0.91)	^*^
Irritable bowel syndrome	1.30 (1.16−1.47)	1.14 (0.98−1.33)	1.64 (1.36−2.00)
Hemorrhoids	1.19 (1.10−1.28)	1.13 (1.03−1.23)	1.33 (1.16−1.53)
Family history of IBD	3.10 (1.93−5.00)	2.60 (1.40−4.85)	4.42 (2.10−9.28)
Loperamide	1.23 (1.09−1.38)	1.21 (1.05−1.40)	1.28 (1.03−1.59)
Appendicectomy	0.54 (0.47−0.63)	0.38 (0.31−0.48)	
Blood tests			
Low hemoglobin	1.63 (1.48−1.81)	1.41 (1.25−1.60)	2.15 (1.81−2.56)
Low MCV	1.38 (1.19−1.60)	^*^	1.87 (1.50−2.32)
Low ferritin	1.96 (1.64−2.34)	2.40 (1.96−2.94)	1.40 (1.01−1.96)
Raised platelets	2.61 (2.35−2.91)	2.16 (1.87−2.48)	3.18 (2.68−3.78)
Raised CRP	3.82 (3.56−4.10)	3.74 (3.43−4.08)	3.84 (3.40−4.35)
Raised ESR	1.83 (1.65−2.02)	1.89 (1.67−2.15)	1.60 (1.34−1.90)
Low albumin	2.01 (1.81−2.24)	1.85 (1.62−2.12)	2.22 (1.85−2.65)

Abbreviations: CRP, C−reactive protein; ESR, erythrocyte sedimentation rate; EIM, extraintestinal manifestations; Hb, hemoglobin; IBD, inflammatory bowel disease; MCV, mean corpuscular volume .

^*^Not included in final model as not associated.

Complete case analysis after excluding patients with missing BMI data showed similar predictors in the IBD models for men and women (Table S3).

### 3.5. Risk equations for males and females

The 5-year risk equations for men and women for IBD risk are described later. The coefficients of the final IBD model in both sexes are shown in Table S4.

**Table:** 

Patient 5-year IBD risk equation in women
1 – 0.99635^exp (1.579658 ×$\left({{\left(\;\frac{age}{10}\right)}^{-2}}^{}-0.0428173966\right)$) – (0.0017741 ×$\left({{\left(\frac{age}{10}\right)}^{3}}^{}\;112.867684\right)$) + (0.3168358 × ex-smoker) + (0.2691738 × current smoker) – (0.0247878 × BMI < 18 kg/m^2^) – (0.1104974 × BMI 25–30 kg/m^2^) – (0.3046951 × BMI > 30 kg/m^2^) – (0.1182983 × Missing BMI) – (0.000814 × comorbidity score of 1) – (0.1114679 × comorbidity score of 2) – (0.2158126 × comorbidity score of >2) + (1.270727 × Change in bowel habit) – (0.2938484 × abdominal pain) – (0.5614838 × nausea and vomiting) + (0.7429809 × Diarrhea) + (2.19955 × mucus in stool) – (0.5479528 × weight loss) – (0.3153851 × bloating) + (1.638549 × rectal bleeding) + (0.4685556 × ophthalmic extraintestinal manifestation) + (2.501881 × primary sclerosing cholangitis) – (0.1409276 × joint swelling) + (0.2441694 × dermatological extraintestinal manifestation) – (0.1466258 × anxiety) + (0.2538555 × irritable bowel syndrome) + (1.610818 × family history of IBD) + (0.2287947 × hemorrhoids) + (0.3746028 × Loperamide) – (0.3443312 × appendicectomy) + (0.3745337 × anemia) + (1.018102 × raised platelets) + (0.2306289 × low MCV) + (0.7777982 × low albumin) + (0.3114264 × low vitamin B12) + (0.3865922 × low ferritin) + (0.971512 × raised CRP) + (0.6380655 × raised ESR)

**Table:** 

Patient 5-year IBD risk equation in men
1 – 0.995305^exp ((2.597693 ×$\left({\left(\frac{age}{10}\right)}^{-2}-0.0396522836\right)$) – (0.0022372 × ($\left({{\left(\frac{age}{10}\right)}^{3}}^{}126.6478128\right)$)) + (0.3293201 × ex-smoker) + (0.0865584 × current smoker) + (0.0325487 × BMI <18) – (0.0526996 × BMI 25–30) – (0.1805035 × BMI >30) – (0.0128515 × BMI missing) + (0.0746145 × comorbidity score of 1) – (0.0870168 × comorbidity score of 2) – (0.2661331 × comorbidity score of >2) + (1.265126 × change in bowel habit) – (0.2264712 × abdominal pain) – (0.9056818 × nausea and vomiting) + (0.6930883 × diarrhea) + (1.550392 × mucus in stool) – (0.8053466 × weight loss) – (0.3082227 × bloating) + (1.307207 × rectal bleeding) + (0.2460937 × mouth ulcers) + (0.2646168 × ophthalmic extraintestinal manifestation) + (1.97502 × primary sclerosing cholangitis) – (0.1988999 × joint pain) + (0.1225519 × dermatological extraintestinal manifestation) – (0.1211607 × depression) + (0.2635726 × irritable bowel syndrome) + (1.13236 × family history of IBD) + (0.1718364 × hemorrhoids) + (0.2042413 × Loperamide) – (0.6102908 × appendicectomy) + (0.4900032 × anemia) + (0.9604751 × raised platelets) + (0.3239654 × low MCV) + (0.6994172 × low albumin) + (0.672573 × low ferritin) + (1.340137 × raised CRP) + (0.6025531 × raised ESR)

### 3.6. Internal model validation

Table 4 shows the performance measures for IBD, UC, and CD in both sexes in the development cohort. The IBD model in men predicting 5-year risk of IBD performed well with a C-statistic of 0.76 (95% confidence interval [CI], 0.76–0.77) and D-statistic of 1.70 (95% CI, 1.66–1.74). Corresponding values in women were similarly a C-statistic of 0.77 (95% CI, 0.76–0.77) and D-statistic of 1.70 (95% CI, 1.66–1.74).

**Table 4.** Performance measures for IBD, ulcerative colitis and Crohn’s disease in the development and validation cohorts in women and men (mean [95% confidence intervals] at different time points.

	Men			Women		
	C-statistic^*^	D-statistic^*^	*R*-squared D	C-statistic^*^	D-statistic^*^	*R*-squared D
Development cohort						
Inflammatory bowel disease						
1 year	0.80 (0.79−0.80)	2.02 (1.98−2.07)	0.49	0.81 (0.80−0.82)	2.14 (2.09−2.19)	0.52
2 years	0.78 (0.77−0.79)	1.87 (1.83−1.91)	0.45	0.79 (0.78−0.80)	1.94 (1.90−1.99)	0.47
3 years	0.77 (0.77−0.78)	1.79 (1.75−1.83)	0.43	0.78 (0.77−0.78)	1.83 (1.79−1.87)	0.44
**5 years	0.76 (0.76−0.77)	1.70 (1.66−1.74)	0.41	0.77(0.76−0.77)	1.70 (1.66−1.74)	0.41
Ulcerative colitis						
1 year	0.83 (0.82−0.83)	2.16 (2.10−2.21)	0.53	0.84 (0.83−0.85)	2.29 (2.23−2.36)	0.56
2 years	0.81 (0.80−0.82)	2.01 (1.96−2.06)	0.49	0.82 (0.81−0.83)	2.11 (2.05−2.17)	0.52
3 years	0.80 (0.80−0.81)	1.93 (1.88−1.98)	0.47	0.80 (0.80−0.81)	1.98 (1.92−2.03)	0.48
**5 years	0.79 (0.79−0.80)	1.83 (1.78−1.87)	0.44	0.79 (0.78−0.80)	1.82 (1.77−1.87)	0.44
Crohn’s disease						
1 year	0.76 (0.75−0.78)	1.96 (1.87−2.04)	0.48	0.78 (0.77−0.80)	2.06 (1.98−2.15)	0.50
2 years	0.75 (0.73−0.76)	1.79 (1.71−1.86)	0.43	0.76 (0.75−0.78)	1.87 (1.79−1.94)	0.45
3 years	0.74 (0.73−0.75)	1.73 (1.66−1.80)	0.42	0.76 (0.75−0.77)	1.78 (1.71−1.85)	0.43
**5 years	0.73(0.72−0.75)	1.64(1.57−1.71)	0.39	0.75(0.74−0.76)	1.70(1.63−1.76)	0.41
Validation cohort						
Inflammatory bowel disease						
1 year	0.81 (0.80−0.82)	2.13 (2.05−2.22)	0.52	0.82 (0.81−0.84)	2.26 (2.16−2.37)	0.55
2 years	0.80 (0.78−0.81)	2.00 (1.92−2.08)	0.49	0.80 (0.79−0.82)	2.08 (1.99−2.17)	0.51
3 years	0.79 (0.78−0.80)	1.92 (1.84−1.99)	0.47	0.79 (0.78−0.80)	1.95 (1.87−2.03)	0.48
**5 years	0.78 (0.77−0.79)	1.81(1.73−1.88)	0.44	0.78 (0.77−0.79)	1.80 (1.72−1.88)	0.44
Ulcerative colitis						
1 year	0.84 (0.83−0.85)	2.25 (2.14−2.35)	0.55	0.86 (0.84−0.88)	2.43 (2.30−2.56)	0.58
2 years	0.82 (0.81−0.84)	2.11 (2.01−2.20)	0.51	0.84 (0.82−0.85)	2.23 (2.12−2.35)	0.54
3 years	0.82 (0.80−0.83)	2.03 (1.94−2.13)	0.50	0.82 (0.80−0.84)	2.10 (2.00−2.21)	0.51
**5 years	0.81 (0.80−0.82)	1.92 (1.83−2.01)	0.47	0.81 (0.79−0.82)	1.93 (1.83−2.03)	0.47
Crohn’s disease						
1 year	0.80 (0.77−0.82)	2.25 (2.10−2.40)	0.55	0.79 (0.76−0.81)	2.23 (2.07−2.39)	0.54
2 years	0.79 (0.77−0.81)	2.14 (2.00−2.27)	0.52	0.78 (0.75−0.80)	2.05 (1.91−2.19)	0.50
3 years	0.78 (0.76−0.80)	2.01 (1.88−2.14)	0.49	0.76 (0.74−0.79)	1.92 (1.79−2.05)	0.47
**5 years	0.77 (0.75−0.79)	1.82 (1.71−1.94)	0.44	0.75 (0.74−0.77)	1.74 (1.63−1.86)	0.42

^*^Measures of discrimination: higher values indicate better discrimination.

^**^Final model.

### 3.7. External model validation

Table 4 shows the performance measures of the IBD, UC, and CD models in both sexes in the validation cohort at 1,2, 3, and 5 years after symptom onset, respectively. The IBD model in women predicting 5-year risk of IBD showed that the fitted predictors in the model explained 44% of the variation in IBD diagnosis (*R*-squared). The model again performed well with a D-statistic of 1.80 (95% CI, 1.72–1.88) and a C-statistic of 0.78 (95% CI, 0.77–0.79). Corresponding values in the IBD model for men at 5 years were *R*-squared D of 44%, D-statistic 1.81 (95% CI, 1.73–1.88), and C-statistic 0.77 (95% CI, 0.76–0.79). All models showed similar performance measures in both sexes in the validation cohort and development cohort.

### 3.8. Calibration

**Figure 2.**
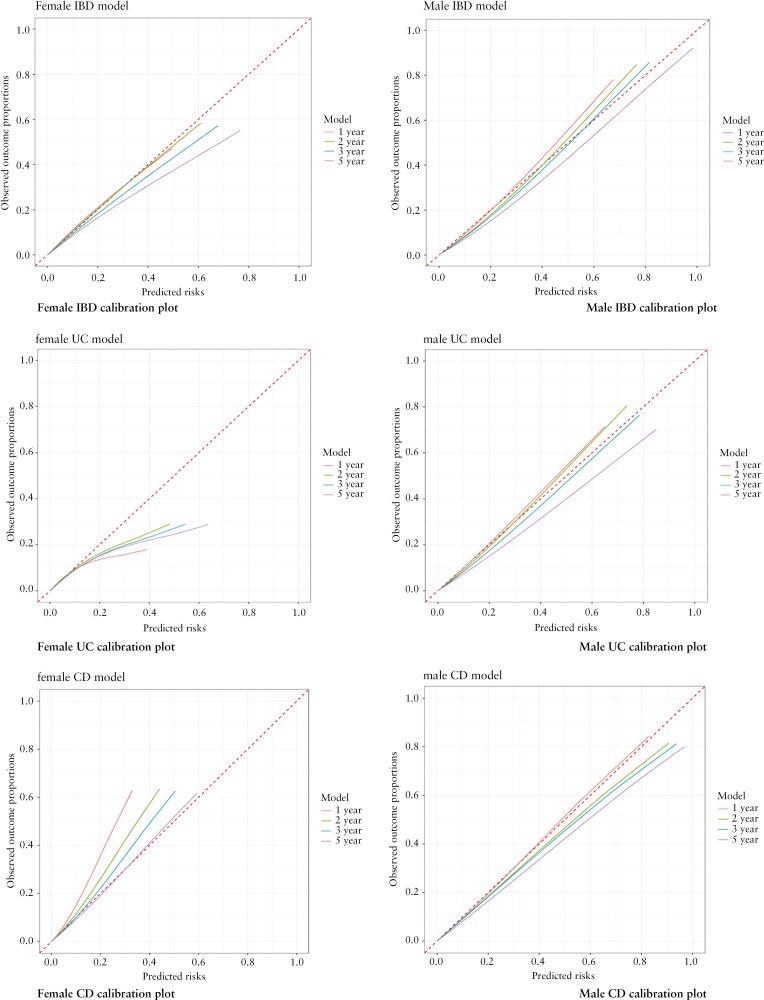
Calibration plots for men and women predicting inflammatory bowel disease (IBD), ulcerative colitis (UC), and Crohn’s disease (CD) at 1, 2, 3, and 5 years in the validation cohort.

**Figure 3.**
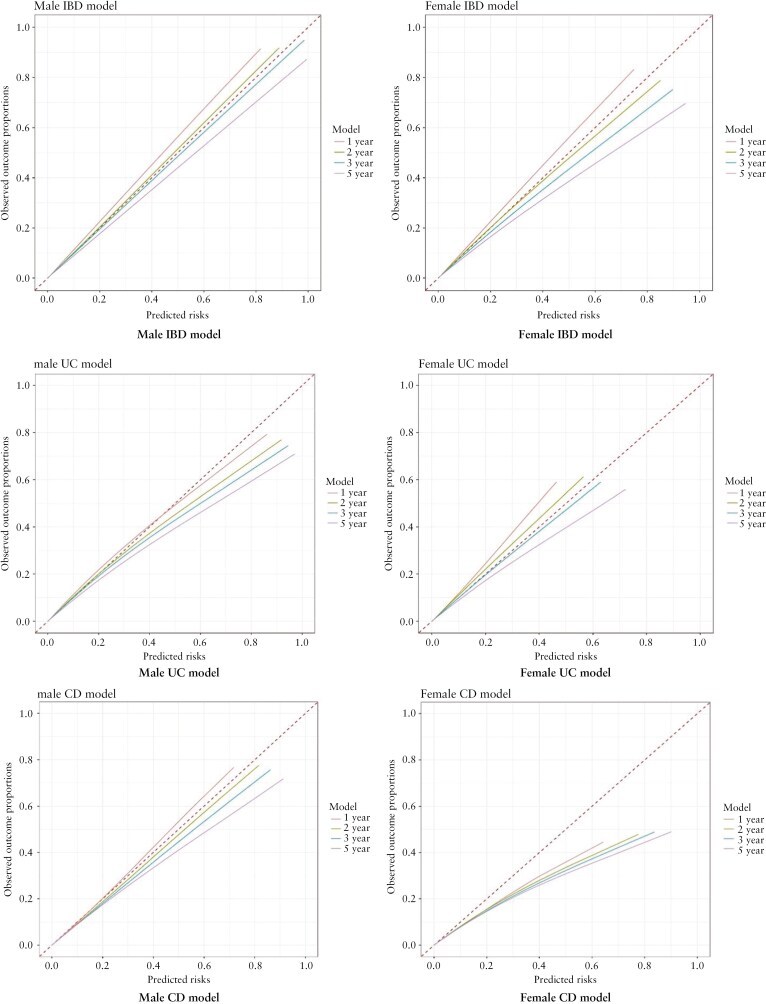
Calibration plots for inflammatory bowel disease (IBD), ulcerative colitis (UC), and Crohn’s disease (CD) in the development cohort at 1, 2, 3, and 5 years.

Figures 2 and 3 show the calibration plots in the validation and development cohorts for males and females for IBD at 1, 2, 3, and 5 years after symptom onset. The IBD model for males was well calibrated at lower risk thresholds at 1, 2, and 3 years but underestimated risks at higher thresholds, while it overestimated risks at higher thresholds at 5 years. The female IBD model was well calibrated at 1 and 2 years, while at 3 and 5 years, the model overestimated the risk at higher thresholds.

### 3.9. Sensitivity analysis

The hazard ratios of the models with FC)are shown in Tables S5 and S6. Higher FC levels were associated as expected with a much higher risk of a later diagnosis of IBD in both sexes (FC level of >1000 compared to no record were adjusted hazard ratios (aHR) 25.98 (95% CI, 20.96–32.20) in women and 19.85 (95% CI, 16.01–24.62) in men respectively).

The performance measures of the IBD, UC, and CD models including FC are shown in Table S7. The performance measures of models that included FC did not differ significantly from those of models that excluded FC. For example, for the male IBD model in the development cohort, the addition of FC yielded a C-statistic of 0.77 (95% CI, 0.76–0.78) and a D-statistic of 1.79 (95% CI, 1.75–1.83). Figure S2 shows the calibration plots for the IBD, UC, and CD models with FC at 1, 2, 3, and 5 years in the validation cohort.

Table S8 presents the aHR for IBD in men and women within the development cohort, as determined through complete case analysis including FC.

## 4. Discussion

### 4.1. Main findings

We have developed and validated risk prediction models for the diagnosis of IBD, and specifically UC and CD, in men and women presenting to primary care with GI symptoms. These models incorporated demographic characteristics, clinical symptoms, extraintestinal manifestations, and blood test results recorded in primary care.

Good performance measures of the IBD model were observed in men and women in the external validation. In men, the model showed good discrimination and was well calibrated for predicting the IBD risk at 3 years. In women, the model also showed good discrimination and was well calibrated at 1 and 2 years. Both the UC and CD models showed good discrimination in both sexes. The UC model was well calibrated at 3 years in men but over-predicted at higher thresholds in women. The CD model was well calibrated at 1 year in men and at 5 years in women. No significant difference in model performance was observed after the inclusion of FC.

Patients with IBD exhibit varying disease severity, influencing the time to clinical diagnosis. Severe cases are typically diagnosed rapidly within 1–2 years, while milder cases may take several years. Predictive models aim to expedite diagnosis using readily available data. These models incorporate varying time horizons to reflect this spectrum of disease severity. One-year models are better at identifying severe cases, whereas 2-, 3- and 5-year models are more effective at predicting risk in milder cases. By examining different time points, these models performed well at 1, 2, 3, and 5 years after symptom onset and therefore across the full range of disease severity seen in primary care settings and can facilitate earlier diagnosis.

### 4.2. Comparison with the existing literature

This study showed that 66.3% of patients had UC and 34.7% had CD, and a similar ratio has been observed in other epidemiological studies estimating the incidence and prevalence of UC and CD in the United Kingdom.^2^

As highlighted in a recent systematic review of existing prediction models for the diagnosis and prognosis of IBD, there is a scarcity of prediction models for the diagnosis of IBD in symptomatic patients presenting to primary care.^17^ Most of the diagnostic prediction models in IBD are focused on either the diagnosis of extraintestinal manifestations or predicting severity of inflammation in patients with CD and UC.^10,11,34^

To identify patients at higher risk of IBD, several scoring systems have been proposed. A machine learning prediction model to distinguish IBD patients from non-IBD patients based on baseline blood, urine, and stool test records was proposed in Poland. This study was conducted in a secondary care setting using a case–control study design.^16^ It has several limitations, which include using data of both prevalent and newly diagnosed IBD patients, a small sample size, and a lack of external validation of the model.

An Italian study proposed a “Red Flags Index for suspected CD,” which was based on signs and symptoms identified through a systematic literature review. The model was subject to recall bias as CD patients were asked to recall their symptoms before diagnosis. Second, the sample size of the study was small and not representative of the incidence and prevalence of IBD in the general population.^35^ The external validation of the Red flag tool demonstrated poor performance with 50% sensitivity and 58% specificity.^36^

The IBD-REFER criteria based on signs, symptoms, and blood tests, selected through a Delphi consensus exercise in Israel were proposed to predict the diagnosis of UC and CD in adults and children.^37^ Patients enrolled in this study were patients with a gastroenterology referral so the population was not representative of a primary care patient cohort.^38^ IBD-REFER criteria used FC as an additional rather than mandatory predictor due to its limited use by primary care physicians and low specificity.^37^

CalproQuest (an 8-item questionnaire) has been proposed in Swiss primary care for predicting IBD diagnosis in symptomatic patients. Patients with a higher score were offered an FC test. It has not been externally validated.^38^

A meta-analysis of IBD prediction models based on lower GI symptoms in pediatric patients highlighted that the addition of blood tests (CRP, ESR, Hb, platelets, and albumin) to a symptom-based model improves the model performance in a hospital setting.^39^ It also highlighted that most of the studies evaluating the performance of the addition of blood or stool test to symptom-based models were based on secondary care data (referred patients), and none were based in primary care.

The diagnostic accuracy of FC for diagnosing IBD in secondary care is reported as an area under the curve (AUC) of 0.97.^40^ In a primary-care-based study, a diagnostic accuracy AUC of 0.89 (95% CI, 0.85–0.93) has been reported for patients aged 18–45 years presenting with lower GI symptoms who had an FC test result available.^41^ In the present study, models that included FC did not show any improvement in performance, as FC was not available for most patients.

### 4.3. Clinical utility of the IBD prediction model

The diagnostic accuracy of FC testing in patients with lower GI symptoms is lower in primary care compared to secondary care due to the lower prevalence of IBD in the general population.^40–43^ To increase the pretest probability of FC, noninvasive tools are required.^38^ A recent review article highlights the uncertainties regarding which patients should be targeted for FC testing.^44^

The NICE guidelines recommend that all patients with lower GI symptoms are eligible for FC testing, provided that cancer is not suspected.^45^ In contrast, the BSG guidelines recommend targeting only patients with chronic diarrhea.^46^ An IBD prediction model based on routinely collected data and common blood tests, as presented here, may identify patients who need FC testing in primary care and subsequent referral to secondary care for endoscopy. It will also guide clinicians on the probability of IBD diagnosis in patients with nonspecific lower GI symptoms.

The models were developed using data that was recorded in the electronic health records through routine care and should be readily implementable in UK-based and similar primary care clinical information systems. The complexity of the models is unlikely to affect the take-up of models as they are designed to be calculated automatically from the electronic patient record and to provide the clinician with a predicted probability of IBD, which they can use to support their decision-making. This model may also be used in patients who fail to return their FC test sample, as highlighted in a study indicating that only one-third of patients returned their sample when requested in primary care.^47^ Furthermore, it could be used in patients with inconclusive FC results in primary care to support the clinician’s decision on whether to refer patients to secondary care. These risk models can guide the use of FC testing by identifying individuals at increased risk of IBD. This approach will enable primary care physicians to prioritize FC testing for patients at increased risk of developing IBD. These risk models can also assist gastroenterologists in triaging patients at risk of IBD, identifying those requiring prompt further evaluation, including colonoscopy and other investigations.

We acknowledge that these models are particularly relevant to the UK healthcare system and similar systems with strong primary care frameworks, where patients typically consult a family physician before specialist referral. However, their applicability may be limited in healthcare systems with more direct access to secondary and tertiary care.

Randomized impact trials are needed to evaluate the effect of implementing these models on the diagnosis of IBD and on their cost-effectiveness. Further research is also needed to determine the optimum risk thresholds of the models and to evaluate their performance in different ethnic groups.

### 4.4. Strengths and limitations

The key strength of this study is the large sample size which is representative of the UK population in terms of demographics and patient characteristics. The use of coded data mitigates the effect of recall and responder bias. To mitigate the effect of selection bias, all individuals with a lower GI symptom record in primary care have been included. Primary care data were linked to secondary care hospital records for all symptomatic patients, so it is likely that all IBD patients with a symptom coded in primary care have been included, minimizing ascertainment bias.

Important limitations of our study included that patients with or without an IBD diagnosis with no record of symptoms in primary care could not be included. It is known that symptoms may not be coded in primary care records but instead included in free-text comments which were not available in this study.^48^ Although we included a family history of IBD within our models, this is often not well documented in electronic health records data. This model is not applicable to the pediatric population, as they may present with different symptoms including poor growth or failure to thrive and thus require different risk models that have been specifically developed in pediatric cohorts. Other causes of chronic diarrhea, such as celiac disease, are important differential diagnoses. The models’ ability to distinguish between IBD and these conditions was not specifically evaluated. Ethnicity and deprivation are not included as predictors as there was a high level of missing data in the validation dataset. Also, the regression coefficients for ethnicity in the preliminary analysis suggested that certain ethnic minority groups were at lower risk of having IBD when this could potentially be due to underrepresentation of these groups in the observational data; inclusion of ethnicity as a predictor variable might therefore exacerbate existing disparities in the IBD diagnostic pathway for these minority groups. Therefore, it was decided to exclude ethnicity from the analysis. The blood and stool tests were used as categorical variables as there were less than 10% of patients with a recorded blood or stool test around the symptom. Due to this low recording rate, a missing category was used, necessitating the use of categorical variables.

The sensitivity and specificity of models at different risk thresholds could not be examined due to the potential for bias when estimating the metrics. This is because in survival analysis, some subjects may be censored, meaning their event status is unknown after a certain period and sensitivity and specificity do not handle censored data well.^49^ We developed separate models with and without FC testing to assess differences in model performance. However, only a small proportion of the study population underwent FC testing; this may partly explain why the models did not show significant variation in predictive accuracy. A higher rate of IBD was observed in those who underwent FC testing as primary care clinicians are more likely to request FC tests in those with a greater pretest probability of IBD. A broader implementation of FC testing in the at-risk population may improve the diagnostic accuracy of our models.

The diagnostic accuracy of our risk models cannot be directly compared to FC testing in primary care due to the lack of studies evaluating FC testing’s diagnostic accuracy in such settings. Further research is required to assess the diagnostic accuracy of FC testing in primary care, enabling more meaningful comparisons. We anticipate that FC test data will eventually be integrated with other variables in our risk models, providing a more comprehensive and holistic risk assessment for IBD.

## 5. Conclusions

To our knowledge, this is the first model predicting a composite diagnosis of IBD and separately for UC and CD in patients with suggestive symptomatology in primary care. The model could be used in patients presenting to primary care with GI symptoms to identify those at risk of IBD, who should go on to receive FC testing. Additionally, the model could be used for patients who fail to return their FC test or for those with inconclusive FC test results, providing primary care clinicians with additional information to inform their referral decision-making. The model can help primary care clinicians to ensure that patients at high risk of IBD are prioritized for further testing or referral, while also reducing the number of patients at low risk of IBD referred for invasive investigation in secondary care.

## Supplementary Material

jjaf044_suppl_Supplementary_Figure_S1

jjaf044_suppl_Supplementary_Figure_S2

jjaf044_suppl_Supplementary_Tables_S1-S8

jjaf044_suppl_Supplementary_Checklist

## Data Availability

Data used in this study were obtained under license from Clinical Practice Research Datalink (CPRD) and are not publicly available. Pseudonymized participant data are available from CPRD subject to Research Data Governance approval.
