# Supplementary material for: Development and validation of a risk prediction tool for the diagnosis of inflammatory bowel disease in patients presenting in primary care with abdominal symptoms
Source: J Crohns Colitis. 2025 Mar 18;19(4):jjaf044. doi: 10.1093/ecco-jcc/jjaf044 (PMC12010163; doi:10.1093/ecco-jcc/jjaf044)
Supplement: jjaf044_suppl_Supplementary_Figure_S2 [file jjaf044_suppl_supplementary_figure_s2.docx]

**
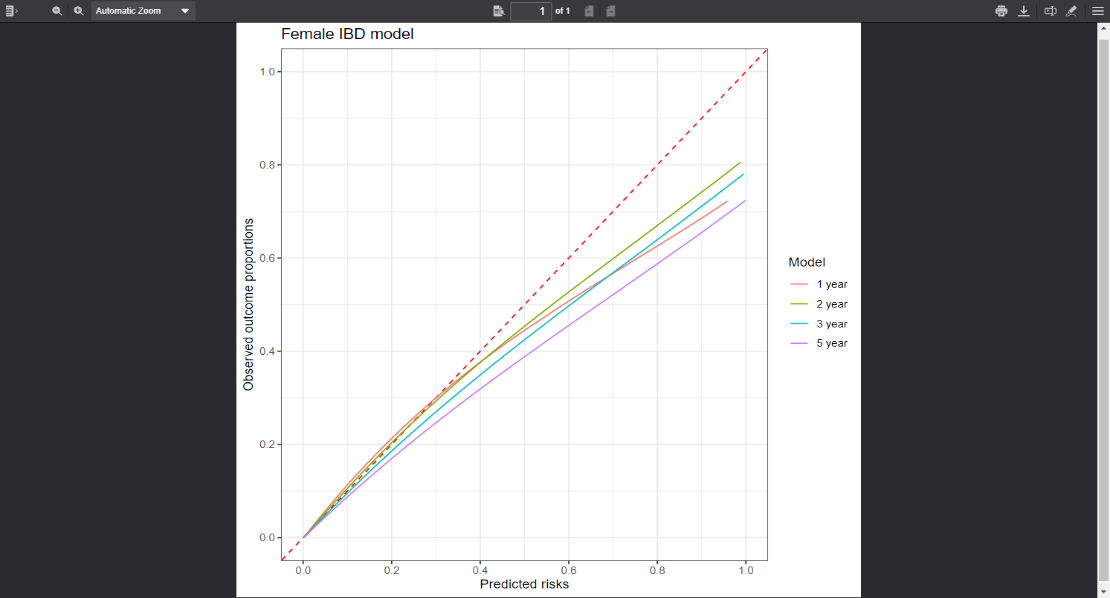
**
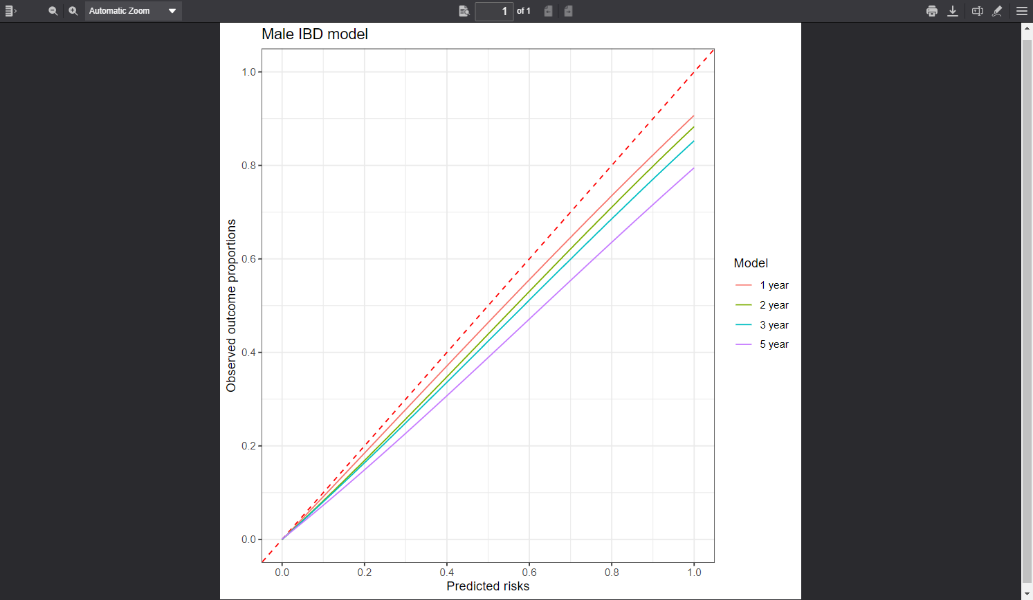
**Female IBD model Male IBD model**


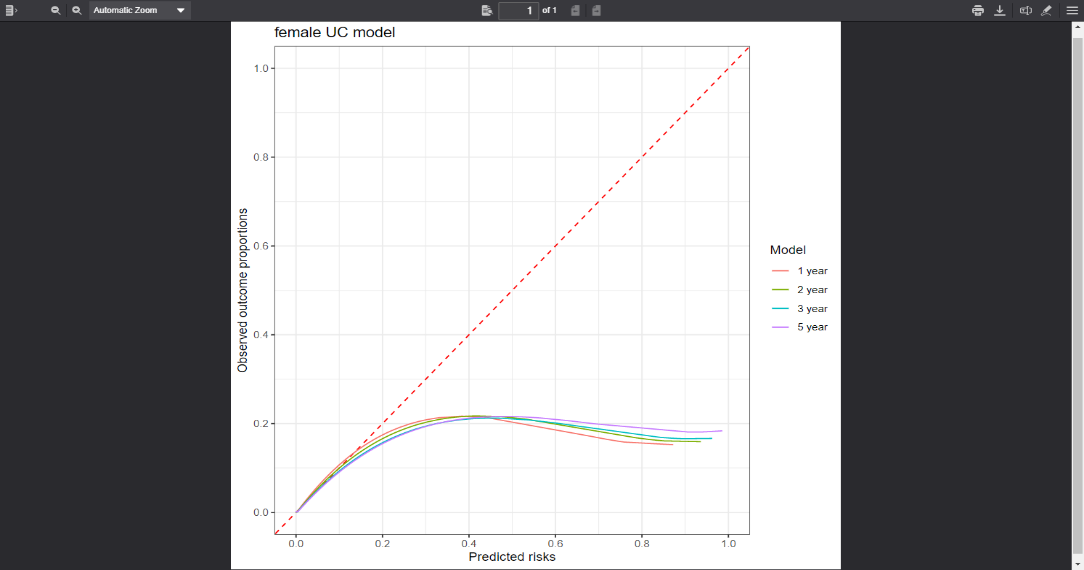

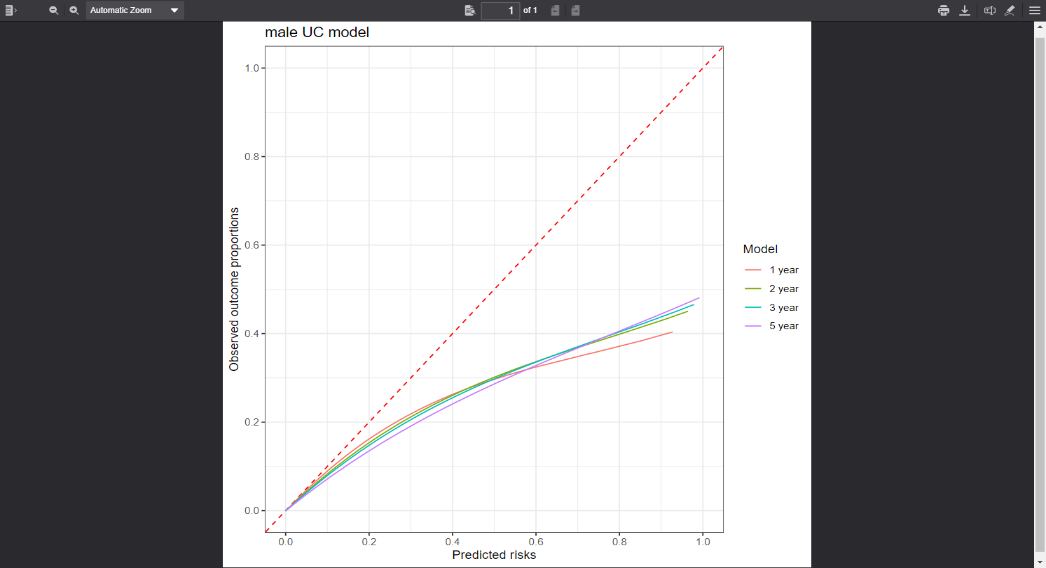
**Female UC model Male UC model**

**
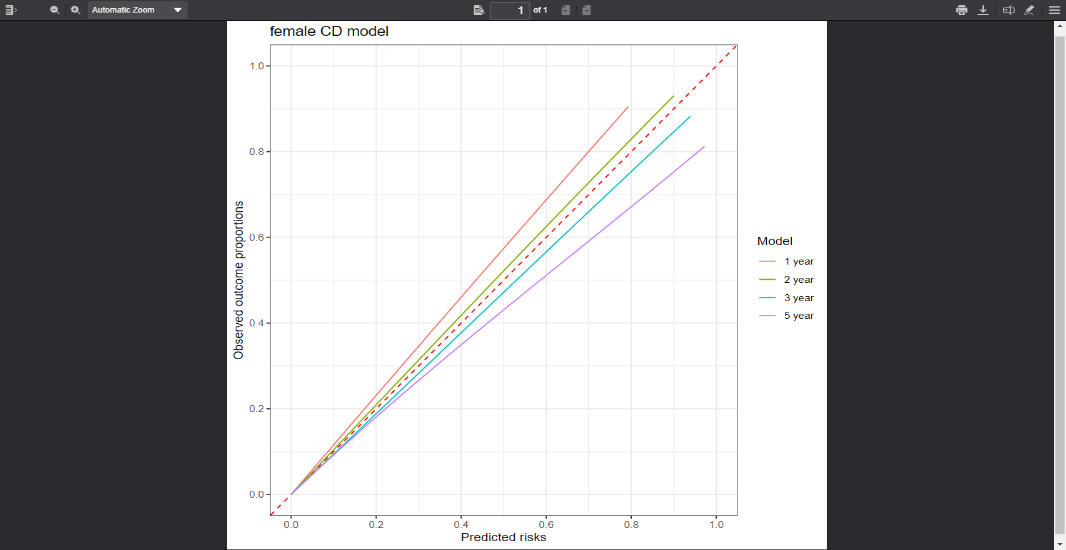

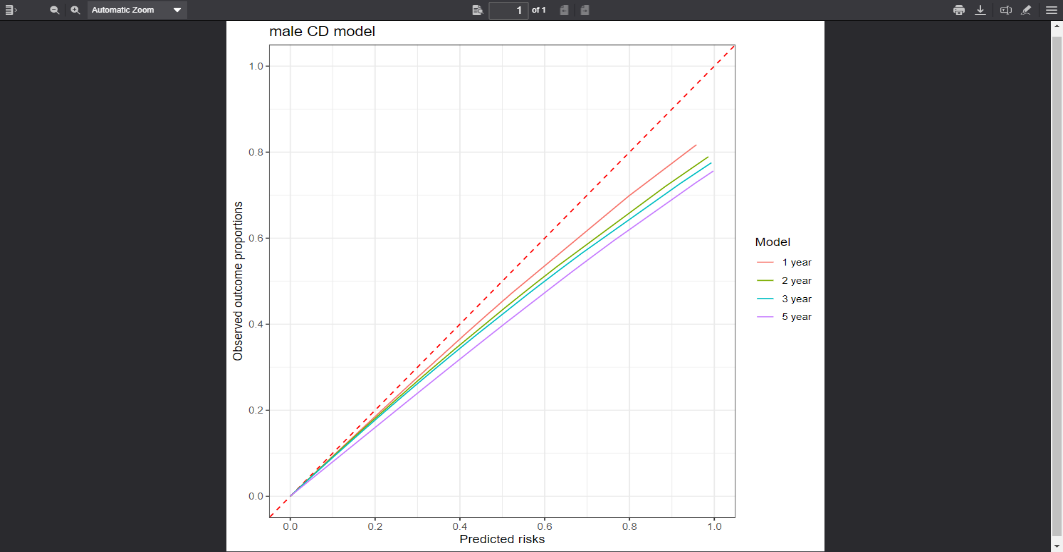
Female CD model Male CD model**

**Supplementary Figure 2: Calibration plots for IBD , ulcerative colitis (UC) and Crohn’s disease (CD) with faecal calprotectin in validation cohort at 1 , 2 , 3 and 5 year.**
