# Supplementary material for: Development and validation of a risk prediction tool for the diagnosis of inflammatory bowel disease in patients presenting in primary care with abdominal symptoms
Source: J Crohns Colitis. 2025 Mar 18;19(4):jjaf044. doi: 10.1093/ecco-jcc/jjaf044 (PMC12010163; doi:10.1093/ecco-jcc/jjaf044)
Supplement: jjaf044_suppl_Supplementary_Tables_S1-S8 [file jjaf044_suppl_supplementary_tables_s1-s8.docx]

**Supplementary file**

**Supplementary Table 1: Code lists**

**Ulcerative colitis**

| **DESCRIPTION** | **SNOMED CT CODE** |
| --- | --- |
| Ulcerative colitis | 64766004 |
| H/O: ulcerative colitis | 275549008 |
| Ulcerative proctitis | 52231000 |
| Exacerbation of ulcerative colitis | 414156000 |
| Ulcerative pancolitis | 444548001 |
| [RFC] Ulcerative colitis | 906191000006109 |
| Ulcerative proctocolitis NOS | 295046003 |
| UC - Ulcerative colitis | 64766004 |
| Ulcerative colitis confined to rectum | 52231000 |
| Chronic ulcerative proctitis | 52231000 |
| Chronic ulcerative recto sigmoiditis | 52506002 |
| UC - Ulcerative colitis confined to rectum | 52231000 |
| History of ulcerative colitis | 275549008 |
| Arthropathy in ulcerative colitis | 201727001 |
| Ulcerative proctosigmoiditis | 52506002 |
| Ulcerative colitis and/or proctitis | 295046003 |
| Chronic ulcerative proctosigmoiditis | 52506002 |
| Ulcerative recto sigmoiditis | 52506002 |
| [X]Other ulcerative colitis | 64766004 |
| Ulcerative proctocolitis | 295046003 |
| Juvenile arthritis in ulcerative colitis | 201807008 |
| Ulcerative (chronic) enterocolitis | 235714007 |
| Ulcerative colitis confined to rectum and sigmoid colon | 52506002 |

| **DESCRIPTION** | **READ CODE** |
| --- | --- |
| Ulcerative colitis | J410100 |
| Ulcerative colitis and/or proctitis | J41..12 |
| H/O: ulcerative colitis | 14C4.11 |
| Ulcerative proctocolitis | J410.00 |
| Ulcerative proctitis | J410300 |
| Arthropathy in ulcerative colitis | N031000 |
| Exacerbation of ulcerative colitis | J410400 |
| Ulcerative rectosigmoiditis | J410200 |
| Ulcerative (chronic) enterocolitis | J411.00 |
| Ulcerative proctocolitis NOS | J410z00 |
| [X]Other ulcerative colitis | Jyu4100 |
| Juvenile arthritis in ulcerative colitis | N045400 |
| Ulcerative pancolitis | J413.00 |

**Crohn’s Disease**

| **DESCRIPTION** | **SNOMED CT CODE** |
| --- | --- |
| Crohn's disease | 34000006 |
| Orofacial Crohn's disease | 196578009 |
| Arthropathy in Crohn's disease | 201728006 |
| Exacerbation of Crohn's disease of large intestine | 414153008 |
| Regional enteritis - Crohn | 34000006 |
| [RFC] Crohn’s disease | 906051000006102 |
| Crohn's disease of the small bowel NOS | 56689002 |
| [X]Other Crohn's disease | 34000006 |
| Crohn's disease NOS | 34000006 |
| Regional enteritis - Crohn's disease | 34000006 |
| Crohn's colitis | 50440006 |
| Crohn's disease of the terminal ileum | 196977009 |
| CD - Crohn's disease | 34000006 |
| Crohn disease of large bowel | 7620006 |
| Crohn disease of small intestine | 56689002 |
| Crohn disease of duodenum | 56287005 |
| Crohn’s disease, small intestine | 56689002 |
| Crohn disease of terminal ileum | 196977009 |
| Crohn’s disease, large intestine | 7620006 |
| Crohn's proctitis | 3815005 |
| Crohn disease of ileum | 38106008 |
| Crohn's disease of colon | 50440006 |
| Exacerbation of Crohn disease of large intestine | 414153008 |
| Crohn's disease of duodenum | 56287005 |
| Crohn’s disease, colon | 50440006 |
| Crohn disease of colon | 50440006 |
| Crohn's ileitis | 38106008 |
| Crohn's disease of rectum | 3815005 |
| Juvenile arthritis in Crohn's disease | 201805000 |
| Crohn's disease of the ileum unspecified | 38106008 |
| Crohn’s disease | 34000006 |
| Crohn's disease of oral soft tissues | 196578009 |
| Exacerbation of Crohn disease of small intestine | 414154002 |
| Crohn's disease of the large bowel NOS | 7620006 |
| Crohn's duodenitis | 56287005 |
| Arthropathy in Crohn disease | 201728006 |
| Juvenile arthritis in Crohn disease | 201805000 |
| Crohn disease of rectum | 3815005 |
| Crohn's disease of the ileum NOS | 38106008 |
| Oral Crohn's disease | 196578009 |
| Exacerbation of Crohn's disease of small intestine | 414154002 |
| Crohn disease | 34000006 |

| **DESCRIPTION** | **READ CODE** |
| --- | --- |
| Crohn's disease | J40..11 |
| Crohn's colitis | J401z11 |
| Crohn's disease of the small bowel NOS | J400z00 |
| Regional enteritis - Crohn's disease | J40..00 |
| Juvenile arthritis in Crohn's disease | N045300 |
| Arthropathy in Crohn's disease | N031100 |
| Crohn's disease of the large bowel NOS | J401z00 |
| Crohn's disease of the terminal ileum | J400200 |
| Orofacial Crohn's disease | J08z900 |
| Exacerbation of Crohn's disease of small intestine | J400500 |
| Exacerbation of Crohn's disease of large intestine | J401200 |
| Crohn's disease of the ileum NOS | J400400 |
| Crohn's disease NOS | J40z.11 |
| Crohn's disease of the ileum unspecified | J400300 |
| [X]Other Crohn's disease | Jyu4000 |

**ICD 10 Codes**

**IBD**

| K500 | Crohn's disease of small intestine |
| --- | --- |
| K501 | Crohn's disease of large intestine |
| K508 | Other Crohn's disease |
| K509 | Crohn's disease, unspecified |
| K51 | Ulcerative colitis |
| K510 | Ulcerative (chronic) enterocolitis |
| K511 | Ulcerative (chronic) ileocolitis |
| K512 | Ulcerative (chronic) proctitis |
| K513 | Ulcerative (chronic) recto sigmoiditis |
| K515 | Mucosal proctocolitis |
| K518 | Other ulcerative colitis |
| K519 | Ulcerative colitis, unspecified |
| K514 | Pseudo polyposis of colon |

**Symptoms**

**Nausea and vomiting**

| **DESCRIPTION** | **SNOMED-CT CODE** |
| --- | --- |
| Projectile vomiting | 8579004 |
| Habit vomiting | 49206006 |
| Retching | 84480002 |
| Nausea present | 162057007 |
| Vomit appearance - normal | 167827008 |
| Vomit odour | 167839000 |
| Vomit odour normal | 167840003 |
| Vomit pH | 992361000000108 |
| Pneumonitis due to inhalation of food or vomitus | 196032009 |
| Pneumonitis due to inhalation of vomitus | 196035006 |
| Persistent vomiting | 196746003 |
| Emesis - persistent | 196746003 |
| Vomit: pus present | 271354006 |
| C/O - vomiting | 272044004 |
| Vomiting blood - coffee ground | 40835002 |
| Functional vomiting | 37224001 |
| Morning nausea | 51885006 |
| Nausea | 422587007 |
| Nausea/vomiting | 960221000006103 |
| Frequency of vomiting | 1851581000006100 |
| Emesis | 422400008 |
| Vomiting of blood | 8765009 |
| Nausea NOS | 422587007 |
| Vomiting NOS | 300359004 |
| Vomit - O/E, general | 167820005 |
| Pneumonitis due to inhalation of food or vomitus NOS | 196032009 |
| Cyclical vomiting NOS | 18773000 |
| Persistent vomiting NOS | 196746003 |
| [D]Nausea and vomiting | 16932000 |
| [D]Vomiting | 422400008 |
| [D]Emesis | 422400008 |
| [D]Nausea and vomiting NOS | 16932000 |
| Vomiting | 300359004 |
| Vomit: faeculant | 300366003 |
| Vomiting | 300359004 |
| Nausea symptoms | 422587007 |
| Vomit odour faeculant | 167842006 |
| Vomiting - infective | 765480005 |
| Vomit: coffee ground | 300364000 |
| Faeculant vomit O/E | 275745004 |
| Vomit sample | 122572000 |
| Bilious vomit | 271352005 |
| Aspiration pneumonia caused by vomit | 196035006 |
| Coffee ground vomiting | 40835002 |
| Vomit odor | 167839000 |
| Bilious vomit O/E | 275744000 |
| N+V - Nausea and vomiting | 16932000 |
| Complaining of vomiting | 272044004 |
| Erosion of teeth due to persistent vomiting | 52031007 |
| Periodic vomiting | 18773000 |
| Odor of vomit | 167839000 |
| Pneumonitis caused by inhalation of vomitus | 196035006 |
| Coffee ground emesis | 40835002 |
| Vomit contains blood | 300361008 |
| Nauseous | 422587007 |
| Vomiting symptom | 249497008 |
| N&V - Nausea and vomiting | 16932000 |
| Aspiration of vomitus | 30227006 |
| Vomit odor normal | 167840003 |
| Blood in vomit O/E | 275786006 |
| Induction of vomiting | 133880003 |
| Cyclical vomiting syndrome | 18773000 |
| Vomit toxicology NOS | 250618007 |
| Vomitus specimen | 122572000 |
| Vomit: appearance NOS | 167826004 |
| Teeth erosion due to vomiting | 52031007 |
| Blood in vomit - symptom | 281102003 |
| Vomit: excessive acidity | 167846009 |
| Throwing up | 300359004 |
| [D]Retching | 84480002 |
| Vomiting symptoms | 249497008 |
| Observation of vomiting | 300359004 |
| Observation of nausea | 422587007 |
| Vomit odor offensive | 167841004 |
| Nauseated | 422587007 |
| Bilious emesis | 71419002 |
| Faeculent vomit O/E | 275745004 |
| Vomiting bile | 71419002 |
| Faeculant vomit O/E | 275745004 |
| [D]Nausea | 422587007 |
| Time since last episode of vomiting | 1851591000006100 |
| Reason for referral: Vomiting/Nausea | 1777091000006100 |
| Cyclical vomiting - psychogenic | 191970005 |
| Vomit odour offensive | 167841004 |
| Occult blood in vomit | 167849002 |
| [D]Projectile vomiting | 8579004 |
| [X]Psychogenic vomiting | 192450008 |
| C/O - nausea | 422587007 |
| Vomit: bilious | 271352005 |
| Coffee ground vomit | 300364000 |
| Hysterical vomiting | 37224001 |
| Vomit contains feces | 300366003 |
| Vomit: excessive alkalinity | 167847000 |
| Vomit odor feculent | 167842006 |
| Vomiting symptoms | 249497008 |
| Vomiting - bile stained | 71419002 |
| Psychogenic vomiting NOS | 37224001 |
| Frequency of vomiting | 866121000000105 |
| Vomiting blood - fresh | 267051003 |
| Vomit: undigested food present | 167835006 |
| Bilious vomit O/E | 275744000 |
| Blood in vomit O/E | 275786006 |
| Vomiting of blood | 8765009 |
| Vomit odour faeculant | 167842006 |
| Vomit odour NOS | 167839000 |
| Vomited meal | 301790009 |
| Effortless vomiting | 249500002 |
| Haematemesis - cause unknown | 308904008 |
| Vomiting food | 225586007 |
| Vomit | 1985008 |
| Intermittent vomiting | 236083006 |
| Intractable nausea and vomiting | 698861005 |
| Black vomit | 275371002 |
| Increased nausea and vomiting | 73335002 |
| Vomitus | 1985008 |

| **DESCRIPTION** | **READ CODE** |
| --- | --- |
| Vomiting | 1992 |
| Nausea | 198..00 |
| [D]Nausea | R070000 |
| Vomiting of blood | J680.11 |
| Cyclical vomiting - psychogenic | E264200 |
| Projectile vomiting | 1993 |
| [D]Nausea and vomiting | R070.00 |
| Psychogenic vomiting NOS | E275400 |
| Pneumonitis due to inhalation of food or vomitus | H470.00 |
| Vomiting symptoms | 199..14 |
| [D]Vomiting | R070100 |
| Nausea present | 1982 |
| Vomiting | 199..00 |
| [D]Projectile vomiting | R070400 |
| Nausea symptoms | 198..12 |
| Persistent vomiting | J162.00 |
| [X]Psychogenic vomiting | Eu50511 |
| Retching | 1997 |
| Morning nausea | 1983 |
| Coffee ground vomit | 4A24.11 |
| Nausea NOS | 198Z.00 |
| Cyclical vomiting NOS | J162000 |
| C/O - vomiting | 199..11 |
| [D]Nausea and vomiting NOS | R070z00 |
| [D]Emesis | R070200 |
| Vomit - O/E, general | 4A1..11 |
| Blood in vomit - symptom | 1994.1 |
| Vomit examination | 4A...00 |
| Bilious vomit O/E | 4A25.11 |
| [D]Retching | R070z12 |
| Emesis | 199..12 |
| Vomiting - bile stained | 1996 |
| Vomiting - infective | A0...13 |
| Aspiration pneumonia due to vomit | H470312 |
| Functional vomiting | J16y500 |
| Vomit: faeculant | 4A26.00 |
| C/O - nausea | 198..11 |
| Faeculant vomit O/E | 4A26.11 |
| Throwing up | 1992.1 |
| Pneumonitis due to inhalation of food or vomitus NOS | H470z00 |
| Persistent vomiting NOS | J162z00 |
| Vomit: excessive acidity | 4A42.00 |
| Vomiting NOS | 199Z.00 |
| Occult blood in vomit | 4A5..11 |
| Vomit: bilious | 4A25.00 |
| Vomit: coffee ground | 4A24.00 |
| Blood in vomit O/E | 4A23.11 |
| Vomiting blood - fresh | 1994 |
| Vomiting blood - coffee ground | 1995 |
| Pneumonitis due to inhalation of vomitus | H470300 |
| Habit vomiting | J162100 |
| Emesis - persistent | J162.11 |
| Vomit appearance - normal | 4A21.00 |
| Vomit odour | 4A3..00 |
| Teeth erosion due to vomiting | J013100 |
| Vomit: appearance NOS | 4A2Z.00 |
| Frequency of vomiting | 1999 |
| Vomit: undigested food present | 4A29.00 |

**2.Diarrhoea**

| **DESCRIPTION** | **SNOMED CT CODE** |
| --- | --- |
| Diarrhoea of presumed infectious origin | 43240000 |
| Diarrhoea | 62315008 |
| Epidemic diarrhoea | 86615009 |
| Viral diarrhoea | 111843007 |
| Diarrhoea and vomiting | 249519007 |
| Diarrhoea symptoms | 267060006 |
| Dietetic diarrhoea | 83134002 |
| Diarrhoea & vomiting | 854661000006104 |
| Reason for referral: Diarrhoea and Vomiting | 1776591000006100 |
| Manchester triage - Diarrhoea and vomiting | 1984091000006100 |
| Time since last episode of diarrhoea | 866211000000107 |
| Infectious diarrhoea NOS | 19213003 |
| Infectious diarrhoea | 19213003 |
| [X]Psychogenic diarrhoea | 231517009 |
| Loose stools | 398032003 |
| Travellers' diarrhoea | 11840006 |
| [X]Diarrhoea gastroenteritis of presumed infectious origin | 57419008 |
| Diarrhoea & vomiting -? infect | 19213003 |
| Diarrhoea - presumed non-infectious | 25374005 |
| Faeces consistency: semi-fluid | 398032003 |
| D+V - Diarrhoea and vomiting | 249519007 |
| Haemorrhagic diarrhoea | 95545007 |
| Observation of diarrhoea | 62315008 |
| Liquid faeces | 398212009 |
| Viral diarrhoea | 111843007 |
| Diarrhoea | 62315008 |
| Observation of diarrhoea | 62315008 |
| Chronic diarrhoea | 236071009 |
| Infective diarrhoea | 19213003 |
| Bloody diarrhoea | 95545007 |
| Allergic diarrhoea | 49237006 |
| Loose bowel movement | 398032003 |
| Dietetic diarrhoea | 83134002 |
| D - Diarrhoea | 62315008 |
| Presumed non-infectious diarrhoea | 69980003 |
| Liquid faeces | 398212009 |
| Diarrhoea of presumed infectious origin | 43240000 |
| Non-infective diarrhoea | 69980003 |
| Infectious diarrheal disease | 19213003 |
| D - Diarrhoea | 62315008 |
| Epidemic diarrhoea | 86615009 |
| D&V - Diarrhoea and vomiting | 249519007 |
| Diarrhoea and vomiting, symptom | 275297005 |
| Haemorrhagic diarrhoea | 95545007 |
| Traveller's diarrhoea | 11840006 |
| Bloody diarrhoea | 95545007 |
| Functional diarrhoea | 47812002 |
| Psychogenic diarrhoea | 268651002 |
| Chronic diarrhoea | 236071009 |
| Noninfective diarrhoea | 69980003 |
| Diarrhoea | 62315008 |
| Diarrhoea symptom | 267060006 |
| Loose faeces | 398032003 |
| Diarrhoea/loose stools | 982731000006108 |
| [RFC] Loose stools | 909311000006106 |
| Diarrhoea | 154931000033108 |
| Presumed non-infectious diarrhoea | 69980003 |
| Faeces consistency: fluid | 398212009 |
| Functional diarrhoea | 47812002 |
| Traveller’s diarrhoea | 11840006 |
| Psychogenic diarrhoea | 268651002 |
| Infective diarrhoea | 19213003 |
| Diarrhoea and vomiting | 249519007 |
| Time since last episode of diarrhoea | 1851601000006100 |
| Dysenteric diarrhoea | 46799006 |
| Allergic diarrhoea | 49237006 |
| Diarrhoea symptom NOS | 267060006 |
| Diarrhoea & vomiting, symptom | 275297005 |
| Loose faeces | 398032003 |
| Watery stool | 398212009 |
| Infectious diarrhoea | 19213003 |
| Dysenteric diarrhoea | 46799006 |

| **DESCRIPTION** | **READ CODE** |
| --- | --- |
| Diarrhoea | 19F..11 |
| Loose stools | 19F..12 |
| Dysenteric diarrhoea | A082000 |
| Diarrhoea & vomiting, symptom | 19FZ.11 |
| Diarrhoea | 19F2.00 |
| Infectious diarrhoea | A082.00 |
| Functional diarrhoea | J525.00 |
| Diarrhoea of presumed infectious origin | A083.00 |
| Diarrhoea symptoms | 19F..00 |
| Noninfective diarrhoea | J4...13 |
| Chronic diarrhoea | J43z.11 |
| Diarrhoea and vomiting | 19G..00 |
| Travellers' diarrhoea | A082.11 |
| Allergic diarrhoea | J432.11 |
| Diarrhoea & vomiting -? infect | A083.11 |
| Diarrhoea symptom NOS | 19FZ.00 |
| [D] Stools loose | R077100 |
| Viral diarrhoea | A076.11 |
| Psychogenic diarrhoea | E264300 |
| Diarrhoea - presumed non-infectious | J4zz.11 |
| Dietetic diarrhoea | J433.11 |
| Increased frequency of defaecation | 19EE.00 |
| Irritable bowel syndrome with diarrhoea | J521000 |
| Presumed noninfectious diarrhoea | J4z..11 |
| Faeces consistency: semi-fluid | 4743 |
| Epidemic diarrhoea | A082100 |
| Faeces consistency: fluid | 4744 |
| [X]Psychogenic diarrhoea | Eu45317 |
| Infectious diarrhoea NOS | A082z00 |
| [X]Diarrhoea+gastroenteritis of presumed infectious origin | Ayu0H00 |
| Time since last episode of diarrhoea | 19F5.00 |

**3. Rectal bleeding**

| DESCRIPTION | SNOMED CT CODE |
| --- | --- |
| Rectal bleeding | 12063002 |
| Blood in faeces symptom | 249624003 |
| Blood in faeces | 405729008 |
| Blood in stools | 961941000006107 |
| Referral to rectal bleeding clinic | 1779671000006100 |
| Altered blood in stools | 2901004 |
| Blood in stools altered | 405729008 |
| Bleeding PR | 12063002 |
| Blood in stool | 405729008 |
| GIB - Gastrointestinal bleeding | 74474003 |
| GIT - Gastrointestinal tract haemorrhage | 74474003.00 |
| PR - Bleeding per rectum | 12063002.00 |
| GIH - Gastrointestinal haemorrhage | 74474003.00 |
| Faeces: blood | 405729008.00 |
| RB - Rectal bleeding | 12063002.00 |
| GI - Gastrointestinal haemorrhage | 74474003.00 |
| Referral to rectal bleeding clinic | 809121000000102 |
| Gastrointestinal haemorrhage | 74474003 |
| Faeces: fresh blood present | 269900004 |
| Bright red blood in stool | 405729008 |
| Painless rectal bleeding | 414992000 |
| Blood in faeces | 405729008 |
| Painful rectal bleeding | 414991007 |
| Lower gastrointestinal haemorrhage | 87763006 |
| PRB - Rectal bleeding | 12063002 |
| Gastrointestinal tract haemorrhage NOS | 74474003 |
| Gastrointestinal haemorrhage unspecified | 74474003 |

| **DESCRIPTION** | **READ CODE** |
| --- | --- |
| Rectal bleeding | J573011 |
| GIB - Gastrointestinal bleeding | J68z.11 |
| Blood in stool | J681.11 |
| Gastrointestinal haemorrhage | J68..00 |
| Bleeding PR | J573.11 |
| Gastrointestinal tract haemorrhage NOS | J68zz00 |
| Blood in faeces | 19E6.00 |
| Blood in faeces symptom | 19E6.11 |
| PRB - Rectal bleeding | J573012 |
| Painless rectal bleeding | 196C.00 |
| Painful rectal bleeding | 196B.00 |
| Gastrointestinal haemorrhage unspecified | J68z.00 |
| Blood in faeces | 4762.11 |
| Blood in stools altered | J681.13 |
| Altered blood in stools | J681.12 |
| Faeces: fresh blood present | 4762 |
| Referral to rectal bleeding clinic | 8HTE000 |

**4. Mucus per rectum**

| **DESCRIPTION** | **SNOMED CT CODE** |
| --- | --- |
| Faeces: red currant jelly stool | 269901000 |
| Red currant jelly stool | 269901000 |
| Mucus in faeces | 271864008 |
| [D]Mucus in stool | 500261000000107 |
| Stool mucus abnormal [D] | 500261000000107 |
| [D]Mucus in stool | 271864008 |
| Colonic mucus | 47670000 |
| Rectal mucus | 21697007 |

| **DESCRIPTION** | **READ CODE** |
| --- | --- |
| [D]Mucus in stool | R121200 |
| Mucus in faeces | 19EH.00 |
| Red currant jelly stool | 4764.12 |
| Faeces:red currant jelly stool | 4764 |

**5. Bloating**

| **DESCRIPTION** | **SNOMED CT CODE** |
| --- | --- |
| Bloating symptom | 248490000 |
| Flatulence symptom | 308698004 |
| Abdomen feels bloated | 248490000 |
| [D]Flatulence, eructation and gas pain | 271832001 |
| [D]Bloating | 248490000 |
| [D]Flatulence, eructation and gas pain NOS | 271832001 |
| Flatulence/wind | 249504006 |
| [X]Psychogenic flatulence | 231517009 |
| Excessive flatulence | 80301007 |
| Bloat | 60728008 |
| Bloating | 60728008 |
| Bloated abdomen | 60728008 |
| [D]Flatulence | 249504006 |
| Functional bloating | 722879009 |
| [D]Flatulence NOS | 400221000000109 |
| Abdominal bloating | 116289008 |

| **DESCRIPTION** | **READ CODE** |
| --- | --- |
| Abdomen feels distended | 19A3.00 |
| [D]Bloating | R073400 |
| Bloating symptom | 19B..12 |
| Excessive flatulence | 19B2.00 |
| [D]Flatulence | R073000 |
| Flatulence symptom | 19B..14 |
| [D]Flatulence, eructation and gas pain | R073.00 |
| Flatulence/wind | 19B..00 |
| Abdomen feels bloated | 19A2.00 |
| [D]Flatulence, eructation and gas pain NOS | R073z00 |
| [X]Psychogenic flatulence | Eu45320 |

**6. Abdominal pain**

| DESCRIPTION | SNOMED CT CODE |
| --- | --- |
| Abdominal discomfort | 43364001 |
| Lower abdominal pain | 54586004 |
| Epigastric pain | 79922009 |
| Upper abdominal pain | 83132003 |
| Generalised abdominal pain | 102614006 |
| Abdominal wall pain | 162042000 |
| Right subcostal pain | 162048001 |
| Left flank pain | 162049009 |
| Right flank pain | 162050009 |
| Left iliac fossa pain | 162052001 |
| Suprapubic pain | 162053006 |
| C/O pelvic pain | 162147009 |
| O/E - umbilical pain on palp. | 163218001 |
| O/E - guarding - umbilical | 163230007 |
| O/E - guarding - hypogastrium | 163233009 |
| O/E - rebound tenderness | 163236001 |
| Iliac fossa pain | 247354009 |
| Flank pain | 247355005 |
| Abdominal pain type | 247358007 |
| Site of GIT pain | 267050002 |
| O/E - abdominal pain on palpation | 268941000 |
| Right upper quadrant pain | 301717006 |
| Type of GIT pain | 270477000 |
| Colicky abdominal pain present | 958291000006108 |
| Colicky abdominal pain absent | 958301000006109 |
| Cramping/abdominal discomfort | 961961000006106 |
| [D]Umbilical pain | 88522004 |
| Manchester triage - Abdominal pain in adult | 1983911000006100 |
| Manchester triage - Abdominal pain in child | 1983921000006100 |
| Type of GIT pain NOS | 270477000 |
| Site of GIT pain NOS | 267050002 |
| O/E -abdomen pain on palpation NOS | 268941000 |
| O/E -guarding on palpation NOS | 163224007 |
| O/E - rebound tenderness NOS | 163236001 |
| O/E - abdominal rigidity NOS | 163248003 |
| [D]Flatulence, eructation and gas pain | 271832001 |
| [D]Abdominal cramps | 51197009 |
| [D]Epigastric pain | 79922009 |
| [D]Pain in right iliac fossa | 162051008 |
| [D]Acute abdomen | 9209005 |
| [D]Other specified abdominal pain | 21522001 |
| [D]Abdominal pain NOS | 21522001 |
| [D]Abdominal rigidity | 72300008 |
| Type of GIT pain - symptom | 270477000 |
| [D]Nonspecific abdominal pain | 304542004 |
| [D]Right upper quadrant pain | 301717006 |
| O/E - abdominal pain - epigastrium | 163215003 |
| O/E - abd. pain - R.lumbar | 163217006 |
| O/E - abd. pain - umbilical | 163218001 |
| O/E - abd. pain - L.lumbar | 163219009 |
| O/E - abd. pain - R.iliac | 163220003 |
| O/E - abd. pain - hypogastrium | 163221004 |
| O/E - abd. pain - L.iliac | 163222006 |
| O/E - guarding-R.hypochondrium | 163226009 |
| O/E - guarding-L.hypochondrium | 163228005 |
| O/E - guarding - R.lumbar | 163229002 |
| O/E - guarding - L.lumbar | 163231006 |
| O/E - guarding - R.iliac | 163232004 |
| O/E - guarding - L.iliac | 163234003 |
| O/E - rebound - L.iliac | 163246004 |
| Colicky abdominal pain | 9991008 |
| On examination - abdominal rigidity | 163248003 |
| Type of abdominal pain | 247358007 |
| On examination - guarding - left iliac | 163234003 |
| O/E - rebound - R.lumbar | 163241009 |
| O/E - abd. pain - umbilical | 163218001 |
| On examination - abdominal pain - umbilical | 163218001 |
| On examination - rebound - right hypochondrium | 163238000 |
| On examination - guarding - left hypochondrium | 163228005 |
| O/E - rebound - L.lumbar | 163243007 |
| O/E - abd. pain - L.lumbar | 163219009 |
| On examination - guarding - left lumbar | 163231006 |
| On examination - abdominal pain - left lumbar | 163219009 |
| O/E - guarding - L.iliac | 163234003 |
| AP - Abdominal pain | 21522001 |
| On examination - guarding - hypogastrium | 163233009 |
| O/E - rebound - L.iliac | 163246004 |
| O/E - guarding-L.hypochondrium | 163228005 |
| O/E - rebound-R.hypochondrium | 163238000 |
| O/E - rebound - R.iliac | 163244001 |
| On examination - rebound - umbilical | 163242002 |
| Acute abdominal pain syndrome | 9209005 |
| O/E - abd. pain - R.iliac | 163220003 |
| Generalized abdominal pain | 102614006 |
| On examination - guarding on palpation | 163224007 |
| Complaining of perineal pain | 162148004 |
| On examination - rebound-left hypochondrium | 163240005 |
| LIF - Left iliac fossa pain | 162052001 |
| On examination - abdominal pain - hypogastrium | 163221004 |
| O/E - guarding - R.lumbar | 163229002 |
| O/E - guarding - L.lumbar | 163231006 |
| Spasmodic abdominal pain | 9991008 |
| O/E - abd.pain-L.hypochondrium | 163216002 |
| On examination - rebound - left iliac | 163246004 |
| On examination - board-like abdominal rigidity | 268942007 |
| O/E - guarding - R.iliac | 163232004 |
| O/E - abd. pain - L.iliac | 163222006 |
| On examination - guarding - epigastrium | 163227000 |
| O/E - abd. pain - epigastrium | 163215003 |
| On examination - rebound - right iliac | 163244001 |
| O/E - abd. pain - R.lumbar | 163217006 |
| On examination - rebound tenderness | 163236001 |
| Observation of abdominal rigidity | 72300008 |
| O/E - abd.pain-R.hypochondrium | 163214004 |
| On examination - abdominal pain - left iliac | 163222006 |
| On examination - guarding - right hypochondrium | 163226009 |
| O/E - rebound-L.hypochondrium | 163240005 |
| O/E - epigastric pain on palp. | 308903002 |
| Abdominal pain score | 1858471000006100 |
| Colicky abdominal pain control | 958281000006105 |
| No abdominal pain | 162037008 |
| Non-colicky abdominal pain | 162038003 |
| O/E - guarding on palpation | 163224007 |
| O/E - guarding - epigastrium | 163227000 |
| O/E - rebound - hypogastrium | 163245000 |
| [D]Abdominal pain | 21522001 |
| [D]Abdominal colic | 9991008 |
| O/E - board like abd. rigidity | 268942007 |
| O/E - rebound-R.hypochondrium | 163238000 |
| O/E - rebound - R.iliac | 163244001 |
| O/E - epigastric pain on palp. | 308903002 |
| On examination - guarding - right iliac | 163232004 |
| On examination - abdominal pain - epigastrium | 163215003 |
| On examination - abdominal pain - right hypochondrium | 163214004 |
| On examination - rebound - epigastrium | 163239008 |
| [D]Suprapubic pain | 162053006 |
| General abdominal pain-symptom | 102614006 |
| Subcostal pain | 247352008 |
| Site of abdominal pain | 247353003 |
| Abdominal pain in pregnancy | 309737007 |
| O/E - rebound - epigastrium | 163239008 |
| O/E - abdominal rigidity | 163248003 |
| [D]Functional abdominal pain syndrome | 449890002 |
| O/E - abd.pain-R.hypochondrium | 163214004 |
| O/E - abd.pain-L.hypochondrium | 163216002 |
| RIF - Right iliac fossa pain | 162051008 |
| O/E - abd. pain - hypogastrium | 163221004 |
| On examination - guarding - umbilical | 163230007 |
| O/E - guarding-R.hypochondrium | 163226009 |
| On examination - rebound - hypogastrium | 163245000 |
| [D]Pain in left iliac fossa | 162052001 |
| On examination - abdominal pain - left hypochondrium | 163216002 |
| On examination - epigastric pain on palpation | 308903002 |
| Central abdominal pain | 162046002 |
| Left subcostal pain | 162047006 |
| Right iliac fossa pain | 162051008 |
| O/E - guarding of abdomen | 163224007 |
| O/E - rebound - umbilical | 163242002 |
| Abdominal pain | 21522001 |
| [D]Recurrent acute abdominal pain | 271858001 |
| [X]Pain localized to other parts of lower abdomen | 54586004 |
| [X]Other and unspecified abdominal pain | 21522001 |
| Colicky abdominal pain | 9991008 |
| On examination - abdominal pain - right iliac | 163220003 |
| On examination - no abdominal pain on palpation | 163213005 |
| [D]Upper abdominal pain | 83132003 |
| O/E - rebound-L.hypochondrium | 163240005 |
| O/E - rebound - R.lumbar | 163241009 |
| O/E - rebound - L.lumbar | 163243007 |
| On examination - abdominal pain - right lumbar | 163217006 |
| On examination - guarding - right lumbar | 163229002 |
| On examination - rebound - right lumbar | 163241009 |
| On examination - rebound - left lumbar | 163243007 |
| Stomach ache | 271681002 |
| Rebound tenderness of left iliac fossa | 301420004 |
| Recurrent abdominal pain | 439469002 |
| C/O left iliac fossa pain | 274278000 |
| Rebound tenderness of right iliac fossa | 301419005 |
| O/E - epigastric pain | 274288004 |
| Left sided abdominal pain | 285387005 |
| Chronic abdominal pain | 111985007 |
| Tummy ache | 271681002 |
| Acute exacerbation of chronic abdominal pain | 444746004 |
| Right sided abdominal pain | 285388000 |
| Guarding of left iliac fossa | 300418006 |
| O/E - abdominal pain | 274287009 |
| Abdominal pain in early pregnancy | 314041007 |
| Chronic nonspecific abdominal pain | 235841007 |
| Guarding of right iliac fossa | 300417001 |
| C/O right iliac fossa pain | 274277005 |
| Unexplained abdominal pain | 314212008 |
| Abdominal pain through to back | 74704000 |
| Guarding of epigastrium | 300412007 |
| Acute abdominal pain | 116290004 |
| Psychosomatic abdominal pain | 307199009 |
| Abdominal pain - cause unknown | 314212008 |
| Burning epigastric pain | 21005005 |
| Complaining of right iliac fossa pain | 274277005 |
| Complaining of left iliac fossa pain | 274278000 |
| Periumbilical pain | 443503005 |

| **DESCRIPTION** | **READ CODE** |
| --- | --- |
| Abdominal pain | 1969 |
| Epigastric pain | 1972 |
| [D]Epigastric pain | R090500 |
| [D]Abdominal cramps | R090400 |
| [D]Abdominal pain | R090.00 |
| Abdominal pain type | 196..11 |
| [D]Abdominal colic | R090100 |
| [D]Recurrent acute abdominal pain | R090E00 |
| Abdominal discomfort | 1968 |
| [D]Abdominal pain NOS | R090z00 |
| Upper abdominal pain | 197B.00 |
| Central abdominal pain | 1971 |
| Non-colicky abdominal pain | 1963 |
| Site of abdominal pain | 197..13 |
| Subcostal pain | 197..14 |
| Colicky abdominal pain | 1962 |
| [D]Upper abdominal pain | R090H00 |
| General abdominal pain-symptom | 197A.11 |
| O/E - abd. pain - R.iliac | 25C8.00 |
| O/E - abd.pain-R.hypochondrium | 25C2.00 |
| O/E -abd.pain on palpation NOS | 25CZ.00 |
| O/E - abdo. pain on palpation | 25C..00 |
| O/E - rebound tenderness | 25E..00 |
| O/E - abd. pain - epigastrium | 25C3.00 |
| [D]Nonspecific abdominal pain | R090N00 |
| O/E - epigastric pain on palp. | 25C..11 |
| O/E - abd. pain - L.iliac | 25CA.00 |
| Lower abdominal pain | 197C.00 |
| Left subcostal pain | 1973 |
| O/E - guarding of abdomen | 25D..11 |
| O/E - abd. pain - umbilical | 25C6.00 |
| Generalised abdominal pain | 197A.00 |
| Right subcostal pain | 1974 |
| O/E - abd. pain - L.lumbar | 25C7.00 |
| Abdominal wall pain | 1969000 |
| [D]Other specified abdominal pain | R090y00 |
| O/E - guarding on palpation | 25D..00 |
| O/E - guarding - R.iliac | 25D8.00 |
| O/E - abd. pain - R.lumbar | 25C5.00 |
| O/E - abd.pain-L.hypochondrium | 25C4.00 |
| O/E - guarding-R.hypochondrium | 25D2.00 |
| O/E - abd. pain - hypogastrium | 25C9.00 |
| O/E - guarding - epigastrium | 25D3.00 |
| [D]Abdominal rigidity | R094.00 |
| O/E - abdominal rigidity | 25F..00 |
| [X]Pain localized to other parts of lower abdomen | Ryu1000 |
| O/E - rebound - L.iliac | 25EA.00 |
| O/E - rebound - R.iliac | 25E8.00 |
| [X]Other and unspecified abdominal pain | Ryu1100 |
| O/E - guarding - hypogastrium | 25D9.00 |
| O/E - guarding - L.iliac | 25DA.00 |
| O/E - rebound tenderness NOS | 25EZ.00 |
| O/E - guarding-L.hypochondrium | 25D4.00 |
| O/E - board like abd. rigidity | 25F2.00 |
| O/E - rebound - umbilical | 25E6.00 |
| O/E - guarding - umbilical | 25D6.00 |
| O/E - rebound - epigastrium | 25E3.00 |
| O/E - rebound-R.hypochondrium | 25E2.00 |
| O/E - rebound - R.lumbar | 25E5.00 |
| O/E -guarding on palpation NOS | 25DZ.00 |
| O/E - abdominal rigidity NOS | 25FZ.00 |
| O/E - rebound-L.hypochondrium | 25E4.00 |
| O/E - rebound - hypogastrium | 25E9.00 |
| [D]Functional abdominal pain syndrome | R090P00 |
| O/E - guarding - R.lumbar | 25D5.00 |

**7. Change in bowel habit**

| **DESCRIPTION** | **SNOMED CT CODE** |
| --- | --- |
| Change in bowel habit | 88111009 |
| Change in bowel habit | 982741000006103 |
| [D]Change in bowel habit | 88111009 |

| **DESCRIPTION** | **READ CODE** |
| --- | --- |
| Change in bowel habit | 19EA.00 |
| [D]Change in bowel habit | R078.00 |

**8. Perianal symptoms**

| DESCRIPTION | SNOMED CT CODE |
| --- | --- |
| Ischiorectal abscess | 36046008 |
| Anal pain | 68653001 |
| Rectal pain | 77880009 |
| Perianal abscess | 82127005 |
| Pruritus ani | 90446007 |
| Perianal itch | 90446007 |
| C/O perineal pain | 162148004 |
| Drainage of perianal abscess | 174392009 |
| Rectal abscess | 197166005 |
| Anorectal pain | 197232005 |
| Anal abscess drained | 174392009 |
| Anal/rectal abscess | 75236001 |
| Incision and drainage of abscess | 914781000006104. |
| Rectal abscess | 197166005 |
| Ano-rectal abscess NOS | 75236001 |
| Ano-rectal fissure abscess | 197164008 |
| [D] Perineal pain | 225565007. |
| Ischiorectal abscess/fistula | 286977005 |
| Laying open of high fistula in ano | 174374001 |
| Laying open of low fistula in ano | 174373007 |
| Laying open of anal fistula NEC | 532541000000102 |
| Anal fistula excision | 532541000000102 |
| Anal fistula | 72779005 |
| Rectal fistula | 80736008 |
| Ileorectal fistula | 91448003 |
| [D]Pelvic and perineal pain | 274671002 |
| Perianal irritation | 90446007 |
| Ano-rectal fistula | 72779005 |
| Inter sphincteric fistula | 197155003 |
| Superficial anal fistula | 197155003 |
| Repair of rectal fistula | 21756005 |
| Ischiorectal abscess/fistula | 286977005 |
| Anal fistula operations | 275018007 |
| Anal fissure/fistula | 197150008 |
| Intern. Closure -rectal fistula | 21756005 |
| Perineal irritation | 281639001 |
| Closure of rectal fistula | 21756005 |
| Fistula-in-ano | 72779005 |
| Anal fistula operations | 275018007 |
| Repair of anal fistula using plug | 442289003 |
| Anal abscess drained | 287801001 |
| Incision and drainage of perirectal abscess | 37903002 |
| Incision of perianal abscess | 448395007 |
| Drainage of anorectal abscess | 235393003 |
| Anal abscess | 1082661000119100 |
| Drainage of recto anal abscess | 235393003 |
| Incision and drainage of perianal abscess | 48047006 |
| Incision and drainage of anorectal abscess | 275231009 |
| Incision and drainage of rectal abscess | 8324006 |

| **DESCRIPTION** | **READ CODE** |
| --- | --- |
| Ischiorectal abscess | J541.00 |
| Fistula-in-ano | J531.00 |
| Perianal abscess | J540.00 |
| Pruritus ani | M180.00 |
| Laying open of anal fistula NEC | 7738200 |
| Rectal pain | J574800 |
| Anorectal pain | J574F00 |
| Anal pain | J574700 |
| Drainage of perianal abscess | 773A100 |
| Excision of anal fistula | 7738611 |
| Laying open of low anal fistula | 7738000 |
| Perianal itch | M180.11 |
| Perineal irritation | M2yD.00 |
| [D] Perineal pain | R090G12 |
| Rectal abscess | J546.00 |
| Perianal irritation | M180.13 |
| [D]Pelvic and perineal pain | R090G00 |
| Rectal fistula | J531300 |
| Ano-rectal abscess NOS | J54z.00 |
| Anal fistula operations | 7738.11 |
| Ano-rectal fistula | J531200 |
| Closure of rectal fistula | 7729700 |
| Laying open of high anal fistula | 7738100 |
| Rectal abscess | J54..12 |
| Drainage of perirectal abscess | 773A200 |
| C/O perineal pain | 1A5A.00 |
| Ileorectal fistula | J57y500 |
| Ano-rectal fissure abscess | J544.00 |
| Incision and drainage of abscess | 7G25700 |
| Repair of anal fistula using plug | 7738900 |
| York - Mason repair of rectal fistula | 7725411 |

**9.Weight loss**

| **DESCRIPTION** | **SNOMED CT CODE** |
| --- | --- |
| Abnormal weight loss | 267024001 |
| Weight loss from baseline weight | 401003006 |
| Complaining of weight loss | 198511000000103 |
| Weight Loss | 161832001 |
| Unexplained/progressive weight loss | 960561000006106 |
| Percentage weight loss | 1708211000006100 |
| Malnutrition universal screening tool - weight loss score | 1995641000006100 |
| [D]Abnormal loss of weight | 267024001 |
| [D]Cachexia | 238108007 |
| [D]Cachexia NOS | 238108007 |
| Abnormal decrease in weight | 267024001 |
| Losing weight | 161832001 |
| Involuntary weight loss | 448765001 |
| Decreased BMI (body mass index) | 6497000 |
| Percentage weight loss | 248349002 |
| Weight decreasing | 161832001 |
| Progressive weight loss | 161832001 |
| Unintentional weight loss | 448765001 |
| Abnormal weight loss - symptom | 267024001 |
| Unexplained weight loss | 422868009 |
| Recent weight loss | 426977000 |
| Percentage underweight | 248355007 |
| Weight decreased | 262285001 |
| Excessive weight loss | 309257005 |
| Weight loss | 89362005 |

| **DESCRIPTION** | **READ CODE** |
| --- | --- |
| Weight decreasing | 1623 |
| [D]Abnormal loss of weight | R032.00 |
| Abnormal weight loss | 1625 |
| Abnormal weight loss - symptom | 1625.11 |
| Complaining of weight loss | 1D1A.00 |
| [D]Cachexia | R2y4.00 |
| Intentional weight loss | 1626 |
| Weight loss from baseline weight | 22A8.00 |
| [D]Cachexia NOS | R2y4z00 |
| Unintentional weight loss | 1627 |
| Percentage weight loss | 22A9.00 |

**10. Miscellaneous symptoms**

| **DESCRIPTION** | **SNOMED CT CODE** |
| --- | --- |
| Gastrointestinal symptoms NOS | 267045008 |
| Psychogenic gastrointestinal tract symptom NOS | 268650001 |
| Type of GIT pain - symptom | 270477000 |
| Psychogenic gastrointestinal tract symptoms | 268650001 |
| GIT symptoms | 267045008 |
| GIT symptom changes | 170671007 |
| Gastrointestinal symptom NOS | 267045008 |
| Gastrointestinal symptoms | 267045008 |
| Gastrointestinal symptoms reproduced | 251381007 |

| **DESCRIPTION** | **READ CODE** |
| --- | --- |
| GIT symptoms | 19...11 |
| Gastrointestinal symptoms | 19...00 |
| Gastrointestinal symptom NOS | 19ZZ.00 |
| Gastrointestinal symptoms NOS | 19Z..00 |
| Psychogenic gastrointestinal tract symptoms | E264.00 |
| GIT symptom changes | 6643 |
| Psychogenic gastrointestinal tract symptom NOS | E264z00 |

**Extraintestinal Manifestations**

1. **Mouth ulcers**

| **DESCRIPTION** | **SNOMED CT CODE** |
| --- | --- |
| O/E - mouth ulcer present | 163145007 |
| Major aphthous ulceration | 196531008 |
| Mouth ulcer | 26284000 |
| Oral aphthae | 426965005 |
| Recurrent aphthous ulceration | 398870000 |
| Aphthous ulcers - mouth ulcers | 426965005 |
| Adverse reaction to Preparations For Non-Specific Mouth Ulceration | 1010891000006100 |
| Adverse reaction to Adcortyl In Orabase | 1014781000006100 |
| Adcortyl In Orabase For Mouth Ulcer | 1179131000033100 |
| Adverse reaction to Adcortyl In Orabase For Mouth Ulcer | 1706691000006100 |
| Oral aphthae NOS | 426965005 |
| Recurrent mouth ulcers | 723177002 |
| Minor aphthous ulceration | 307772002 |
| Herpetic aphthous ulceration | 319297003 |
| Recurrent aphthous ulcer | 398870000 |
| Sutton's aphthae | 196535004 |
| Ulceration of oral mucosa | 26284000 |
| Mouth ulceration | 26284000 |
| Recurrent oral ulceration | 723177002 |
| Recurrent ulcer of mouth | 723177002 |
| Herpetiform aphthae | 319297003 |
| MJAU - Major aphthous ulceration | 196531008 |
| Aphthae minor | 307772002 |
| On examination - mouth ulcer present | 163145007 |
| Ulcer of mouth | 26284000 |
| Oral herpetiform aphthous ulceration | 319297003 |
| Minor oral aphthous ulceration | 307772002 |
| Aphthous stomatitis | 426965005 |
| Oral ulcer | 26284000 |
| Adcortyl In Orabase | 104731000033100 |
| O/E - mouth ulcer | 163145007 |
| Aphthous stomatitis | 426965005 |
| Herpetiform aphthous stomatitis | 319297003 |
| Aphthous ulcer | 427617000 |
| Recurrent aphthous stomatitis | 722781002 |
| Aphthous ulceration | 427617000 |

| **Description** | **Read Code** |
| --- | --- |
| Mouth ulcer | J082.11 |
| Oral aphthae | J082.00 |
| Aphthous stomatitis | J082400 |
| O/E - mouth ulcer | 2533.11 |
| O/E - mouth ulcer present | 2533 |
| Oral aphthae NOS | J082z00 |
| Minor aphthous ulceration | J082000 |
| Recurrent aphthous ulceration | J082200 |
| Recurrent mouth ulcers | J082211 |
| Major aphthous ulceration | J082100 |
| Herpetic aphthous ulceration | J082300 |

1. **Ophthalmic Extraintestinal manifestations**

| **DESCRIPTION** | **SNOMED CT CODE** |
| --- | --- |
| Recurrent iridocyclitis | 6869001 |
| Primary iridocyclitis | 12630008 |
| Anterior scleritis | 63454000 |
| Scleritis | 78370002 |
| Posterior scleritis | 267660007 |
| Chronic anterior uveitis | 398155003 |
| Scleritis/episcleritis | 267659002 |
| Posterior uveitis NOS | 43363007 |
| Unspecified acute iridocyclitis | 267618008 |
| Unspecified subacute iridocyclitis | 267618008 |
| Chronic iridocyclitis NOS | 77971008 |
| Certain types of iridocyclitis | 77971008 |
| Scleritis or episcleritis NOS | 267659002 |
| [X]Other iridocyclitis | 77971008 |
| [X]Iridocyclitis in other diseases classified elsewhere | 77971008 |
| Uveitis NOS | 128473001 |
| Secondary noninfected iridocyclitis | 193488003 |
| Chronic iridocyclitis due to another disorder | 193493000 |
| Chronic iritis | 398155003 |
| Diffuse scleritis | 91612009 |
| Secondary non-infected iridocyclitis | 193488003 |
| Diffuse uveitis | 75614007 |
| Intermediate uveitis | 314429009 |
| Unspecified acute iridocyclitis | 619301000000100 |
| Acute iritis (iridocyclitis) | 619301000000100 |
| Acute and subacute iridocyclitis | 267618008 |
| Pan uveitis | 75614007 |
| Brawny scleritis | 91612009 |
| Unspecified chronic iridocyclitis | 77971008 |
| Iritis | 65074000 |
| Acute iritis (iridocyclitis) | 267618008 |
| Scleritis and episcleritis | 267659002 |
| Chronic iridocyclitis due to disease EC | 193493000 |
| Anterior uveitis | 410692006 |
| Unspecified iridocyclitis | 77971008 |
| [X]Scleritis episcleritis in diseases CE | 231872000 |
| Acute or subacute iritis NOS | 267618008 |
| Unspecified scleritis | 78370002 |
| Episcleritis | 815008 |
| Episcleritis periodica fugax | 31166000 |
| Nodular episcleritis | 70558001 |
| Chronic iridocyclitis | 314429009 |
| Acute anterior uveitis of right eye | 336541000119107 |
| Anterior uveitis idiopathic | 231947004 |
| Idiopathic pan uveitis | 766933000 |
| Acute iritis of left eye | 342141000119102 |
| Diffuse episcleritis | 314549003 |
| Nodular scleritis | 95195003 |
| AAU - acute anterior uveitis | 4927003 |
| History of uveitis | 16098861000119100 |
| Chronic uveitis | 444248002 |
| Acute anterior uveitis of left eye | 342151000119100 |
| Simple episcleritis | 231873005 |
| Acute anterior uveitis | 4927003 |
| Idiopathic anterior uveitis | 231947004 |
| Acute anterior uveitis of bilateral eyes | 347311000119100 |
| Acute anterior uveitis of both eyes | 347311000119100 |
| Nodular episcleritis of right eye | 333651000119103 |

| **DESCRIPTION** | **READ CODE** |
| --- | --- |
| Episcleritis | F4K0.11 |
| Uveitis NOS | F443.11 |
| Iritis - acute | F440.11 |
| Anterior uveitis | F443000 |
| Scleritis | F4K0.12 |
| Chronic anterior uveitis | F441200 |
| Nodular episcleritis | F4K0200 |
| Acute or subacute iritis NOS | F440z00 |
| H/O: iritis | 1486 |
| Acute and subacute iridocyclitis | F440.00 |
| Posterior uveitis NOS | F432200 |
| Chronic iritis | F441.11 |
| Recurrent iridocyclitis | F440300 |
| Panuveitis | F401100 |
| Posterior scleritis | F4K0700 |
| Unspecified scleritis | F4K0000 |
| Chronic iridocyclitis | F441.00 |
| Unspecified chronic iridocyclitis | F441000 |
| Anterior scleritis | F4K0300 |
| Unspecified acute iridocyclitis | F440000 |
| Scleritis or episcleritis NOS | F4K0z00 |
| Episcleritis periodica fugax | F4K0100 |
| Brawny scleritis | F4K0600 |
| Secondary noninfected iridocyclitis | F440500 |
| Unspecified subacute iridocyclitis | F440100 |
| Chronic iridocyclitis NOS | F441z00 |
| Primary iridocyclitis | F440200 |
| Certain types of iridocyclitis | F442.00 |
| Iritis | F443100 |
| [X]Other iridocyclitis | FyuDC00 |

1. **Dermatological extraintestinal manifestation**

| DESCRIPTION | SNOMED_CT_CODE |
| --- | --- |
| Pyoderma faciale | 29909004 |
| Guttate psoriasis | 37042000 |
| Pyoderma | 70759006 |
| Sweet's syndrome | 84625002 |
| H/O: psoriasis | 161562002 |
| Psoriasis annularis | 200962007 |
| Psoriasis diffusa | 200964008 |
| Psoriasis geographica | 200966005 |
| Psoriasis palmaris | 200970002 |
| Psoriasis plantaris | 200971003 |
| Pustular psoriasis | 200973000 |
| Psoriasis universalis | 200974006 |
| Erythrodermic psoriasis | 200977004 |
| Scalp psoriasis | 238608008 |
| Palmoplantar pustular psoriasis | 27520001 |
| Chronic large plaque psoriasis | 402307000 |
| Pyoderma NOS | 70759006 |
| Other psoriasis | 9014002 |
| Psoriasis NOS | 9014002 |
| Parapsoriasis unspecified | 88233000 |
| Other psoriasis and similar disorders | 399937000 |
| Psoriasis and similar disorders NOS | 9014002 |
| [X]Other psoriasis | 9014002 |
| [X]Erythema in other diseases classified elsewhere | 86735004 |
| Psoriasis discoidea | 200965009 |
| Psoriasis ostracea | 200969003 |
| Erythematous conditions | 86735004 |
| Psoriasis area and severity index | 866341000000101 |
| Erythema | 247441003 |
| Psoriasis vulgaris & (Chronic large plaque psoriasis) | 200975007 |
| Pustular psoriasis of the palms AND/OR soles | 27520001 |
| On examination - erythematous rash | 135888007 |
| Psoriasis area and severity index score | 866341000000101 |
| Ostraceous psoriasis | 200969003 |
| PASI (psoriasis area and severity index) score | 866341000000101 |
| Flexural psoriasis | 238600001 |
| Flexural psoriasis | 238600001 |
| Psoriasis gyrata | 200967001 |
| Psoriasis punctata | 200972005 |
| Erythema nodosum | 32861005 |
| Pyoderma gangrenosum | 74578003 |
| Psoriasis unspecified | 9014002 |
| Parapsoriasis NOS | 88233000 |
| [X]Other parapsoriasis | 88233000 |
| Sweet syndrome | 84625002 |
| Acute palmoplantar pustular psoriasis | 3533007 |
| Pustular psoriasis of palms and soles | 27520001 |
| Erythema | 982391000006108 |
| Psoriasis circinata | 200963002 |
| Psoriasis vulgaris | 200975007 |
| Guttate parapsoriasis | 10057001 |
| Chronic palmoplantar pustular psoriasis | 27520001 |
| Psoriasis and similar disorders | 9014002 |
| EN - Erythema nodosum | 32861005 |
| Chronic large plaque psoriasis | 1583831000006100 |
| Psoriasis inveterate | 200968006 |
| O/E - erythematous rash | 135888007 |
| PASI - psoriasis area and severity index | 866311000000102 |
| Chronic stable plaque psoriasis | 402310007 |
| Psoriasis area and severity index | 866311000000102 |
| Chronic small plaque psoriasis | 402308005 |
| Psoriasis and similar disorders | 200955003 |
| Localised pustular psoriasis | 81271001 |
| Generalised pustular psoriasis | 238612002 |
| Juvenile pustular psoriasis | 238615000 |
| Childhood pustular psoriasis | 402328009 |

| **DESCRIPTION** | **READ CODE** |
| --- | --- |
| Erythema nodosum | M152.00 |
| Pyoderma | M070.00 |
| Sweet's syndrome | M228.00 |
| Pyoderma gangrenosum | M070200 |
| Pyoderma NOS | M070z00 |

**4.Primary sclerosing cholangitis**

| **DESCRIPTION** | **SNOMED CT CODE** |
| --- | --- |
| PSC - Primary sclerosing cholangitis | 197441003 |
| Primary sclerosing cholangitis | 197441003 |
| **DESCRIPTION** | **READ CODE** |
| Primary sclerosing cholangitis | J661700 |

**5.Swollen joints**

| DESCRIPTION | SNOMED_CT_CODE |
| --- | --- |
| Intermittent hydrarthrosis | 711286009 |
| O/E - joint swelling | 164524001 |
| O/E - swelling - joint | 164524001 |
| Synovial fluid examination | 167859001 |
| Shoulder joint synovial fluid | 167868004 |
| Hip joint synovial fluid | 167873005 |
| Knee joint synovial fluid | 167874004 |
| Ankle joint synovial fluid | 167875003 |
| Foot joint synovial fluid | 167876002 |
| Synovial fluid viscosity | 167886001 |
| Synovial fluid viscosity high | 167888000 |
| Synovial fluid viscosity low | 167889008 |
| Synovial fluid fibrin clot | 167891000 |
| Synovial fluid: no fibrin clot | 167892007 |
| Synovial fluid: fibrin clot + | 167893002 |
| Synovial fluid cell count OK | 167896005 |
| Synovial fluid abnorm. content | 167899003 |
| Effusion of sternoclavicular joint | 202371002 |
| Effusion of acromioclavicular joint | 202372009 |
| Effusion of elbow | 202373004 |
| Wrist joint effusion | 202375006 |
| Effusion of wrist | 202375006 |
| Effusion of hip | 202379000 |
| Knee joint effusion | 202381003 |
| Effusion of knee | 202381003 |
| Ankle joint effusion | 202383000 |
| Effusion of subtalar joint | 202384006 |
| Chronic joint effusion | 202391009 |
| Acute joint effusion | 202392002 |
| Chronic crepitant synovitis of hand and wrist | 202925003 |
| Synovitis of hip | 202926002 |
| Synovitis of knee | 239817004 |
| Swollen knee | 248491001 |
| Joint effusion of the lower leg | 267942009 |
| Synovial fluid for organism | 269956000 |
| Swollen joint | 271771009 |
| Swollen foot | 297142003 |
| Synovial fluid sample | 119332005 |
| Swollen thumb | 314916002 |
| Joint effusion of the hand | 16711001 |
| Intermittent joint effusion | 50442003 |
| Effusion of shoulder | 40884005 |
| Hydrarthrosis | 387637008 |
| Swollen hand | 299037003 |
| Joint effusion of the forearm | 267940001 |
| Synovitis of shoulder | 239818009 |
| Effusion - shoulder joint | 40884005 |
| Effusion - hip joint | 9363005 |
| Effusion - ankle/foot | 4819006 |
| Effusion - multiple joint | 36662003 |
| Effusion - wrist joint | 202375006 |
| Immediate swelling of knee | 1.56646E+15 |
| Synovial fluid mucin clot test | 9.96611E+14 |
| Synovial fluid specimen | 1.71714E+15 |
| Test request : Synovial fluid for organism | 1.89938E+15 |
| O/E - joint swelling NOS | 164524001 |
| Synovial fluid exam. - general | 167859001 |
| Synovial fluid: joint NOS | 119332005 |
| Synovial fluid: appearance NOS | 167879009 |
| Synovial fluid viscosity NOS | 167886001 |
| Synovial fluid fibrin clot NOS | 167891000 |
| Synovial fluid cell count NOS | 60923009 |
| Synovial fluid exam. NOS | 167859001 |
| Joint effusion of unspecified site | 387637008 |
| Joint effusion of other specified site | 387637008 |
| [D]Synovial fluid abnormal | 167899003 |
| Joint effusion of the upper arm | 202373004 |
| Joint effusion of the pelvic region and thigh | 9363005 |
| Synovial fluid source | 119332005 |
| Effusion of joint | 387637008 |
| Synovial fluid sent for exam. | 167861005 |
| Synovitis of elbow | 239816008 |
| Swelling of joint - effusion | 387637008 |
| Synovitis NOS | 416209007 |
| Ankle swelling symptom | 267039000 |
| Effusion of DIP joint - finger | 202378008 |
| Effusion of IP joint of toe | 202389001 |
| Effusion of lesser MTP joint | 202388009 |
| Effusion of multiple joints | 36662003 |
| Effusion of PIP joint of finger | 202377003 |
| Effusion of tibio-fibular joint | 202382005 |
| Finger swelling | 299060006 |
| Joint effusion of the ankle and foot | 4819006 |
| Synovial fluid: clear | 167880007 |
| Synovial fluid: turbid | 167884003 |
| Synovial fluid visc. normal | 167887005 |
| Synovial fluid: no abn.content | 167900008 |
| Effusion of joint of pelvic region | 9363005 |
| Synovial fluid: no abn.content | 167900008 |
| Transient synovitis of hip | 301864002 |
| Synovial fluid source | 119332005 |
| Synovial fluid yellow | 167881006 |
| Synovial fluid cell count high | 167897001 |
| Joint fluid cloudy | 167882004 |
| Effusion of sacroiliac joint | 202380002 |
| Synovial fluid turbid | 167884003 |
| Toe swelling | 277890004 |
| On examination - joint effusion present | 164526004 |
| Effusion of joint of ankle AND/OR foot | 4819006 |
| Synovial fluid cloudy | 167882004 |
| Joint fluid bloody | 269909003 |
| Joint fluid yellow | 167881006 |
| Synovial fluid examination - abnormal | 167863008 |
| Hand joint effusion | 16711001 |
| Shoulder joint effusion | 40884005 |
| Intermittent effusion of joint | 711286009 |
| Foot swelling | 297142003 |
| Joint fluid turbid | 167884003 |
| Synovial fluid: clear | 167880007 |
| On examination - joint swelling | 164524001 |
| Joint swelling | 271771009 |
| Effusion of joint of hand | 16711001 |
| Synovial fluid examination - normal | 167862003 |
| Swelling of hand | 299037003 |
| Synovial fluid exam. - normal | 167862003 |
| Synovial fluid: no abnormal content | 167900008 |
| Effusion of tibiofibular joint | 202382005 |
| Synov. fluid cell count high | 167897001 |
| Effusion of pelvis | 9363005 |
| Synovial fluid viscosity normal | 167887005 |
| Synovial fluid visc. normal | 167887005 |
| Swelling of first metatarsophalangeal joint of hallux | 415692008 |
| Joint effusion of other specified site | 6.80401E+14 |
| Effusion of joint of upper arm | 267939003 |
| Synovial fluid abn.content NOS | 167899003 |
| Effusion of other tarsal joint | 4819006 |
| Effusion - other joint | 6.80401E+14 |
| Effusion of joint of lower leg | 267942009 |
| Joint effusion of the upper arm | 267939003 |
| Effusion - elbow joint | 267939003 |
| Joint effusion of the forearm | 267940001 |
| Synovial fluid exam. gen. NOS | 167859001 |
| Right ankle swelling circumference | 1.83058E+15 |
| Effusion - knee joint | 202381003 |
| Ankle swelling | 267039000 |
| Synovial fluid - C/S | 275856007 |
| Swollen toe | 277890004 |
| Aspiration of fluid from knee joint | 281810004 |
| Shoulder synovitis | 239818009 |
| Synovial fluid: uric acid | 167903005 |
| Synovial fluid: blood stained | 269909003 |
| Effusion of MCP joint | 202376007 |
| Effusion of sacro-iliac joint | 202380002 |
| Synovial fluid: yellow | 167881006 |
| Synovial fluid: cloudy | 167882004 |
| Synov. fluid cell count high | 167897001 |
| Effusion into joint | 387637008 |
| Synovial fluid sent for examination | 167861005 |
| Synovial fluid: cloudy | 167882004 |
| Effusion - elbow joint | 202373004 |
| Effusion - wrist joint | 202375006 |
| Blood in synovial fluid | 269909003 |
| Effusion of ankle | 202383000 |
| Effusion of talonavicular joint | 202385007 |
| O/E -joint synovial thickening | 164527008 |
| Multiple joint synovial fluid | 167867009 |
| Elbow joint synovial fluid | 167869007 |
| Wrist joint synovial fluid | 167870008 |
| Hand joint synovial fluid | 167871007 |
| Finger joint synovial fluid | 167872000 |
| Effusion - knee joint | 267942009 |
| Synovitis of knee | 239817004 |
| Effusion of 1st MTP joint | 202387004 |
| Effusion of distal radio-ulnar joint | 202374005 |
| Synovial fluid cell count | 60923009 |
| Swollen ankle region | 267039000 |
| Joint fluid haemorrhagic | 269909003 |
| Effusion of first metatarsophalangeal joint | 202387004 |
| Observation of joint swelling | 271771009 |
| Synovial fluid: turbid | 167884003 |
| Synovial fluid specimen | 119332005 |
| Joint fluid hemorrhagic | 269909003 |
| Synovial fluid bloodstained | 269909003 |
| Effusion of joint NOS | 387637008 |
| Synovial fluid exam.- abnormal | 167863008 |
| Effusion - hand joint | 16711001 |
| Synovial fluid cell count | 9.92381E+14 |
| On examination - joint synovial thickening | 164527008 |
| Swollen finger | 299060006 |
| Synovitis of elbow | 239816008 |
| Elbow joint effusion | 202373004 |
| Hip joint effusion | 202379000 |
| Transient synovitis | 202924004 |
| O/E - joint effusion present | 164526004 |
| Toe joint synovial fluid | 167877006 |
| Synovial fluid appearance | 167879009 |
| Synovial fluid: LE cells | 167901007 |
| Synovial fluid exam. - normal | 167862003 |
| Synovial fluid exam.- abnormal | 167863008 |
| Joint effusion of the shoulder region | 40884005 |
| Intermittent hydrarthrosis | 50442003 |
| Cell count of synovial fluid | 60923009 |
| Synovial fluid: yellow | 167881006 |
| Synovial fluid clear | 167880007 |
| Joint effusion of the lower leg | 267942009 |
| Swelling of toe joint | 299577004 |
| Synovitis of left ankle joint | 1.07589E+15 |
| Metatarsophalangeal joint effusion | 298155004 |
| Soft tissue swelling of ankle joint | 299416002 |
| Swelling of right foot | 762915008 |
| Soft tissue swelling of elbow joint | 298872009 |
| Swollen joint count | 3.76481E+14 |
| Swelling of knee joint | 299322007 |
| Foot joint swelling | 299480001 |
| Chronic synovitis | 704174008 |
| Swelling of wrist joint | 298941006 |
| Synovial sample | 309123007 |
| Moderate effusion in knee | 250121005 |
| Finger joint effusion | 298152001 |
| Finger joint - soft tissue swelling | 299126001 |
| Suprapatellar swelling of knee joint | 299327001 |
| Temporomandibular joint swelling | 298367008 |
| Soft tissue swelling of knee joint | 299324008 |
| Finger joint - synovial swelling | 299127005 |
| Foot joint - soft tissue swelling | 299482009 |
| Trace effusion in knee | 250120006 |
| Swelling of left foot | 762916009 |
| Swelling of ankle joint | 299414004 |
| Toe joint - soft tissue swelling | 299579001 |
| Swelling of finger joint | 299123009 |
| Thumb joint - synovial swelling | 299135008 |
| Temporomandibular joint - soft tissue swelling | 298369006 |
| Wrist joint - soft tissue swelling | 298943009 |
| Swollen joint count | 273856009 |
| Swelling of thumb joint | 299132006 |
| Thumb joint - soft tissue swelling | 299134007 |
| Elbow joint - synovial swelling | 298873004 |
| Shoulder joint swelling | 298768000 |
| Elbow joint swelling | 298870001 |
| Painful swelling of joint | 387638003 |

| DESCRIPTION | READ_CODE |
| --- | --- |
| Knee joint effusion | N090611 |
| Effusion of joint | N090.00 |
| O/E - joint swelling | 2H3..00 |
| Joint effusion of unspecified site | N090000 |
| Synovitis of hip | N220S00 |
| Acute joint effusion | N090Y00 |
| Elbow joint effusion | N090211 |
| Ankle swelling symptom | 1832.11 |
| O/E - joint effusion present | 2H32.00 |
| O/E - swelling - joint | 2H3..11 |
| Ankle swelling | 1832 |
| Swelling of joint - effusion | N090.12 |
| Aspiration of fluid from knee joint | 7K6Z800 |
| Swollen knee | 16J4.00 |
| Swollen toe | 16J1.00 |
| Swollen foot | 16J7.00 |
| Swollen hand | 16J6.00 |
| Synovitis of knee | N220z12 |
| Synovial fluid examination | 4B...00 |
| Ankle joint effusion | N090711 |
| Joint effusion of the hand | N090400 |
| Effusion of multiple joints | N090900 |
| Shoulder synovitis | N220z11 |
| Transient synovitis | N220Q00 |
| Synovitis of elbow | N220z13 |
| Swollen joint | 16J3.00 |
| Effusion of knee | N090M00 |
| Effusion of elbow | N090D00 |
| Synovial fluid sample | 4B2..11 |
| Joint effusion of the shoulder region | N090100 |
| O/E - joint swelling NOS | 2H3Z.00 |
| Effusion of shoulder | N090A00 |
| Joint effusion of the ankle and foot | N090700 |
| Synovial fluid exam. - normal | 4B12.00 |
| Synovial fluid exam. - general | 4B1..00 |
| Hip joint effusion | N090511 |
| Synovial fluid exam.- abnormal | 4B13.00 |
| Effusion of ankle | N090P00 |
| Swollen joint count | ZRq1.00 |
| Chronic joint effusion | N090X00 |
| Chronic crepitant synovitis of hand and wrist | N220R00 |
| Joint effusion of the upper arm | N090200 |
| Intermittent joint effusion | N093.13 |
| Effusion of 1st MTP joint | N090T00 |
| Joint effusion of the lower leg | N090600 |
| Hydrarthrosis | N090.11 |
| Synovitis NOS | N220T00 |
| Finger swelling | 1834 |
| O/E -joint synovial thickening | 2H33.00 |
| Effusion of PIP joint of finger | N090H00 |
| Effusion of joint NOS | N090z00 |
| Effusion of DIP joint - finger | N090J00 |
| Elbow joint synovial fluid | 4B23.00 |
| Knee joint synovial fluid | 4B28.00 |
| Synovial fluid - C/S | 4JL7.11 |
| Synovitis of knee | N220V00 |
| Synovial fluid exam. gen. NOS | 4B1Z.00 |
| Synovial fluid for organism | 4JL7.00 |
| Synovial fluid exam. NOS | 4BZ..00 |
| Joint effusion of other specified site | N090800 |
| Effusion of MCP joint | N090G00 |
| Effusion of wrist | N090F00 |
| Intermittent hydrarthrosis | N093.12 |
| Joint effusion of the forearm | N090300 |
| Effusion of hip | N090K00 |
| Synovial fluid: uric acid | 4B74.00 |
| [D]Synovial fluid abnormal | R127.00 |
| Wrist joint effusion | N090311 |
| Finger joint synovial fluid | 4B26.00 |
| Ankle joint synovial fluid | 4B29.00 |
| Effusion of acromioclavicular joint | N090C00 |
| Synovitis of elbow | N220W00 |
| Synovitis of shoulder | N220X00 |
| Intermittent hydrarthrosis | N090W00 |
| Wrist joint synovial fluid | 4B24.00 |
| Effusion of IP joint of toe | N090V00 |
| Synovial fluid sent for exam. | 4B11.00 |
| Effusion of sternoclavicular joint | N090B00 |
| Effusion of sacro-iliac joint | N090L00 |
| Effusion of tibio-fibular joint | N090N00 |
| Joint effusion of the pelvic region and thigh | N090500 |
| Shoulder joint synovial fluid | 4B22.00 |
| Toe joint synovial fluid | 4B2B.00 |
| Synovial fluid: blood stained | 4B34.00 |
| Synovial fluid cell count | 4B6..00 |
| Synovial fluid source | 4B2..00 |
| Effusion of lesser MTP joint | N090U00 |
| Effusion of talonavicular joint | N090R00 |
| Blood in synovial fluid | 4B34.11 |
| Effusion of subtalar joint | N090Q00 |
| Effusion of distal radio-ulnar joint | N090E00 |
| Synovial fluid appearance | 4B3..00 |
| Synovial fluid: joint NOS | 4B2Z.00 |
| Synovial fluid abnorm. content | 4B7..00 |
| Hip joint synovial fluid | 4B27.00 |
| Synovial fluid: no abn.content | 4B71.00 |
| Foot joint synovial fluid | 4B2A.00 |
| Synovial fluid mucin clot test | 4B8..00 |
| Synovial fluid: yellow | 4B32.00 |
| Synovial fluid viscosity | 4B4..00 |
| Synovial fluid viscosity low | 4B43.00 |
| Hand joint synovial fluid | 4B25.00 |
| Synovial fluid: LE cells | 4B72.00 |
| Synovial fluid: turbid | 4B35.00 |
| Effusion of other tarsal joint | N090S00 |

**6. Joint pain**

| DESCRIPTION | SNOMED_CT_CODE |
| --- | --- |
| Knee pain | 30989003 |
| Coxalgia | 49218002 |
| Hip joint pain | 49218002 |
| Arthralgia of the hand | 202472008 |
| Hand joint pain | 202472008 |
| Arthralgia of sternoclavicular joint | 202478007 |
| Arthralgia of acromioclavicular joint | 202479004 |
| Elbow joint pain | 202480001 |
| Wrist joint pain | 202482009 |
| Arthralgia of wrist | 202482009 |
| Ankle joint pain | 202490009 |
| Arthralgia of talonavicular joint | 202493006 |
| Shoulder joint pain | 267949000 |
| Arthralgia of shoulder | 267949000 |
| Arthralgia of the pelvic region and thigh | 267952008 |
| Arthralgia of the lower leg | 267953003 |
| Shoulder joint painful on movement | 298857005 |
| Knee joint pain | 30989003 |
| Arthralgia of hip | 49218002 |
| Pain in joint - coxalgia | 57676002 |
| Ankle/foot joint pain | 267954009 |
| Joint pain NOS | 57676002 |
| Tenderness of head of fibula | 1.56643E+15 |
| Shoulder joint painful on movement | 298857005 |
| Shoulder joint painful on external rotation | 1.89277E+15 |
| Shoulder joint painful on external rotation | 9.21261E+14 |
| Arthralgia of unspecified site | 57676002 |
| Arthralgia of other specified site | 57676002 |
| Arthralgia of other tarsal joint | 57676002 |
| Arthralgia NOS | 57676002 |
| Arthralgia of the forearm | 267950000 |
| Pain in joint - arthralgia | 57676002 |
| Arthralgia of 1st MTP joint | 202495004 |
| Arthralgia of IP joint of toe | 202497007 |
| Arthralgia of lesser MTP joint | 202496003 |
| Arthralgia of MCP joint | 202483004 |
| Arthralgia of sacro-iliac joint | 202487003 |
| Arthralgia of tibio-fibular joint | 202489000 |
| Coxalgia | 49218002 |
| Musculoskeletal pain - joints | 57676002 |
| Gonalgia | 30989003 |
| Sacroiliac joint pain | 202487003 |
| Subtalar joint pain | 202491008 |
| Distal radioulnar joint pain | 202481002 |
| Proximal interphalangeal joint of finger pain | 202484005 |
| Sternoclavicular joint pain | 202478007 |
| Talonavicular joint pain | 202493006 |
| Metacarpophalangeal joint pain | 202483004 |
| Acromioclavicular joint pain | 202479004 |
| On examination - joint movement painful | 164539000 |
| Tibiofibular joint pain | 202489000 |
| Facet joint pain | 247369005 |
| Arthralgia of other specified site | 6.29831E+14 |
| Other joint pain | 6.29831E+14 |
| Arthralgia NOS | 6.87011E+14 |
| Arthralgia of knee | 30989003 |
| Arthralgia of multiple joints | 35678005 |
| Arthralgia of the shoulder region | 267949000 |
| Elbow joint pain | 202480001 |
| Arthralgia of tibiofibular joint | 202489000 |
| Lesser metatarsophalangeal joint pain | 202496003 |
| Painful joint | 57676002 |
| Whipple's arthralgia | 201517005 |
| Pain in joint - coxalgia | 267952008 |
| Arthralgia of the upper arm | 267950000 |
| O/E - joint movement painful | 164539000 |
| Ache in joint | 57676002 |
| Arthralgia of DIP joint of finger | 202485006 |
| Arthralgia of distal radioulnar joint | 202481002 |
| Ankle and/or foot joint pain | 267954009 |
| First metatarsophalangeal joint pain | 202495004 |
| Multiple joint pain | 35678005 |
| Interphalangeal joint of toe pain | 202497007 |
| Painful Shoulder | 267949000 |
| Multiple joint pain | 35678005 |
| Arthralgia of the ankle and foot | 267954009 |
| Arthralgia of elbow | 202480001 |
| Arthralgia of ankle | 202490009 |
| Arthralgia of subtalar joint | 202491008 |
| Hip pain | 49218002 |
| Other joint symptoms of unspecified site | 57676002 |
| Arthralgia of distal radio-ulnar joint | 202481002 |
| Arthralgia of PIP joint of finger | 202484005 |
| Distal interphalangeal joint of finger pain | 202485006 |
| Polyarthralgia | 35678005 |
| Joint pain NOS | 6.87011E+14 |
| Tenderness of knee joint | 299372009 |
| Tenderness of elbow joint | 298928007 |
| Tenderness of sacroiliac joint | 298251000 |
| Tenderness of ankle joint | 299446004 |
| Tenderness of wrist joint | 299017002 |
| Tenderness of subtalar joint | 299553005 |
| Tenderness of thumb joint | 299198008 |
| Tenderness of foot joint | 299512002 |
| Tenderness of joint | 110288007 |

| DESCRIPTION | READ_CODE |
| --- | --- |
| Arthralgia of unspecified site | N094000 |
| Knee joint pain | N094611 |
| Hip joint pain | N094512 |
| Arthralgia of multiple joints | N094900 |
| Elbow joint pain | N094211 |
| Shoulder joint pain | N094111 |
| Wrist joint pain | N094311 |
| Arthralgia of the lower leg | N094600 |
| Pain in joint - arthralgia | N094.00 |
| Arthralgia of wrist | N094F00 |
| Arthralgia of knee | N094M00 |
| Ache in joint | N094.11 |
| Arthralgia of the hand | N094400 |
| Arthralgia of elbow | N094D00 |
| Arthralgia of the forearm | N094300 |
| Arthralgia of DIP joint of finger | N094J00 |
| Musculoskeletal pain - joints | N096.12 |
| Arthralgia of the ankle and foot | N094700 |
| Arthralgia of sternoclavicular joint | N094B00 |
| Arthralgia of sacro-iliac joint | N094L00 |
| Ankle joint pain | N094711 |
| Arthralgia of the pelvic region and thigh | N094500 |
| Arthralgia of the shoulder region | N094100 |
| Arthralgia of the upper arm | N094200 |
| Arthralgia of shoulder | N094A00 |
| Arthralgia of MCP joint | N094G00 |
| Hand joint pain | N094411 |
| Arthralgia of PIP joint of finger | N094H00 |
| Arthralgia NOS | N094z00 |
| Arthralgia of hip | N094K00 |
| Arthralgia of ankle | N094P00 |
| Arthralgia of other specified site | N094800 |
| O/E - joint movement painful | 2H45.00 |
| Arthralgia of acromioclavicular joint | N094C00 |
| Arthralgia of IP joint of toe | N094V00 |
| Arthralgia of 1st MTP joint | N094T00 |
| Arthralgia of talonavicular joint | N094R00 |
| Arthralgia of lesser MTP joint | N094U00 |
| Elbow joint pain | N094D11 |
| Arthralgia of other tarsal joint | N094S00 |
| Arthralgia of tibio-fibular joint | N094N00 |
| Arthralgia of subtalar joint | N094Q00 |
| Arthralgia of distal radio-ulnar joint | N094E00 |
| Shoulder joint painful on movement | 1M02.00 |
| Shoulder joint painful on external rotation | 1M03.00 |

**Co-existing conditions**

1. **Haemorrhoids**

| DESCRIPTION | SNOMED CT CODE |
| --- | --- |
| External haemorrhoids, simple | 195457004 |
| Haemorrhoids in pregnancy and the puerperium - delivered | 200251000 |
| Haemorrhoids in the puerperium | 200255009 |
| Haemorrhoids | 70153002 |
| Piles - haemorrhoids | 70153002 |
| External bleeding haemorrhoids | 26421009 |
| Haemorrhoids - obstetric | 200249004 |
| [RFC] Haemorrhoids | 906251000006106 |
| Second degree internal haemorrhoids | 760871000000103 |
| H/O: haemorrhoids | 1937031000006100 |
| External haemorrhoids with other complications | 23913003 |
| Bleeding haemorrhoids NOS | 51551000 |
| Haemorrhoids in pregnancy and the puerperium unspecified | 200249004 |
| Internal thrombosed haemorrhoids | 52931009 |
| Haemorrhoids in pregnancy and puerperium - deliv + p/n comp | 200252007 |
| Haemorrhoids in pregnancy and puerperium with a/n comp | 200253002 |
| Haemorrhoids NOS | 70153002 |
| Strangulated haemorrhoids NOS | 83256006 |
| Unspecified simple haemorrhoids | 70153002 |
| Strangulated internal haemorrhoids | 23202007 |
| Piles | 70153002 |
| Bleeding external haemorrhoids | 26421009 |
| Strangulated external haemorrhoids | 80829003 |
| Prolapsed internal haemorrhoids | 80426004 |
| Bleeding internal haemorrhoids | 75884004 |
| Thrombosed external haemorrhoids | 26373009 |
| Ulcerated external haemorrhoids | 55168004 |
| Prolapsing internal haemorrhoids requiring manual reduction | 760901000000103 |
| Prolapsed external haemorrhoids | 38059007 |
| External haemorrhoids with other complications | 623581000000109 |
| Internal haemorrhoids | 90458007 |
| Internal haemorrhoids with other complications | 571391000000107 |
| External haemorrhoids with other complications NOS | 23913003 |
| [X]Internal haemorrhoids with other complications | 90458007 |
| [X]Unspecified haemorrhoids with other complications | 70153002 |
| Internal haemorrhoids with other complications | 90458007 |
| Internal haemorrhoids - other | 571391000000107 |
| External haemorrhoids - other | 623581000000109 |
| Internal haemorrhoids with other complications | 90458007 |
| Haemorrhoids with other complications NOS | 70153002 |
| Haemorrhoids - piles | 70153002 |
| External haemorrhoids - simple | 195457004 |
| External thrombosed haemorrhoids | 26373009 |
| Internal haemorrhoids, simple | 195453000 |
| Thrombosed haemorrhoids NOS | 75955007 |
| Haemorrhoids in pregnancy and the puerperium NOS | 200249004 |
| Prolapsed non-reducible internal haemorrhoids | 760911000000101 |
| Internal haemorrhoids | 90458007 |
| Piles - haemorrhoids | 70153002 |
| Fourth degree internal haemorrhoids | 760911000000101 |
| Internal strangulated haemorrhoids | 23202007 |
| Internal bleeding haemorrhoids | 75884004 |
| Haemorrhoids in pregnancy and the puerperium | 200249004 |
| Ulcerated haemorrhoids NOS | 70153002 |
| Other specified haemorrhoids | 70153002 |
| Prolapsed haemorrhoids NOS | 46276006 |
| First degree internal haemorrhoids | 75884004 |
| Third degree internal haemorrhoids | 760901000000103 |
| Ulcerated internal haemorrhoids | 46516001 |
| Haemorrhoids in pregnancy and puerperium with p/n comp | 200254008 |
| Manual reduction of haemorrhoids | 5151004 |
| Prolapsing internal haemorrhoids with spontaneous retraction | 760871000000103 |
| Internal haemorrhoids with other complications NOS | 90458007 |
| Haemorrhoids with other complications NOS | 70153002 |
| [X]External haemorrhoids with other complications | 23913003 |
| Internal haemorrhoids grade II | 721704005 |
| Internal haemorrhoids grade I | 721703004 |
| Ulcerated haemorrhoids | 5201002 |
| Internal haemorrhoids grade III | 721705006 |
| External haemorrhoids without complication | 38996000 |
| Internal haemorrhoids grade IV | 721706007 |

| DESCRIPTION | READ CODE |
| --- | --- |
| Haemorrhoids | G84..00 |
| External haemorrhoids, simple | G843.00 |
| Haemorrhoids NOS | G84z.00 |
| Bleeding haemorrhoids NOS | G848000 |
| Piles - haemorrhoids | G84..11 |
| Internal strangulated haemorrhoids | G842200 |
| Internal haemorrhoids, simple | G840.00 |
| Haemorrhoids in pregnancy and the puerperium | L416.00 |
| Internal bleeding haemorrhoids | G842000 |
| External bleeding haemorrhoids | G845000 |
| Prolapsed haemorrhoids NOS | G848100 |
| External haemorrhoids with other complications | G845.00 |
| Unspecified simple haemorrhoids | G846.00 |
| Internal thrombosed haemorrhoids | G841.00 |
| Internal haemorrhoids with other complications | G842.00 |
| Strangulated haemorrhoids NOS | G848200 |
| External haemorrhoids with other complications NOS | G845z00 |
| Haemorrhoids with other complications NOS | G848.00 |
| Haemorrhoids with other complications NOS | G848z00 |
| Ulcerated haemorrhoids NOS | G848300 |
| [X]External haemorrhoids with other complications | Gyu8400 |
| Internal haemorrhoids with other complications NOS | G842z00 |
| Haemorrhoids in pregnancy and the puerperium unspecified | L416000 |
| Haemorrhoids in the puerperium | L416500 |
| Haemorrhoids in pregnancy and the puerperium - delivered | L416100 |
| Haemorrhoids in pregnancy and puerperium - deliv + p/n comp | L416200 |
| Haemorrhoids in pregnancy and the puerperium NOS | L416z00 |
| [X]Internal haemorrhoids with other complications | Gyu8300 |
| First degree internal haemorrhoids | G842400 |
| Second degree internal haemorrhoids | G842500 |
| Third degree internal haemorrhoids | G842600 |
| Haemorrhoids in pregnancy and puerperium with a/n comp | L416300 |
| Fourth degree internal haemorrhoids | G842700 |

1. **Irritable bowel syndrome**

| DESCRIPTION | SNOMED CT CODE |
| --- | --- |
| Irritable bowel syndrome | 10743008 |
| Constipation - functional | 197118003 |
| Irritable bowel syndrome with diarrhoea | 197125005 |
| History of irritable bowel syndrome | 70871000119100 |
| Irritable bowel - IBS | 10743008 |
| Irritable colon - Irritable bowel syndrome | 10743008 |
| Irritable bowel | 10743008 |
| Irritable bowel syndrome characterized by constipation | 440630006 |
| Irritable bowel syndrome with diarrhea | 197125005 |
| Irritable bowel syndrome characterized by alternating bowel habit | 440544005 |
| Constipation predominant irritable bowel syndrome | 440630006 |
| IBS - Irritable bowel syndrome | 10743008 |
| Management of irritable bowel syndrome | 817511000000108 |
| IC - Irritable colon | 10743008 |
| Irritable bowel syndrome characterised by constipation | 440630006 |
| [X]Psychogenic IBS | 231517009 |
| IBS characterised by alternating bowel habit | 440544005 |
| Constipation alternates with diarrhoea | 249517009 |

| **DESCRIPTION** | **READ CODE** |
| --- | --- |
| Irritable bowel syndrome | J521.11 |
| Irritable colon - Irritable bowel syndrome | J521.00 |
| [X]Psychogenic IBS | Eu45324 |
| Irritable bowel syndrome with diarrhoea | J521000 |
| History of irritable bowel syndrome | 14CF.00 |
| Management of irritable bowel syndrome | 8Cm..00 |
| Irritable bowel syndrome characterised by constipation | J521100 |
| IBS characterised by alternating bowel habit | J521200 |

| **DESCRIPTION** | **SNOMED CT CODE** |
| --- | --- |
| Dental phobia | 38617005 |
| Simple phobia | 54587008 |
| Acrophobia | 58963008 |
| H/O: anxiety state | 161470009 |
| Recurrent anxiety | 191709001 |
| Social phobia, fear of eating in public | 191724005 |
| Social phobia, fear of public speaking | 191725006 |
| Anxiety management training | 228560001 |
| Flying phobia | 247854002 |
| Generalised anxiety disorder | 21897009 |
| Cancer phobia | 34563004 |
| Panic disorder | 371631005 |
| Animal phobia | 54307006 |
| H/O: agoraphobia | 414371008 |
| Referral for guided self-help for anxiety | 1.99101E+14 |
| Anxious | 48694002 |
| Phonophobia | 8.51141E+14 |
| School phobia | 8.51351E+14 |
| [RFC] Anxiety management | 9.09691E+14 |
| Needle phobia | 54587008 |
| ** The treatment of anxiety disorders | 9.72931E+14 |
| Panic disorder without agoraphobia | 56576003 |
| Referral for guided self-help for anxiety declined | 9.33461E+14 |
| Referral for psychological management of anxiety | 1.03745E+15 |
| Anxiety state unspecified | 198288003 |
| Anxiety state NOS | 198288003 |
| Agoraphobia without mention of panic attacks | 61569007 |
| [X]Phobic anxiety disorders | 386810004 |
| [X]Phobic anxiety disorder, unspecified | 386810004 |
| [X]Other anxiety disorders | 197480006 |
| [X]Separation anxiety disorder of childhood | 11806006 |
| [X]Agoraphobia | 70691001 |
| [X]Agoraphobia without history of panic disorder | 70691001 |
| [X]Animal phobias | 54307006 |
| [X]Anxiety hysteria | 197480006 |
| [X]Anxiety neurosis | 207363009 |
| [X]Childhood overanxious disorder | 13438001 |
| [X]Dream anxiety disorder | 419145002 |
| [X]Generalized anxiety disorder | 21897009 |
| [X]Mild anxiety depression | 231504006 |
| [X]Mixed anxiety and depressive disorder | 231504006 |
| [X]Nosophobia | 18193002 |
| [X]Organic anxiety disorder | 17496003 |
| [X]Panic state | 371631005 |
| [X]Social anxiety disorder of childhood | 64165008 |
| [X]Social phobias | 25501002 |
| Anxiety with depression | 231504006 |
| Disturbance anxiety and fearfulness childhood/adolescent NOS | 192108001 |
| [X]Needle phobia | 231501003 |
| [X]Other phobic anxiety disorders | 386810004 |
| [X]Persistant anxiety depression | 231504006 |
| [X]Simple phobia | 386810004 |
| Anxiety states | 197480006 |
| Management of anxiety | 710060004 |
| Adjustment disorder with anxiety | 47372000 |
| AMT - Anxiety management training | 228560001 |
| History of agoraphobia | 414371008 |
| Phobia of going out | 70691001 |
| History of anxiety state | 161470009 |
| Adjustment disorder with anxious mood | 47372000 |
| Episodic paroxysmal anxiety disorder | 371631005 |
| [X]Phobia NOS | 386810004 |
| Social anxiety disorder | 25501002 |
| Feeling anxious | 48694002 |
| Anxious cognitions | 79015004 |
| Anxiety counseling | 313087008 |
| GAD - Generalised anxiety disorder | 21897009 |
| Anxiety | 48694002 |
| Isolated phobia | 54587008 |
| Counseling for anxiety | 313087008 |
| Generalized anxiety disorder | 21897009 |
| GAD - Generalized anxiety disorder | 21897009 |
| Agoraphobia | 191723004 |
| Needle phobia | 5.63201E+14 |
| Agoraphobia without mention of panic attacks | 191723004 |
| Referral for psychological management of anxiety declined | 1037471000000100 |
| Feeling anxious | 1818111000006100 |
| Agoraphobia | 191722009 |
| O/E - anxious | 162723006 |
| [X]Anxiety disorder, unspecified | 197480006 |
| [X]Acrophobia | 54587008 |
| [X]Anxiety NOS | 197480006 |
| [X]Phobic anxiety disorder of childhood | 192611004 |
| Disturbance of anxiety and fearfulness childhood/adolescent | 192108001 |
| Counselling for anxiety | 313087008 |
| On examination - anxious | 162723006 |
| Phobia unspecified | 386808001 |
| [X]Panic disorder [episodic paroxysmal anxiety] | 371631005 |
| Breathlessness causing anxiety | 1861181000006100 |
| Phobic anxiety | 853241000006103 |
| Chronic anxiety | 191708009 |
| Social phobia, fear of public washing | 191726007 |
| Organic anxiety disorder | 17496003 |
| [X]Anthropophobia | 25501002 |
| [X]Anxious [avoidant] personality disorder | 231528008 |
| [X]Panic disorder with agoraphobia | 191722009 |
| Overanxious disorder of childhood | 13438001 |
| Zoophobia | 54307006 |
| [X]Other mixed anxiety disorders | 231504006 |
| Anxiety resolved | 1037391000000100 |
| Separation anxiety disorder | 11806006 |
| Agoraphobia with panic attacks | 191722009 |
| Claustrophobia | 19887002 |
| [X]Other specified anxiety disorders | 197480006 |
| Phobic anxiety | 386810004 |
| Childhood phobic anxiety disorder | 192611004 |
| [X]Anxiety state | 198288003 |
| [X]Claustrophobia | 54587008 |
| [X]Phobia NOS | 386810004 |
| [X]Specific (isolated) phobias | 54587008 |
| Anxiety counselling | 313087008 |
| Anxiety depression | 231504006 |
| Cyesiophobia | 191733007 |
| [X]Organic anxiety disorder | 17496003 |
| Other phobias | 563201000000101 |
| Anxiety state | 198288003 |

| **DESCRIPTION** | **READ CODE** |
| --- | --- |
| Anxiety states | E200.00 |
| Anxiety with depression | E200300 |
| [X]Anxiety neurosis | Eu41111 |
| Cancer phobia | E202B00 |
| Claustrophobia | E202800 |
| Chronic anxiety | E200400 |
| Phobia unspecified | E202000 |
| Dental phobia | E202C00 |
| [X]Agoraphobia | Eu40000 |
| Agoraphobia with panic attacks | E202100 |
| H/O: anxiety state | 1466 |
| Panic disorder | E200100 |
| [X]Panic state | Eu41012 |
| Anxiety state NOS | E200z00 |
| Recurrent anxiety | E200500 |
| Generalised anxiety disorder | E200200 |
| [X]Other anxiety disorders | Eu41.00 |
| Separation anxiety disorder | E292000 |
| Anxiety state unspecified | E200000 |
| [X]Phobia NOS | Eu40z11 |
| [X]Mild anxiety depression | Eu41211 |
| Anxiety counselling | Z4L1.00 |
| [X]Panic disorder [episodic paroxysmal anxiety] | Eu41000 |
| [X]Anxious [avoidant] personality disorder | Eu60600 |
| Anxiety management training | 8G94.00 |
| [X]Phobic anxiety disorders | Eu40.00 |
| [X]Specific (isolated) phobias | Eu40200 |
| Phobic anxiety | E202.12 |
| [X]Generalized anxiety disorder | Eu41100 |
| [X]Claustrophobia | Eu40213 |
| [X]Social phobias | Eu40100 |
| H/O: agoraphobia | 146G.00 |
| [X]Mixed anxiety and depressive disorder | Eu41200 |
| [X]Needle phobia | Eu40300 |
| [X]Simple phobia | Eu40214 |
| Agoraphobia without mention of panic attacks | E202200 |
| O/E - anxious | 2258 |
| [X]Panic disorder with agoraphobia | Eu40012 |
| [X]Persistant anxiety depression | Eu34114 |
| Social phobia, fear of eating in public | E202300 |
| [X]Agoraphobia without history of panic disorder | Eu40011 |
| [X]Dream anxiety disorder | Eu51511 |
| [X]Separation anxiety disorder of childhood | Eu93000 |
| [X]Animal phobias | Eu40212 |
| Social phobia, fear of public washing | E202500 |
| [X]Organic anxiety disorder | Eu05400 |
| Flying phobia | E28z.12 |
| [X]Anxiety disorder, unspecified | Eu41z00 |
| [X]Other specified anxiety disorders | Eu41y00 |
| [X]Phobic anxiety disorder of childhood | Eu93100 |
| [X]Anxiety NOS | Eu41z11 |
| Phobia counselling | Z481.00 |
| Reducing anxiety | Z4I7211 |
| [X]Other phobic anxiety disorders | Eu40y00 |
| Acrophobia | E202600 |
| [X]Anxiety hysteria | Eu41y11 |
| Referral for guided self-help for anxiety | 8HHp.00 |
| Animal phobia | E202700 |
| [X]Social anxiety disorder of childhood | Eu93200 |
| Disturbance of anxiety and fearfulness childhood/adolescent | E2D0.00 |
| Social phobia, fear of public speaking | E202400 |
| [X]Phobic anxiety disorder, unspecified | Eu40z00 |
| Disturbance anxiety and fearfulness childhood/adolescent NOS | E2D0z00 |
| [X]Other mixed anxiety disorders | Eu41300 |
| [X]Anxiety state | Eu41113 |
| [X]Childhood overanxious disorder | Eu93y12 |
| [X]Nosophobia | Eu45215 |
| [X]Acrophobia | Eu40211 |
| Anxious | 1B13.12 |
| Desensitisation - phobia | Z522400 |
| Referral for guided self-help for anxiety declined | 8IH5300 |

1. **Depression**

| DESCRIPTION | SNOMED CT CODE |
| --- | --- |
| Agitated depression | 83458005 |
| Senile dementia with depression | 191459006 |
| Arteriosclerotic dementia with depression | 191466007 |
| Single major depressive episode, severe, with psychosis | 191604000 |
| Recurrent major depressive episodes, mild | 191610000 |
| Recurrent major depressive episodes, moderate | 191611001 |
| Recurrent major depressive episodes, severe, with psychosis | 191613003 |
| Reactive depressive psychosis | 191676002 |
| Psychotic reactive depression | 191676002 |
| Postviral depression | 192079006 |
| Endogenous depression first episode | 231499006 |
| Masked depression | 231500002 |
| Seasonal affective disorder | 247803002 |
| Endogenous depression - recurrent | 274948002 |
| Depression management programme | 401174001 |
| On depression register | 413169006 |
| Patient given advice about management of depression | 415044007 |
| Depression interim review | 413973005 |
| Depression medication review | 413974004 |
| Depression annual review | 413972000 |
| [X]Mild depression | 310495003 |
| Referral for guided self-help for depression | 199111000000100 |
| Depression monitoring first letter | 717211000000107 |
| Depression monitoring third letter | 716961000000102 |
| On full dose long term treatment for depression | 361761000000106 |
| Post natal depression | 853871000006107 |
| Reactive (neurotic) depression | 87414006 |
| [RFC] Postnatal depression | 908731000006105 |
| Antenatal depression | 1771531000006100 |
| Unspecified dementia, other symptoms, predominantly depressive | 1972071000006100 |
| Organic depressive disorder | 1972111000006100 |
| Dementia in Alzheimer's dis, atypical or mixed type, other symptoms, predominantly depressive | 1972311000006100 |
| Dementia in Alzheimer's disease, unspecified, other symptoms, predominantly depressive | 1972451000006100 |
| Vascular dementia of acute onset, other symptoms, predominantly depressive | 1972541000006100 |
| Mixed cortical and subcortical vascular dementia, other symptoms, predominantly depressive | 1972911000006100 |
| Other vascular dementia, other symptoms, predominantly depressive | 1973381000006100 |
| Post-schizophrenic depression, continuous | 1975191000006100 |
| Post-schizophrenic depression, episodic with progressive deficit | 1975211000006100 |
| Post-schizophrenic depression, episodic remittent | 1975261000006100 |
| Post-schizophrenic depression, course uncertain, period of observation too short | 1975321000006100 |
| Mild depressive episode, without somatic syndrome | 1975981000006100 |
| Moderate depressive episode, without somatic syndrome | 1976021000006100 |
| Recurrent depressive disorder, current episode mild, with somatic syndrome | 1976231000006100 |
| Recurrent depressive disorder, current episode moderate, with somatic syndrome | 1976271000006100 |
| Mixed anxiety and depressive reaction | 1976491000006100 |
| Post-schizophrenic depression, other | 1976921000006100 |
| Maternal postnatal depression | 1038261000000100 |
| Signposting to depression self-help group | 1057351000000100 |
| Senile dementia with depressive or paranoid features NOS | 191457008 |
| Recurrent major depressive episodes, unspecified | 268621008 |
| Brief depressive reaction NOS | 192046006 |
| Depressive disorder NEC | 35489007 |
| [X]Other recurrent depressive disorders | 191616006 |
| [X]Other depressive episodes | 35489007 |
| [X]Depressive episode, unspecified | 35489007 |
| [X]Recurrent depressive disorder | 191616006 |
| [X]Recurrent depressive disorder, unspecified | 191616006 |
| Prolonged depressive reaction | 192049004 |
| [X] Reactive depression NOS | 87414006 |
| [X]Depressive conduct disorder | 231542000 |
| [X]Depressive episode | 35489007 |
| [X]Depressive neurosis | 78667006 |
| [X]Dysthymia | 78667006 |
| [X]Mild anxiety depression | 231504006 |
| [X]Mixed anxiety and depressive disorder | 231504006 |
| [X]Monopolar depression NOS | 35489007 |
| [X]Neurotic depression | 78667006 |
| Depression monitoring telephone invite | 716421000000103 |
| [X]Post-schizophrenic depression | 231485007 |
| [X]Postnatal depression NOS | 58703003 |
| [X]Postpartum depression NOS | 58703003 |
| [X]Recurr severe episodes/psychogenic depressive psychosis | 191613003 |
| [X]Recurrent brief depressive episodes | 40568001 |
| [X]Recurrent depress disorder cur epi severe with psyc symp | 28475009 |
| [X]Recurrent episodes of depressive reaction | 191616006 |
| [X]Recurrent episodes of psychogenic depression | 191616006 |
| [X]Recurrent episodes of reactive depression | 191616006 |
| [X]Schizoaffective disorder, depressive type | 84760002 |
| [X]Schizoaffective psychosis, depressive type | 84760002 |
| [X]Seasonal depressive disorder | 247803002 |
| [X]Single episode of masked depression NOS | 231500002 |
| [X]Single episode of reactive depression | 87414006 |
| Anxiety with depression | 231504006 |
| Brief depressive reaction | 192046006 |
| Depressive psychoses | 35489007 |
| [X]Major depression, severe with psychotic symptoms | 73867007 |
| [X]Major depression, severe without psychotic symptoms | 75084000 |
| [X]Antenatal depression | 790961000000101 |
| Recurrent major depressive episode NOS | 268621008 |
| [X]Recurrent depressive disorder, current episode mild | 310495003 |
| [X]Recurrent depressive disorder, current episode moderate | 310496002 |
| [D]Postoperative depression | 82218004 |
| Recurrent major depressive episodes, severe, no psychosis | 764611000000100 |
| [X]Persistant anxiety depression | 231504006 |
| [X]Prolonged single episode of reactive depression | 87414006 |
| [X]Single episode of psychogenic depression | 87414006 |
| [X]Single episode of reactive depressive psychosis | 191676002 |
| [X]Mild depressive episode | 310495003 |
| [X]Moderate depressive episode | 310496002 |
| Single major depressive episode | 36923009 |
| [X]Severe depressive episode without psychotic symptoms | 310497006 |
| [X]Single episode major depression w'out psychotic symptoms | 310497006 |
| [X]Recurrent severe episodes/reactive depressive psychosis | 1086471000000100 |
| Single major depressive episode, severe, without psychosis | 251000119105 |
| Mild depression | 310495003 |
| Major depressive disorder, single episode | 36923009 |
| History of depression | 161469008 |
| Single major depressive episode, moderate | 15639000 |
| Counseling for postnatal depression | 395072006 |
| [X]Recurrent severe episodes/reactive depressive psychosis | 191613003 |
| Psychotic depression | 73867007 |
| Moderate depression | 310496002 |
| Counselling for postnatal depression | 395072006 |
| Depressive conduct disorder | 231542000 |
| Postoperative depression | 82218004 |
| Depression management program | 401174001 |
| Recurrent reactive depressive episodes, severe, with psychosis | 1086471000000100 |
| Severe recurrent major depression with psychotic features | 28475009 |
| Schizophreniform psychosis, depressive type | 84760002 |
| Depressive illness | 35489007 |
| Recurrent brief depressive disorder | 40568001 |
| Post-schizophrenic depression | 231485007 |
| History of depressive disorder | 161469008 |
| Depressive disorder NEC | 609311000000100 |
| Depression | 609311000000100 |
| Depression NOS | 609311000000100 |
| Moderate depression | 465441000000108 |
| Severe depression | 397701000000102 |
| Depression care management | 784051000000106 |
| [X]Mild depressive episode | 430421000000104 |
| Referral for guided self-help for depression declined | 933441000000101 |
| Dementia in Alzheimer's disease with early onset, other symptoms, predominantly depressive | 1972131000006100 |
| Multi-infarct dementia, other symptoms, predominantly depressive | 1972661000006100 |
| Recurrent depressive disorder, current episode moderate, without somatic syndrome | 1976251000006100 |
| Depression resolved | 196381000000100 |
| Atypical depressive disorder | 191659001 |
| Depression - enhanced service completed | 166481000000107 |
| [X]Depression NOS | 35489007 |
| [X]Depressive disorder NOS | 35489007 |
| [X]Recurr severe episodes/major depression+psychotic symptom | 28475009 |
| [X]SAD - Seasonal affective disorder | 247803002 |
| Neurotic depression reactive type | 87414006 |
| On full dose long term treatment depression - enh serv admin | 361761000000106 |
| [X]Severe depressive episode with psychotic symptoms | 191604000 |
| [X]Endogenous depression with psychotic symptoms | 73867007 |
| [X]Major depression, recurrent without psychotic symptoms | 268621008 |
| [X]Recurr depress disorder cur epi severe without psyc sympt | 310497006 |
| [X]Single episode of major depression and psychotic symptoms | 191604000 |
| [X]Single episode of psychogenic depressive psychosis | 191676002 |
| Severe depression | 310497006 |
| Depression care management | 784051000000106 |
| [X]Severe depressive episode without psychotic symptoms | 397701000000102 |
| QOF (Quality and Outcomes Framework) depression quality indicator-related care invitation | 1110911000000100 |
| Referral for depression self-help video | 923921000000104 |
| Dementia in Alzheimer's disease with late onset, other symptoms, predominantly depressive | 1972201000006100 |
| Subcortical vascular dementia, other symptoms, predominantly depressive | 1972771000006100 |
| Mild depressive episode, with somatic syndrome | 1975991000006100 |
| Moderate depressive episode, with somatic syndrome | 1976051000006100 |
| Recurrent depressive disorder, current episode mild, without somatic syndrome | 1976211000006100 |
| Depression confirmed | 1823881000006100 |
| [RFC] Depression | 909681000006106 |
| Postnatal depression discussed | 939961000006100 |
| Depression monitoring administration | 713831000000108 |
| Depression monitoring second letter | 716681000000100 |
| Endogenous depression | 300706003 |
| Drug-induced depressive state | 191495003 |
| Recurrent depression | 191616006 |
| Postnatal depression | 58703003 |
| Depression monitoring verbal invite | 717261000000109 |
| [X]Recurrent severe episodes of psychotic depression | 191613003 |
| Agitated depression | 83458005 |
| Endogenous depression first episode | 231499006 |
| [X]Major depression, moderately severe | 832007 |
| [X]Major depression, mild | 87512008 |
| [X]Endogenous depression without psychotic symptoms | 300706003 |
| [X]Vital depression, recurrent without psychotic symptoms | 310497006 |
| Single major depressive episode, mild | 79298009 |
| Single major depressive episode, moderate | 15639000 |
| Single major depressive episode, unspecified | 36923009 |
| [X]Single episode vital depression w'out psychotic symptoms | 310497006 |
| Severe recurrent major depression without psychotic features | 36474008 |
| Drug-induced depression | 191495003 |
| Severe major depression, single episode | 251000119105 |
| Vascular dementia, unspecified, other symptoms, predominantly depressive | 1973551000006100 |
| Post-schizophrenic depression, episodic with stable deficit | 1975231000006100 |
| Other recurrent mood affective disorders, recurrent brief depressive disorder | 1976411000006100 |
| Moderate major depression, single episode | 15639000 |
| Puerperal depression | 58703003 |
| Postnatal depression counselling | 395072006 |
| Presenile dementia with depression | 191455000 |
| Senile dementia with depressive or paranoid features | 191457008 |
| Chronic depression | 192080009 |
| H/O: depression | 161469008 |
| Recurrent major depressive episode | 268621008 |
| Depression - enhanced services administration | 166291000000108 |
| Major depression, single episode | 36923009 |
| [X]Schizophreniform psychosis, depressive type | 84760002 |
| [X]Single episode of psychotic depression | 191604000 |
| [X]Atypical depression | 191659001 |
| [X]Single episode of depressive reaction | 87414006 |
| Single major depressive episode NOS | 36923009 |
| [X]Single episode agitated depressn w'out psychotic symptoms | 310497006 |
| Postnatal depression counseling | 395072006 |
| Anxiety depression | 231504006 |
| Mild major depression, single episode | 79298009 |
| [X]Moderate depressive episode | 465441000000108 |
| Mild depression | 430421000000104 |

| **DESCRIPTION** | **READ CODE** |
| --- | --- |
| Depressive disorder NEC | E2B..00 |
| [X]Depression NOS | Eu32z11 |
| Endogenous depression | E112.14 |
| Anxiety with depression | E200300 |
| Agitated depression | E135.00 |
| Neurotic depression reactive type | E204.00 |
| Brief depressive reaction | E290.00 |
| Depressive psychoses | E11..12 |
| Postnatal depression | E204.11 |
| H/O: depression | 1465 |
| Puerperal depression | 62T1.00 |
| [X]Depressive episode, unspecified | Eu32z00 |
| Postviral depression | E2B0.00 |
| [X]Depressive disorder NOS | Eu32z12 |
| [X]Recurrent depressive disorder | Eu33.00 |
| Chronic depression | E2B1.00 |
| [X]Depressive episode | Eu32.00 |
| [X]Postpartum depression NOS | Eu53012 |
| Agitated depression | E112.11 |
| [X] Reactive depression NOS | Eu32z14 |
| Recurrent depression | E113700 |
| Endogenous depression first episode | E112.12 |
| [X]Other depressive episodes | Eu32y00 |
| Endogenous depression - recurrent | E113.11 |
| Endogenous depression first episode | E112.13 |
| Single major depressive episode NOS | E112z00 |
| [X]Single episode of reactive depression | Eu32.13 |
| [X]Neurotic depression | Eu34113 |
| [X]Mild anxiety depression | Eu41211 |
| [X]Dysthymia | Eu34100 |
| Reactive depressive psychosis | E130.00 |
| [X]Depressive neurosis | Eu34111 |
| [X]SAD - Seasonal affective disorder | Eu33.15 |
| [X]Recurrent episodes of depressive reaction | Eu33.11 |
| [X]Recurrent episodes of reactive depression | Eu33.13 |
| [X]Single episode of depressive reaction | Eu32.11 |
| Masked depression | E11z200 |
| [X]Moderate depressive episode | Eu32100 |
| [X]Severe depressive episode without psychotic symptoms | Eu32200 |
| Single major depressive episode | E112.00 |
| [X]Mild depression | Eu32400 |
| [X]Atypical depression | Eu32y11 |
| Seasonal affective disorder | E118.00 |
| [X]Schizoaffective disorder, depressive type | Eu25100 |
| [X]Major depression, recurrent without psychotic symptoms | Eu33212 |
| [X]Endogenous depression without psychotic symptoms | Eu33211 |
| [X]Mild depressive episode | Eu32000 |
| [X]Mixed anxiety and depressive disorder | Eu41200 |
| [X]Severe depressive episode with psychotic symptoms | Eu32300 |
| Depression medication review | 9H91.00 |
| Depression annual review | 9H90.00 |
| [X]Postnatal depression NOS | Eu53011 |
| Recurrent major depressive episodes, moderate | E113200 |
| Recurrent major depressive episode | E113.00 |
| Single major depressive episode, moderate | E112200 |
| Single major depressive episode, severe, without psychosis | E112300 |
| [X]Persistant anxiety depression | Eu34114 |
| Single major depressive episode, mild | E112100 |
| Prolonged depressive reaction | E291.00 |
| [X]Recurrent severe episodes of psychotic depression | Eu33315 |
| Psychotic reactive depression | E130.11 |
| [X]Single episode of psychogenic depression | Eu32.12 |
| Postnatal depression counselling | 6G00.00 |
| [X]Recurrent brief depressive episodes | Eu3y111 |
| Depression resolved | 212S.00 |
| [X]Recurrent episodes of psychogenic depression | Eu33.12 |
| [X]Post-schizophrenic depression | Eu20400 |
| Senile dementia with depression | E002100 |
| [X]Single episode major depression w'out psychotic symptoms | Eu32212 |
| [X]Endogenous depression with psychotic symptoms | Eu33311 |
| [X]Single episode of psychotic depression | Eu32313 |
| [X]Single episode of major depression and psychotic symptoms | Eu32311 |
| Recurrent major depressive episodes, severe, with psychosis | E113400 |
| Recurrent major depressive episode NOS | E113z00 |
| Recurrent major depressive episodes, severe, no psychosis | E113300 |
| Atypical depressive disorder | E11y200 |
| Presenile dementia with depression | E001300 |
| [X]Prolonged single episode of reactive depression | Eu32z13 |
| [X]Seasonal depressive disorder | Eu33.14 |
| [X]Single episode of reactive depressive psychosis | Eu32314 |
| Recurrent major depressive episodes, mild | E113100 |
| [X]Recurrent depressive disorder, current episode moderate | Eu33100 |
| [D]Postoperative depression | R007z13 |
| [X]Recurrent depressive disorder, current episode mild | Eu33000 |
| Depression interim review | 9H92.00 |
| Patient given advice about management of depression | 8CAa.00 |
| Depression - enhanced services administration | 9k4..00 |
| [X]Recurr severe episodes/psychogenic depressive psychosis | Eu33314 |
| Single major depressive episode, severe, with psychosis | E112400 |
| Referral for guided self-help for depression | 8HHq.00 |
| [X]Depressive conduct disorder | Eu92000 |
| [X]Recurr severe episodes/major depression+psychotic symptom | Eu33313 |
| [X]Recurr depress disorder cur epi severe without psyc sympt | Eu33200 |
| Single major depressive episode, unspecified | E112000 |
| [X]Schizoaffective psychosis, depressive type | Eu25111 |
| Recurrent major depressive episodes, unspecified | E113000 |
| Brief depressive reaction NOS | E290z00 |
| [X]Monopolar depression NOS | Eu33z11 |
| [X]Recurrent severe episodes/reactive depressive psychosis | Eu33316 |
| [X]Schizophreniform psychosis, depressive type | Eu25112 |
| Senile dementia with depressive or paranoid features NOS | E002z00 |
| [X]Single episode agitated depressn w'out psychotic symptoms | Eu32211 |
| On depression register | 9HA0.00 |
| Arteriosclerotic dementia with depression | E004300 |
| [X]Recurrent depressive disorder, unspecified | Eu33z00 |
| Senile dementia with depressive or paranoid features | E002.00 |
| Depression management programme | 8BK0.00 |
| Drug-induced depressive state | E02y300 |
| [X]Recurrent depress disorder cur epi severe with psyc symp | Eu33300 |
| [X]Other recurrent depressive disorders | Eu33y00 |
| Depression monitoring administration | 9Ov..00 |
| [X]Single episode of psychogenic depressive psychosis | Eu32312 |
| [X]Single episode of masked depression NOS | Eu32y12 |
| [X]Single episode vital depression w'out psychotic symptoms | Eu32213 |
| Depression - enhanced service completed | 9k40.00 |
| Depression monitoring first letter | 9Ov0.00 |
| Depression monitoring second letter | 9Ov1.00 |
| [X]Vital depression, recurrent without psychotic symptoms | Eu33214 |
| Depression monitoring telephone invite | 9Ov4.00 |
| Depression monitoring verbal invite | 9Ov3.00 |
| Depression monitoring third letter | 9Ov2.00 |
| On full dose long term treatment depression - enh serv admin | 9kQ..00 |
| [X]Major depression, moderately severe | Eu32600 |
| [X]Major depression, mild | Eu32500 |
| [X]Major depression, severe without psychotic symptoms | Eu32700 |
| [X]Major depression, severe with psychotic symptoms | Eu32800 |
| [X]Single major depr ep, severe with psych, psych in remiss | Eu32900 |
| [X]Recurr major depr ep, severe with psych, psych in remiss | Eu32A00 |
| [X]Antenatal depression | Eu32B00 |
| Referral for guided self-help for depression declined | 8IH5200 |

1. **Family History of IBD**

| **DESCRIPTION** | **SNOMED CT CODE** |
| --- | --- |
| FH: Crohn's disease | 160386006 |
| FH: Ulcerative colitis | 275129008 |
| Family history: Crohn's disease | 160386006 |
| Family history of Crohn's disease | 160386006 |
| Family history: Ulcerative colitis | 275129008 |
| Family history: Crohn disease | 160386006 |
| Family history of ulcerative colitis | 275129008 |

| **DESCRIPTION** | **READ CODE** |
| --- | --- |
| FH: Ulcerative colitis | 12E2.11 |
| FH: Crohn's disease | 12E5.00 |

1. **Appendicectomy**

| **DESCRIPTION** | **SNOMED CT CODE** |
| --- | --- |
| Endoscopic appendicectomy NEC | 6025007 |
| Appendicectomy NEC | 80146002 |
| Appendicectomy with drainage | 49438003 |
| Emergency appendicectomy | 174036004 |
| History of appendicectomy | 428251008 |
| Incidental appendicectomy | 82730006 |
| Inversion appendicectomy | 235314005 |
| Prophylactic appendicectomy NEC | 80146002 |
| Appendicectomy | 661131000000108 |
| Interval appendicectomy | 174045003 |
| Planned delayed appendicectomy NEC | 80146002 |
| Endoscopic emergency appendicectomy | 174041007 |
| Endoscopic appendicectomy | 6025007 |
| Emergency appendicectomy NEC | 174036004 |
| Laparoscopic emergency appendicectomy | 174041007 |
| Secondary appendicectomy | 82730006 |
| Laparoscopic interval appendicectomy | 307581005 |
| Non emergency appendicectomy | 235313004 |
| Appendicectomy and drainage | 49438003 |

| **DESCRIPTION** | **READ CODE** |
| --- | --- |
| Appendicectomy NEC | 7701z11 |
| Non emergency appendicectomy | 7701.11 |
| Emergency appendicectomy | 7700.11 |
| Incidental appendicectomy | 7701200 |
| Planned delayed appendicectomy NEC | 7701300 |
| Emergency appendicectomy NEC | 7700300 |
| Endoscopic emergency appendicectomy | 7700400 |
| Interval appendicectomy | 7701000 |
| Appendicostomy | 7702200 |
| Prophylactic appendicectomy NEC | 7701100 |
| Endoscopic appendicectomy NEC | 7701400 |
| Appendicocaecostomy | 771A500 |
| Weir appendicostomy | 771Ay11 |
| Non emergency appendicectomy | 7701.11 |
| Prophylactic appendicectomy NEC | 7701100 |
| Appendicectomy NEC | 7701z11 |
| Planned delayed appendicectomy NEC | 7701300 |
| Emergency appendicectomy NEC | 7700300 |
| Interval appendicectomy | 7701000 |
| Endoscopic appendicectomy NEC | 7701400 |
| Incidental appendicectomy | 7701200 |
| Endoscopic emergency appendicectomy | 7700400 |
| Emergency appendicectomy | 7700.11 |

**AHD codes for blood and stool tests**

1. **Haemoglobin**

| 813551000006113 | Haemoglobin_estimation |
| --- | --- |
| 257200015 | Haemoglobin_estimation_NOS |
| 257185017 | Haemoglobin_low |
| 257186016 | Haemoglobin_borderline_low |

1. **Mean Corpuscular volume**

| 714931000006112 | Mean_corpuscular_volume_(MCV) |
| --- | --- |
| 257286011 | MCV_-_NOS |
| 257284014 | MCV_-_low |
| 132761000000116 | Mean_cell_volume |

**3. Platelet count**

| 102928018 | Platelet_count |
| --- | --- |
| 257443013 | Platelet_count_NOS |

1. **C- Reactive protein**

| 216602012 | Serum_C_reactive_protein_level |
| --- | --- |
| 223761000006118 | Plasma_C_reactive_protein |

1. **Erythrocyte sedimentation rate**

| 648521000006111 | Erythrocyte_sedimentation_rate |
| --- | --- |
| 648511000006115 | Erythrocyte_sediment_rate_NOS |

1. **Ferritin and Iron**

| 143821000006118 | Serum_ferritin |
| --- | --- |
| 1787464018 | Plasma_ferritin_level |
| 257523016 | Ferritin_level_low |
| 668851000006113 | Ferritin_-_serum |
| 144291000006119 | Serum_iron_level |
| 404437013 | Serum_iron_tests |
| 373199017 | Serum_iron_level |
| 257533012 | Serum_iron_tests_NOS |

1. **Vitamin B12**

| 145701000006116 | Serum_vitamin_B12 |
| --- | --- |
| 2159281019 | Plasma_vitamin_B12_level |
| 257555016 | Serum_vitamin_B12_NOS |
| 257553011 | Serum_vitamin_B12_low |
| 836101000006119 | Vitamin_B12_level |

1. **Calprotectin level**

| 2339451000000111 | Faecal_calprotectin_content |
| --- | --- |
| 731011000000115 | Calprotectin_level |
| 1897541000006119 | Faecal_calprotectin_test |

1. **Albumin (<35 g/dl)**

| 1484984011 | Plasma_albumin_level |
| --- | --- |
| 278130018 | Serum_albumin |

**Supplementary Table 2a: Demographic details of males with and without IBD in the development cohort.**

|  | **Symptomatic patients** | **%** | **IBD** | **%** | **UC** | **%** | **CD** | **%** |
| --- | --- | --- | --- | --- | --- | --- | --- | --- |
|  | 891,024 | 99.1 | 7,992 | 0.9 | 5,477 |  | 2,514 |  |
| **age (median)** | 49.0 | (IQR 34.8-64.7) | 43.2 | (IQR 29.7-57.8) | 44.7 | (IQR 31.4-58.9) | 39.1 | (IQR (27.2-55.2) |
| **BMI** | 26.4 | (IQR 23.7-29.7) | 26.0 | (IQR 23.2-29.4) | 26.1 | (IQR 23.5-29.5) | 25.9 | (IQR 22.6-29.0) |
| **BMI categories** |  |  |  |  |  |  |  |  |
| 18.5-25 | 260,431 | 29.2 | 2,381 | 29.8 | 1,635 | 29.9 | 746 | 29.7 |
| <18 | 18,226 | 2.1 | 195 | 2.4 | 108 | 2.0 | 87 | 3.5 |
| 25-30 | 285,045 | 32.0 | 2,355 | 29.5 | 1,662 | 30.4 | 693 | 27.6 |
| >30 | 176,240 | 19.8 | 1,380 | 17.3 | 982 | 17.9 | 398 | 15.8 |
| Missing | 151,082 | 17.0 | 1,681 | 21.0 | 1,090 | 19.9 | 590 | 23.5 |
| **Smoking status** |  |  |  |  |  |  |  |  |
| Never smoked | 278,928 | 31.3 | 2,325 | 29.1 | 1,575 | 28.8 | 749 | 29.8 |
| Ex-smoker | 300,115 | 33.7 | 2,785 | 34.9 | 2,078 | 37.9 | 707 | 28.1 |
| Current smoker | 290,726 | 32.6 | 2,614 | 32.7 | 1,649 | 30.1 | 965 | 38.4 |
| Missing | 21,255 | 2.4 | 268 | 3.4 | 175 | 3.2 | 93 | 3.7 |
| **Symptoms** |  |  |  |  |  |  |  |  |
| Nausea and vomiting | 67,908 | 7.62 | 191 | 2.39 | 103 | 1.9 | 88 | 3.5 |
| Abdominal pain | 355,387 | 39.9 | 1,835 | 23.0 | 932 | 17.0 | 903 | 35.9 |
| Diarrhoea | 198,815 | 22.3 | 2,403 | 30.1 | 1,694 | 30.9 | 709 | 28.2 |
| Mucus in stool | 504 | 0.1 | 20 | 0.3 | 17 | 0.3 | 3 | 0.1 |
| Anal symptoms | 50,390 | 5.7 | 297 | 3.7 | 133 | 2.4 | 164 | 6.5 |
| Weight loss | 51,860 | 5.8 | 158 | 2.0 | 80 | 1.5 | 78 | 3.1 |
| Bloating | 23,555 | 2.6 | 106 | 1.3 | 74 | 1.4 | 31 | 1.2 |
| Rectal bleeding | 120,112 | 13.5 | 2,543 | 31.8 | 2,133 | 38.94 | 410 | 16.31 |
| Change in bowel habit | 29,756 | 3.3 | 551 | 6.9 | 399 | 7.29 | 152 | 6.05 |
| Miscellaneous symptom | 6,350 | 0.7 | 44 | 0.6 | 32 | 0.58 | 12 | 0.48 |
| **Extraintestinal manifestations (EIM)** | | | | | | | | |
| Mouth ulcers | 17,425 | 2.0 | 208 | 2.6 | 148 | 2.7 | 60 | 2.4 |
| Primary sclerosing cholangitis | 35 | 0 | 6 | 0.1 | 4 | 0.1 | 2 | 0.1 |
| swollen joint | 42,975 | 4.8 | 304 | 3.8 | 206 | 3.8 | 97 | 3.9 |
| joint pain | 230,940 | 25.9 | 1,638 | 20.5 | 1,140 | 20.8 | 497 | 19.8 |
| Ophthalmic EIM | 8,773 | 1.0 | 99 | 1.2 | 64 | 1.17 | 35 | 1.39 |
| Dermatological EIM | 39,394 | 4.4 | 407 | 5.1 | 271 | 5.0 | 135 | 5.4 |
| **Comorbidity score** | | | | | | | | |
| 0 | 623,383 | 70.0 | 6,271 | 78.5 | 4,224 | 77.1 | 2,046 | 81.4 |
| 1 | 59,657 | 6.7 | 508 | 6.4 | 363 | 6.6 | 145 | 5.8 |
| 2 | 68,066 | 7.6 | 458 | 5.7 | 335 | 6.1 | 123 | 4.9 |
| >2 | 139,918 | 15.7 | 755 | 9.5 | 555 | 10.1 | 200 | 8.0 |
| **Co-existing conditions** | | | | | | | | |
| Anxiety | 102,935 | 11.6 | 828 | 10.4 | 553 | 10.1 | 275 | 10.9 |
| Depression | 130,434 | 14.6 | 1,005 | 12.6 | 660 | 12.1 | 345 | 13.7 |
| IBS | 27,361 | 3.1 | 314 | 3.9 | 190 | 3.5 | 124 | 4.9 |
| Haemorrhoids | 80,457 | 9.0 | 829 | 10.4 | 585 | 10.7 | 244 | 9.7 |
| Family history of IBD | 452 | 0.1 | 18 | 0.2 | 11 | 0.2 | 7 | 0.3 |
| Appendicectomy | 40195 | 4.5 | 195 | 2.4 | 99 | 1.8 | 96 | 3.8 |
| **Drugs** |  |  |  |  |  |  |  |  |
| loperamide | 26,778 | 3.0 | 328 | 4.1 | 228 | 4.2 | 100 | 4.0 |
| **Blood and stool tests** | | | | | | | | |
| Faecal calprotectin | 9,680 | 1.1 | 415 | 5.2 | 294 | 5.4 | 121 | 4.8 |
| Hb | 48,379 | 5.4 | 865 | 10.8 | 525 | 9.6 | 340 | 13.5 |
| MCV (<79fl) | 8,402 | 0.9 | 241 | 3.0 | 123 | 2.3 | 118 | 4.7 |
| ferritin (<20) | 4,213 | 0.5 | 153 | 1.9 | 111 | 2.0 | 42 | 1.7 |
| Vit B12 (<187) | 4,471 | 0.5 | 54 | 0.7 | 32 | 0.6 | 22 | 0.9 |
| Albumin (<35mg/dl) | 24,284 | 2.7 | 551 | 6.9 | 327 | 6.0 | 224 | 8.9 |
| Platelet (>400) | 11,757 | 1.3 | 519 | 6.5 | 279 | 5.1 | 240 | 9.6 |
| CRP(>5mg/dl) | 45,410 | 5.1 | 1,398 | 17.5 | 889 | 16.2 | 509 | 20.3 |
| ESR(>19mm/h) | 18,529 | 2.1 | 594 | 7.4 | 375 | 6.9 | 219 | 8.7 |

**IBD- Inflammatory bowel disease; UC- ulcerative colitis; CD- Crohn’s disease; IBS- irritable bowel syndrome; Hb- haemoglobin (<12.9g/dl in males and 11.9g/dl in females); MCV- mean corpuscular volume; Vit B12- Vitamin B12; CRP- C-reactive protein; ESR- erythrocyte sedimentation rate.**

**Supplementary Table 2b: Demographic details of females with and without IBD in the development cohort.**

|  | **Symptomatic patients** | **%** | **IBD** | **%** | **UC** | **%** | **CD** | **%** |
| --- | --- | --- | --- | --- | --- | --- | --- | --- |
|  | 1,148,401 | 99.4 | 7,113 | 0.6 | 4,537 |  | 2,574 |  |
| **age (median)** | 45.5 | (IQR 31.0-63.8) | 42.5 | (IQR 29.2-59.1) | 43.8 | (IQR 30.7-60.8) | 40.2 | (IQR 27.3-56.4) |
| **BMI** | 25.4 | (IQR 22.2-29.9) | 25.0 | (IQR 22-29.3) | 25 | (IQR 22-29.2) | 25.1 | (IQR 22-29.3) |
| **BMI categories** |  |  |  |  |  |  |  |  |
| 18.5-25 | 443,947 | 38.7 | 2,954 | 41.5 | 1,914 | 42.2 | 1,040 | 40.4 |
| <18 | 40,098 | 3.5 | 234 | 3.3 | 145 | 3.2 | 89 | 3.5 |
| 25-30 | 283,529 | 24.7 | 1,736 | 24.4 | 1,104 | 24.3 | 631 | 24.5 |
| >30 | 252,774 | 22.0 | 1,428 | 20.1 | 902 | 19.9 | 525 | 20.4 |
| Missing | 128,053 | 11.2 | 761 | 10.7 | 472 | 10.4 | 289 | 11.2 |
| **Smoking status** |  |  |  |  |  |  |  |  |
| Never smoked | 462,695 | 40.3 | 2,436 | 34.3 | 1,623 | 35.8 | 812 | 31.6 |
| Ex-smoker | 364,363 | 31.7 | 2,456 | 34.5 | 1,685 | 37.1 | 771 | 30.0 |
| Current smoker | 305,547 | 26.6 | 2,148 | 30.2 | 1,188 | 26.2 | 959 | 37.3 |
| Missing | 15,796 | 1.4 | 73 | 1.0 | 41 | 0.9 | 32 | 1.2 |
| **Symptoms** |  |  |  |  |  |  |  |  |
| Nausea and vomiting | 127,988 | 11.14 | 352 | 4.95 | 197 | 4.3 | 155 | 6.0 |
| Abdominal pain | 574,565 | 50.0 | 2,222 | 31.2 | 1,123 | 24.8 | 1,099 | 42.7 |
| Diarrhoea | 204,214 | 17.8 | 1,925 | 27.1 | 1,203 | 26.5 | 722 | 28.1 |
| Mucus in stool | 718 | 0.1 | 38 | 0.5 | 26 | 0.6 | 12 | 0.5 |
| Anal symptoms | 34,192 | 3.0 | 160 | 2.3 | 77 | 1.7 | 83 | 3.2 |
| Weight loss | 51,060 | 4.5 | 142 | 2.0 | 65 | 1.4 | 77 | 3.0 |
| Bloating | 52,885 | 4.6 | 189 | 2.7 | 117 | 2.6 | 72 | 2.8 |
| Rectal bleeding | 82,938 | 7.2 | 1,776 | 25.0 | 1511 | 33.3 | 264 | 10.3 |
| Change in bowel habit | 29,333 | 2.6 | 421 | 5.9 | 287 | 6.3 | 133 | 5.2 |
| Miscellaneous symptom | 6,693 | 0.6 | 40 | 0.6 | 26 | 0.6 | 14 | 0.5 |
| **Extraintestinal manifestations (EIM)** | | | | | | | | |
| Mouth ulcers | 28,026 | 2.4 | 210 | 3.0 | 122 | 2.7 | 88 | 3.4 |
| Primary sclerosing cholangitis | 37 | 0 | 4 | 0.1 | 4 | 0.1 | 0 | 0 |
| swollen joint | 63,706 | 5.6 | 315 | 4.4 | 201 | 4.4 | 113 | 4.4 |
| joint pain | 302,057 | 26.3 | 1,708 | 24.0 | 1,071 | 23.6 | 637 | 24.8 |
| Ophthalmic EIM | 11,268 | 1.0 | 113 | 1.6 | 64 | 1.4 | 47 | 1.83 |
| Dermatological EIM | 49,441 | 4.3 | 426 | 6.0 | 243 | 5.4 | 183 | 7.1 |
| **Comorbidity score** | | | | | | | | |
| 0 | 849,113 | 73.9 | 5,615 | 78.9 | 3,547 | 78.2 | 2,067 | 80.3 |
| 1 | 60,141 | 5.2 | 372 | 5.2 | 224 | 4.9 | 148 | 5.8 |
| 2 | 104,950 | 9.1 | 551 | 7.8 | 375 | 8.3 | 176 | 6.8 |
| >2 | 134,197 | 11.7 | 575 | 8.1 | 391 | 8.6 | 183 | 7.1 |
| **Co-existing conditions** | | | | | | | | |
| Anxiety | 192,352 | 16.8 | 1,063 | 14.9 | 650 | 14.3 | 413 | 16.1 |
| Depression | 256,113 | 22.3 | 1,549 | 21.8 | 955 | 21.1 | 594 | 23.1 |
| IBS | 75,257 | 6.6 | 628 | 8.8 | 354 | 7.8 | 274 | 10.6 |
| Haemorrhoids | 92,418 | 8.1 | 765 | 10.8 | 520 | 11.5 | 244 | 9.5 |
| Family history of IBD | 628 | 0.1 | 27 | 0.4 | 14 | 0.3 | 13 | 0.5 |
| Appendicectomy | 52637 | 4.6 | 238 | 3.4 | 116 | 2.6 | 122 | 4.7 |
| **Drugs** |  |  |  |  |  |  |  |  |
| loperamide | 29,225 | 2.5 | 325 | 4.6 | 187 | 4.1 | 138 | 5.4 |
| **Blood and stool tests** | | | | | | | | |
| Faecal calprotectin | 10,619 | 0.9 | 362 | 5.1 | 241 | 5.3 | 121 | 4.7 |
| Hb | 70,988 | 6.2 | 936 | 13.2 | 537 | 11.8 | 399 | 15.5 |
| MCV (<79fl) | 18,208 | 1.6 | 303 | 4.3 | 147 | 3.2 | 156 | 6.1 |
| ferritin (<20) | 26,051 | 2.3 | 388 | 5.5 | 246 | 5.4 | 142 | 5.5 |
| Vit B12 (<187) | 6,053 | 0.53 | 77 | 1.1 | 33 | 0.7 | 44 | 1.7 |
| Albumin (<35mg/dl) | 32,699 | 2.9 | 558 | 7.8 | 283 | 6.2 | 275 | 10.7 |
| Platelet (>400) | 25,353 | 2.2 | 662 | 9.3 | 321 | 7.1 | 341 | 13.3 |
| CRP(>5mg/dl) | 58,598 | 5.1 | 1,125 | 15.8 | 599 | 13.2 | 525 | 20.4 |
| ESR(>19mm/h) | 36,288 | 3.2 | 731 | 10.3 | 405 | 8.9 | 326 | 12.7 |

**IBD- Inflammatory bowel disease; UC- ulcerative colitis; CD- Crohn’s disease; IBs- irritable bowel syndrome; Hb- haemoglobin (<12.9g/dl in males and 11.9g/dl in females); MCV- mean corpuscular volume; Vit B12- Vitamin B12; CRP- C-reactive protein; ESR- erythrocyte sedimentation rate.**

**Supplementary Table 3: Adjusted hazard ratios for IBD in men and women in the development cohort complete case analysis in patients without faecal calprotectin.**

| **Predictors** | **IBD model** | |
| --- | --- | --- |
|  | **Men** | **Women** |
| **Smoking status** |  |  |
| Ex-smoker | 1.43 (1.34-1.53) | 1.37(1.29-1.46) |
| Current smoker | 1.18 (1.10-1.26) | 1.31(1.23-1.40) |
| **Body mass index** |  |  |
| Underweight | 1.06 (0.91-1.24) | 0.97 (0.85-1.12) |
| Overweight | 0.94 (0.89-1.00) | 0.90 (0.84-0.96) |
| Obese | 0.83 (0.77-0.89) | 0.75 (0.70-0.80) |
| **Comorbidity score** |  |  |
| 1 | 1.11 (1.00-1.23) | 1.01 (0.90-1.15) |
| 2 | 0.96 (0.86-1.07) | 0.92 (0.83-1.02) |
| >2 | 0.79 (0.72-0.87) | 0.84 (0.75-0.93) |
| **Symptoms** |  |  |
| Change in bowel habit | 3.66 (3.19-4.20) | 3.65 (3.14-4.24) |
| Abdominal pain | 0.81 (0.72-0.91) | 0.75 (0.66-0.85) |
| Nausea and vomiting | 0.44 (0.36-0.54) | 0.59 (0.50-0.69) |
| Diarrhoea | 2.04 (1.82-2.29) | 2.07 (1.83-2.35) |
| Mucus in stool | 5.19 (3.12-8.64) | 9.15 (6.46-12.96) |
| Weight loss | 0.47 (0.38-0.58) | 0.59 (0.47-0.73) |
| Bloating | 0.75 (0.58-0.96) | 0.73 (0.61-0.89) |
| Rectal bleeding | 3.79 (3.38-4.24) | 5.15 (4.54-5.83) |
| **Extraintestinal manifestation** |  |  |
| Ophthalmic EIM | 1.28 (1.02-1.61) | 1.64 (1.33-2.00) |
| Primary sclerosing cholangitis | 25.48 (10.57-61.40) | 14.72 (4.73-45.80) |
| Dermatological EIM | 1.19 (1.06-1.34) | 1.23 (1.10-1.37) |
| Joint pain | 0.82 (0.77-0.87) | 0.95 (0.89-1.01) |
| Joint swelling | NA | 0.88 (0.78-1.00) |
| **Co-existing conditions** |  |  |
| Anxiety | NA | 0.87 (0.81-0.93) |
| Depression | 0.90 (0.83-0.97) | NA |
| Irritable bowel syndrome | 1.22 (1.07-1.39) | 1.31 (1.20-1.44) |
| Haemorrhoids | 1.17 (1.07-1.27) | 1.28 (1.18-1.39) |
| Family history of IBD | 3.91 (2.39-6.39) | 5.49 (3.73-8.08) |
| Appendicectomy | 0.56 (0.47-0.66) | 0.70 (0.60-0.81) |
| Loperamide | 1.31 (1.15-1.49) | 1.46 (1.29-1.66) |
| **Blood tests** |  |  |
| Low haemoglobin | 1.56 (1.39-1.74) | 1.44 (1.30-1.58) |
| Low MCV | 1.46 (1.24-1.73) | 1.25 (1.08-1.45) |
| Low ferritin | 1.72 (1.40-2.12) | 1.48 (1.31-1.68) |
| Raised platelets | 2.48 (2.19-2.80) | 2.67 (2.42-2.96) |
| Raised CRP | 3.45 (3.18-3.75) | 2.63 (2.42-2.85) |
| Raised ESR | 1.75 (1.56-1.96) | 1.79 (1.62-1.98) |
| Low albumin | 1.98 (1.76-2.23) | 2.10 (1.88-2.34) |
| Low Vitamin B12 | NA | 1.41 (1.11-1.80) |

**Supplementary Table 4: IBD risk prediction model coefficients**

| **Predictors** | **Beta-coefficients** | |
| --- | --- | --- |
|  | **Males** | **Females** |
| Age 1 $\left( \left( \frac{age}{10} \right)^{-2}-mean \right)$* | 2.60 | 1.58 |
| Age 2 ${\left( \left( \frac{age}{10} \right)^{3}-mean \right)*}$ | -0.002 | -0.002 |
| **Smoking status (reference non-smoker)** | | |
| ex-smoker | 0.33 | 0.32 |
| current smoker | 0.09 | 0.27 |
| **Body mass index (reference normal)** | | |
| <18 | 0.03 | -0.02 |
| 25-30 | -0.05 | -0.11 |
| >30 | -0.18 | -0.30 |
| Missing | -0.01 | -0.12 |
| **Comorbidity score (reference 0 score)** | | |
| 1 | 0.07 | -0.001 |
| 2 | -0.09 | -0.11 |
| >2 | -0.27 | -0.22 |
| **Symptoms** |  |  |
| Change in bowel habit | 1.27 | 1.27 |
| Abdominal pain | -0.23 | -0.29 |
| Nausea and vomiting | -0.91 | -0.56 |
| Diarrhoea | 0.69 | 0.74 |
| Mucus in stool | 1.55 | 2.20 |
| Weight loss | -0.81 | -0.55 |
| Bloating | -0.31 | -0.32 |
| Rectal bleeding | 1.31 | 1.64 |
| Mouth ulcers | 0.25 | NA |
| Ophthalmic EIM | 0.26 | 0.47 |
| Primary sclerosing cholangitis | 1.98 | 2.50 |
| Joint swelling | NA | -0.14 |
| Joint pain | -0.20 | NA |
| Dermatological EIM | 0.12 | 0.24 |
| **Co-existing conditions** |  |  |
| Anxiety | NA | -0.15 |
| Depression | -0.12 | NA |
| Irritable bowel syndrome | 0.26 | 0.25 |
| Family history of IBD | 1.13 | 1.61 |
| Haemorrhoids | 0.17 | 0.23 |
| Appendicectomy | -0.61 | -0.34 |
| **Drugs** |  |  |
| Loperamide | 0.20 | 0.37 |
| **Blood tests** |  |  |
| Anaemia† | 0.49 | 0.37 |
| Raised platelets† | 0.96 | 1.02 |
| low MCV† | 0.32 | 0.23 |
| Low albumin† | 0.70 | 0.78 |
| Low vitamin B12† | NA | 0.31 |
| Low ferritin† | 0.67 | 0.39 |
| Raised CRP† | 1.34 | 0.97 |
| Raised ESR† | 0.60 | 0.64 |

***Fractional polynomials for age.** †**Haemoglobin-Hb<12.9g/dl in males and 11.9g/dl in females, mean corpuscular volume (MCV) <79fl, platelet >400 x10^9^/L, albumin level <35mg/dl, C-reactive protein (CRP) >5mg/dl, erythrocyte sedimentation rate (ESR) >19mm/h, ferritin <20 mg/L and Vitamin B12 <187 ng/L. IBD- inflammatory bowel disease, EIM- extraintestinal manifestation.**

**Supplementary Table 5. Adjusted hazard ratios for IBD, ulcerative colitis and Crohn’s disease in women in the development cohort with faecal calprotectin.**

| **Predictors** | **IBD model** | **Ulcerative colitis model** | **Crohn’s disease model** |
| --- | --- | --- | --- |
| **Smoking status** |  |  |  |
| Ex-smoker | 1.36 (1.28-1.44) | 1.37 (1.28-1.48) | 1.29 (1.16-1.43) |
| Current smoker | 1.30 (1.22-1.39) | 1.10(1.02-1.19) | 1.66 (1.50-1.83) |
| **Body mass index** |  |  |  |
| Underweight | 1.00 (0.87-1.15) | 1.06 (0.89-1.27) | 0.90 (0.72-1.13) |
| Overweight | 0.91 (0.85-0.97) | 0.87 (0.81-0.94) | 0.96 (0.87-1.07) |
| Obese | 0.75 (0.70-0.81) | 0.74 (0.68-0.81) | 0.77 (0.69-0.86) |
| Missing | 0.90 (0.82-0.97) | 0.89 (0.80-0.99) | 0.90 (0.78-1.03) |
| **Comorbidity score** |  |  |  |
| 1 | 1.01 (0.90-1.14) | 0.93 (0.80-1.08) | 1.17 (0.97-1.40) |
| 2 | 0.90 (0.82-0.99) | 0.91 (0.80-1.02) | 0.89 (0.75-1.06) |
| >2 | 0.81 (0.73-0.90) | 0.86 (0.76-0.98) | 0.75 (0.63-0.90) |
| **Symptoms** | | | |
| Change in bowel habit | 3.43 (2.97-3.97) | 3.57 (2.90-4.41) | 3.56 (2.85-4.45) |
| Abdominal pain | 0.76 (0.67-0.85) | 0.60 (0.50-0.73) | 1.22 (1.04-1.42) |
| Nausea and vomiting | 0.59 (0.50-0.68) | 0.54 (0.43-0.68) | 0.80 (0.64-0.99) |
| Diarrhoea | 2.01 (1.78-2.26) | 2.04 (1.68-2.46) | 2.32 (1.97-2.72) |
| Mucus in stool | 8.43 (6.03-11.79) | 8.87 (5.89-13.36) | 8.50 (4.67-15.48) |
| Weight loss | 0.60 (0.49-0.74) | 0.46 (0.34-0.63) | * |
| Bloating | 0.73 (0.61-0.88) | 0.70 (0.54-0.91) | * |
| Rectal bleeding | 4.99 (4.43-5.62) | 6.69 (5.54-8.07) | 2.31 (1.92-2.77) |
| Anal symptoms | * | 0.73 (0.54-0.99) | 1.87 (1.43-2.45) |
| **Extraintestinal manifestations** | | | |
| Mouth ulcers | 1.14 (0.99-1.32) | * | 1.31 (1.04-1.63) |
| Ophthalmic EIM | 1.56 (1.28-1.90) | 1.41 (1.09-1.83) | 1.71 (1.25-2.34) |
| Primary sclerosing cholangitis | 13.20 (4.25-41.01) | 22.10 (7.11-68.74) | * |
| Dermatological EIM | 1.28 (1.15-1.42) | 1.15 (1.00-1.33) | 1.51 (1.28-1.78) |
| Joint swelling | 0.87 (0.77-0.98) | 0.88 (0.75-1.02) | * |
| Joint pain | * | 0.88 (0.82-0.95) | * |
| **Co-existing conditions** | | | |
| Anxiety | 0.86 (0.80-0.92) | 0.82(0.75-0.89) | * |
| Irritable bowel syndrome | 1.30 (1.19-1.42) | 1.11 (0.99-1.25) | 1.62 (1.42-1.85) |
| Haemorrhoids | 1.23 (1.14-1.33) | 1.21(1.10-1.34) | 1.25 (1.09-1.44) |
| Family history of IBD | 4.48 (3.04-6.59) | 3.61(2.13-6.11) | 5.06 (2.86-8.96) |
| Appendicectomy | 0.71 (0.62-0.82) | 0.54 (0.45-0.66) | * |
| Loperamide | 1.49 (1.32-1.68) | 1.36 (1.16-1.59) | 1.67 (1.38-2.02) |
| **Blood tests** | | | |
| Low haemoglobin | 1.47 (1.34-1.61) | 1.48 (1.32-1.64) | 1.44 (1.25-1.67) |
| Low MCV | 1.26 (1.10-1.44) | * | 1.69 (1.39-2.06) |
| Low ferritin | 1.41 (1.26-1.59) | 1.53 (1.33-1.76) | 1.23 (1.01-1.50) |
| Raised platelets | 2.57 (2.33-2.82) | 1.99 (1.74-2.27) | 3.33 (2.89-3.83) |
| Raised CRP | 2.44 (2.25-2.64) | 2.17 (1.96-2.41) | 2.84 (2.51-3.22) |
| Raised ESR | 1.77 (1.61-1.94) | 1.69 (1.49-1.91) | 1.86 (1.61-2.14) |
| Low albumin | 2.14 (1.93-2.36) | 1.75 (1.53-2.01) | 2.68 (2.31-3.11) |
| Low Vitamin B12 | 1.42 (1.12-1.78) | * | 2.10 (1.54-2.85) |
| **Calprotectin level** | | | |
| <100 | 0.63 (0.46-0.87) | 0.58 (0.38-0.89) | 0.74 (0.44-1.23) |
| 100-199 | 4.63 (3.25-6.59) | 4.19 (2.63-6.67) | 5.82 (3.36-10.06) |
| 200-499 | 12.80 (10.12-16.18) | 12.32 (9.16-16.57) | 11.84 (8.06-17.39) |
| 500-999 | 15.96 (12.71-20.04) | 16.52 (12.45-21.93) | 11.47 (7.82- 16.82) |
| >1000 | 25.98 (20.96-32.20) | 31.34 (24.48-40.12) | 11.91 (7.68- 18.47) |
| FC tests with missing value | 3.70 (2.82-4.86) | 3.92 (2.82-5.46) | 3.48 (2.16-5.63) |
| ***Not included in final model as not associated.** | | | |

**Supplementary Table 6. Adjusted hazard ratios for IBD, ulcerative colitis and Crohn’s disease in men in the development cohort with faecal calprotectin.**

| **Predictors** | **IBD model** | **Ulcerative colitis model** | **Crohn’s disease model** |
| --- | --- | --- | --- |
| **Smoking status** |  |  |  |
| Ex-smoker | 1.38 (1.30-1.46) | 1.45 (1.35-1.55) | 1.28 (1.16-1.41) |
| Current smoker | 1.10 (1.03-1.16) | 1.01 (0.94-1.09) | 1.17 (1.05-1.31) |
| **Body mass index** |  |  |  |
| Underweight | 1.01 (0.87-1.18) | 0.97 (0.79-1.18) | 1.12 (0.89-1.42) |
| Overweight | 0.95 (0.89-1.00) | 0.91 (0.85-0.98) | 0.99 (0.88-1.10) |
| Obese | 0.84 (0.78-0.90) | 0.81 (0.75-0.88) | 0.85 (0.75-0.97) |
| Missing | 0.99 (0.93-1.06) | 0.98 (0.91-1.06) | 1.03 (0.91-1.15) |
| **Comorbidity score** | **Reference** |  |  |
| 1 | 1.09 (0-.99-1.21) | 1.14 (1.01-1.28) | 0.99 (0.82-1.20) |
| 2 | 0.91 (0.82-1.02) | 0.95 (0.84-1.08) | 0.85 (0.70-1.04) |
| >2 | 0.77(0.70-0.84) | 0.83 (0.74-0.92) | 0.68 (0.57-0.82) |
| **Symptoms** | | | |
| Change in bowel habit | 3.31 (2.92-3.75) | 4.03 (3.42-4.76) | 2.49 (2.08-2.98) |
| Abdominal pain | 0.79 (0.71-0.87) | 0.71 (0.61-0.83) | * |
| Nausea and vomiting | 0.41 (0.35-0.50) | 0.42 (0.32-0.53) | 0.46 (0.36-0.58) |
| Diarrhoea | 1.91 (1.72-2.11) | 2.45 (2.12-2.84) | 1.36 (1.22-1.51) |
| Mucus in stool | 4.75 (2.98-7.56) | 6.65 (3.99-11.09) | * |
| Weight loss | 0.46 (0.38-0.56) | 0.41 (0.32-0.54) | 0.59 (0.46-0.75) |
| Bloating | 0.72 (0.57-0.90) | * | 0.60 (0.41-0.87) |
| Rectal bleeding | 3.60 (3.26-3.99) | 5.46 (4.72-6.30) | 1.40 (1.24-1.58) |
| Anal symptoms | * | 0.76 (0.60-0.95) | 1.48 (1.24-1.76) |
| **Extraintestinal manifestation** | | | |
| Mouth ulcers | 1.27 (1.10-1.46) | 1.38 (1.16-1.63) |  |
| Ophthalmic EIM | 1.30 (1.05-1.60) | * | 1.43 (0.99-2.06) |
| Primary sclerosing cholangitis | 8.04 (3.58-18.07) | 7.72 (2.86-20.81) | 12.35 (3.05-50.03) |
| Dermatological EIM | 1.12 (1.01-1.24) | * | 1.29 (1.07-1.54) |
| Joint pain | 0.82 (0.77-0.87) | 0.81 (0.76-0.87) | 0.85 (0.74-0.98) |
| **Co-existing conditions** | | | |
| Depression | 0.89 (0.83-0.96) | 0.83 (0.77-0.91) | * |
| Irritable bowel syndrome | 1.29 (1.15-1.46) | * | 1.63 (1.34-1.98) |
| Haemorrhoids | 1.19 (1.10-1.28) | 1.12 (1.03-1.23) | 1.33 (1.16-1.53) |
| Family history of IBD | 3.13 (1.95-5.04) | 2.69 (1.44-5.00) | 4.34 (2.07-9.13) |
| Appendicectomy | 0.55 (0.47-0.64) | 0.39 (0.31-0.48) |  |
| Loperamide | 1.25 (1.11-1.41) | 1.24 (1.07-1.43) | 1.30 (1.05-1.05-1.62) |
| **Blood tests** |  |  |  |
| Low haemoglobin | 1.64 (1.48-1.81) | 1.40 (1.24-1.58) | 2.14 (1.80-2.54) |
| Low MCV | 1.21 (1.04-1.41) | * | 1.74 (1.40-2.17) |
| Low ferritin | 2.04 (1.71-2.44) | 2.42 (1.98-2.96) | 1.41 (1.01-1.97) |
| Raised platelets | 2.47 (2.22-2.75) | 1.97 (1.71-2.27) | 3.07 (2.58-3.65) |
| Raised CRP | 3.44 (3.20-3.70) | 3.33 (3.04-3.63) | 3.58 (3.16-4.06) |
| Raised ESR | 1.84 (1.66-2.04) | 1.91 (1.68-2.17) | 1.58 (1.33-1.88) |
| Low albumin | 1.98 (1.78-2.21) | 1.80 (1.57-2.06) | 2.19 (1.83-2.62) |
| **Calprotectin level** |  |  |  |
| <100 | 0.89 (0.69-1.14) | 0.93 (0.69-1.25) | 0.82 (0.50-1.34) |
| 100-199 | 4.03 (2.78-5.85) | 3.06 (1.84-5.08) | 6.65 (3.85-11.49) |
| 200-499 | 10.67 (8.47-13.44) | 8.24 (6.05-11.23) | 14.65 (10.35-20.75) |
| 500-999 | 17.53 (14.30-21.49) | 17.05 (13.44-21.61) | 10.84 (7.26-16.18) |
| >1000 | 19.85 (16.01-24.62) | 24.59 (19.37-31.21) | 5.33 (3.28-8.66) |
| FC tests with missing value | 3.34 (2.60-4.28) | 3.60 (2.70-4.81) | 2.44 (1.49-4.00) |
| ***Not included in final model as not associated.** | | | |

**Supplementary Table 7. Mean (95% confidence interval) performance measures of IBD, ulcerative colitis and Crohn’s disease in women and men with faecal calprotectin.**

| **Development Cohort** | | | | | | |
| --- | --- | --- | --- | --- | --- | --- |
|  | **Men** |  |  | **Women** |  |  |
|  | **C-statistic^*^** | **D-statistic^*^** | **R2D** | **C-statistic^*^** | **D-statistic^*^** | **R2D** |
| **Inflammatory bowel disease** |  |  |  |  |  |  |
| **1 year** | 0.81 (0.80-0.81) | 2.14 (2.10-2.19) | 0.52 | 0.82 (0.81-0.83) | 2.27 (2.22-2.32) | 0.55 |
| **2 year** | 0.79 (0.78-0.79) | 1.97 (1.93-2.02) | 0.48 | 0.80 (0.79-0.80) | 2.06 (2.01-2.11) | 0.50 |
| **3 year** | 0.78 (0.77-0.78) | 1.89 (1.85-1.93) | 0.46 | 0.78 (0.78-0.79) | 1.93 (1.89-1.98) | 0.47 |
| **5 year** | 0.77 (0.76-0.78) | 1.79 (1.75-1.83) | 0.43 | 0.77 (0.77-0.78) | 1.80 (1.76-1.84) | 0.44 |
| **Ulcerative colitis** |  |  |  |  |  |  |
| **1 year** | 0.83 (0.83-0.84) | 2.29 (2.23-2.34) | 0.56 | 0.85 (0.84-0.85) | 2.44 (2.37-2.50) | 0.59 |
| **2 year** | 0.82 (0.81-0.82) | 2.12 (2.07-2.17) | 0.52 | 0.82 (0.81-0.83) | 2.24 (2.18-2.29) | 0.54 |
| **3 year** | 0.81 (0.80-0.81) | 2.04 (1.99-2.08) | 0.50 | 0.81 (0.80-0.82) | 2.10 (2.04-2.15) | 0.51 |
| **5 year** | 0.80(0.79-0.80) | 1.93 (1.88-1.97) | 0.47 | 0.79 (0.79-0.80) | 1.93 (1.88-1.98) | 0.47 |
| **Crohn’s disease** |  |  |  |  |  |  |
| **1 year** | 0.77 (0.76-0.78) | 2.05 (1.97-2.13) | 0.50 | 0.79 (0.77-0.80) | 2.16 (2.07-2.25) | 0.53 |
| **2 year** | 0.75 (0.74-0.77) | 1.88 (1.80-1.95) | 0.46 | 0.77 (0.76-0.78) | 1.96 (1.88-2.03) | 0.48 |
| **3 year** | 0.75 (0.74-0.76) | 1.81 (1.74-1.88) | 0.44 | 0.76 (0.75-0.77) | 1.86 (1.79-1.93) | 0.45 |
| **5 year** | 0.74 (0.73-0.75) | 1.72 (1.65-1.78) | 0.41 | 0.75 (0.74-0.77) | 1.77 (1.70-1.84) | 0.43 |
| **Validation Cohort** | | | | | | |
| **Inflammatory bowel disease** |  |  |  |  |  |  |
| **1 year** | 0.81 (0.80-0.83) | 2.19 (2.11-2.28) | 0.53 | 0.83 (0.81-0.84) | 2.32 (2.22-2.42) | 0.56 |
| **2 year** | 0.80 (0.79-0.81) | 2.06 (1.98-2.14) | 0.50 | 0.81 (0.79-0.82) | 2.12 (2.04-2.21) | 0.52 |
| **3 year** | 0.79 (0.78-0.80) | 1.97 (1.89-2.04) | 0.48 | 0.79 (0.78-0.81) | 1.99 (1.91-2.07) | 0.49 |
| **5 year** | 0.78 (0.77-0.79) | 1.85 (1.78-1.93) | 0.45 | 0.78 (0.77-0.79) | 1.84 (1.76-1.92) | 0.45 |
| **Ulcerative colitis** |  |  |  |  |  |  |
| **1 year** | 0.84 (0.83-0.86) | 2.29 (2.19-2.40) | 0.56 | 0.86 (0.85-0.88) | 2.48 (2.35-2.61) | 0.60 |
| **2 year** | 0.82 (0.81-0.84) | 2.15 (2.05-2.24) | 0.52 | 0.84 (0.82-0.85) | 2.28 (2.16-2.39) | 0.55 |
| **3 year** | 0.82 (0.81-0.83) | 2.07 (1.98-2.16) | 0.51 | 0.82 (0.81-0.84) | 2.14 (2.04-2.25) | 0.52 |
| **5 year** | 0.81 (0.80-0.82) | 1.96 (1.87-2.05) | 0.48 | 0.81 (0.79-0.82) | 1.97 (1.87-2.07) | 0.48 |
| **Crohn’s disease** |  |  |  |  |  |  |
| **1 year** | 0.80 (0.78-0.83) | 2.32 (2.17-2.47) | 0.56 | 0.79 (0.77-0.82) | 2.28 (2.12-2.43) | 0.55 |
| **2 year** | 0.79 (0.77-0.81) | 2.20 (2.06-2.33) | 0.54 | 0.78 (0.76-0.80) | 2.09 (1.95-2.23) | 0.51 |
| **3 year** | 0.78 (0.76-0.80) | 2.07 (1.94-2.20) | 0.51 | 0.77 (0.75-0.79) | 1.96 (1.83-2.09) | 0.48 |
| **5 year** | 0.77 (0.75-0.79) | 1.95 (1.83-2.07) | 0.48 | 0.76 (0.74-0.78) | 1.78 (1.66-1.89) | 0.43 |

***A measure of discrimination: higher values indicate better discrimination**

**Supplementary Table 8:** **Adjusted hazard ratios for IBD in men and women in the development cohort complete case analysis with faecal calprotectin.**

| **Predictors** | **Men** | **Women** |
| --- | --- | --- |
| **Smoking status** |  |  |
| Ex-smoker | 1.42 (1.33-1.52) | 1.36 (1.27-1.45) |
| Current smoker | 1.19 (1.12-1.28) | 1.30 (1.22-1.39) |
| **Body mass index** |  |  |
| Underweight | 1.03 (0.89-1.21) | 1.00 (0.87-1.15) |
| Overweight | 0.94 (0.88-1.00) | 0.91 (0.85-0.97) |
| Obese | 0.83 (0.77-0.89) | 0.76 (0.71-0.81) |
| **Comorbidity score** |  |  |
| 1 | 1.12 (1.01-1.25) | 1.02 (0.90-1.15) |
| 2 | 0.96 (0.86-1.07) | 0.92 (0.83-1.02) |
| >2 | 0.79 (0.72-0.87) | 0.84 (0.75-0.93) |
| **Symptoms** |  |  |
| Change in bowel habit | 3.47 (3.02-3.98) | 3.49 (3.00-4.06) |
| Abdominal pain | 0.81 (0.72-0.91) | 0.76 (0.67-0.86) |
| Nausea and vomiting | 0.45 (0.37-0.55) | 0.60 (0.51-0.71) |
| Diarrhoea | 1.96 (1.74-2.19) | 1.97 (1.74-2.24) |
| Mucus in stool | 5.31 (3.19-8.84) | 8.49 (5.99-12.05) |
| Weight loss | 0.49 (0.40-0.60) | 0.61 (0.49-0.76) |
| Bloating | 0.73 (0.57-0.94) | 0.73 (0.60-0.89) |
| Rectal bleeding | 3.71 (3.31-4.16) | 4.99 (4.40-5.66) |
| **Extraintestinal manifestation** |  |  |
| Mouth ulcers | 1.18 (1.00-1.40) | * |
| Ophthalmic EIM | 1.27 (1.01-1.60) | 1.60 (1.30-1.96) |
| Primary sclerosing cholangitis | 26.65 (11.06-64.23) | 15.98 (5.14-49.70) |
| Dermatological EIM | 1.18(1.05-1.33) | 1.25 (1.12-1.40) |
| Joint pain | 0.82 (0.77-0.87) | * |
| Joint swelling | * | 0.87 (0.77-0.99) |
| **Co-existing conditions** |  |  |
| Anxiety | * | 0.86 (0.80-0.92) |
| Depression | 0.90 (0.83-0.97) | * |
| Irritable bowel syndrome | 1.21 (1.06-1.39) | 1.32 (1.20-1.44) |
| Haemorrhoids | 1.17 (1.08-1.27) | 1.25 (1.15-1.36) |
| Family history of IBD | 3.91 (2.39-6.39) | 4.78 (3.25-7.04) |
| Appendicectomy | 0.56 (0.48-0.66) | 0.70 (0.60-0.81) |
| Loperamide | 1.32 (1.16-1.50) | 1.49 (1.31-1.69) |
| **Blood tests** |  |  |
| Low haemoglobin | 1.55 (1.39-1.73) | 1.46 (1.33-1.61) |
| Low MCV | 1.32 (1.12-1.56) | 1.25 (1.08-1.45) |
| Low ferritin | 1.78 (1.44-2.19) | 1.41 (1.25-1.60) |
| Raised platelets | 2.39 (2.11-2.70) | 2.48 (2.24-2.75) |
| Raised CRP | 3.10 (2.86-3.37) | 2.42 (2.22-2.63) |
| Raised ESR | 1.74 (1.55-1.95) | 1.68 (1.53-1.86) |
| Low albumin | 1.98 (1.76-2.23) | 2.06 (1.85-2.29) |
| Low Vitamin B12 | * | 1.46 (1.14-1.86) |
| **Calprotectin level** |  |  |
| <100 | 0.92 (0.69-1.22) | 0.60 (0.43-0.86) |
| 100-199 | 3.53(2.25-5.54) | 4.52 (3.10-6.61) |
| 200-499 | 12.03 (9.25-15.65) | 12.63 (9.89-16.14) |
| 500-999 | 20.00 (15.94-25.08) | 17.45 (13.71-22.22) |
| >1000 | 24.64 (19.29-31.48) | 28.68 (22.91-35.90) |
| FC tests with missing value | 3.12 (2.33-4.17) | 3.97 (3.01-5.24) |
