# Supplementary material for: Development and validation of a risk prediction tool for the diagnosis of inflammatory bowel disease in patients presenting in primary care with abdominal symptoms
Source: J Crohns Colitis. 2025 Mar 18;19(4):jjaf044. doi: 10.1093/ecco-jcc/jjaf044 (PMC12010163; doi:10.1093/ecco-jcc/jjaf044)
Supplement: Supplementary file 4 [file jjaf044_suppl_supplementary_figure_s1.docx]

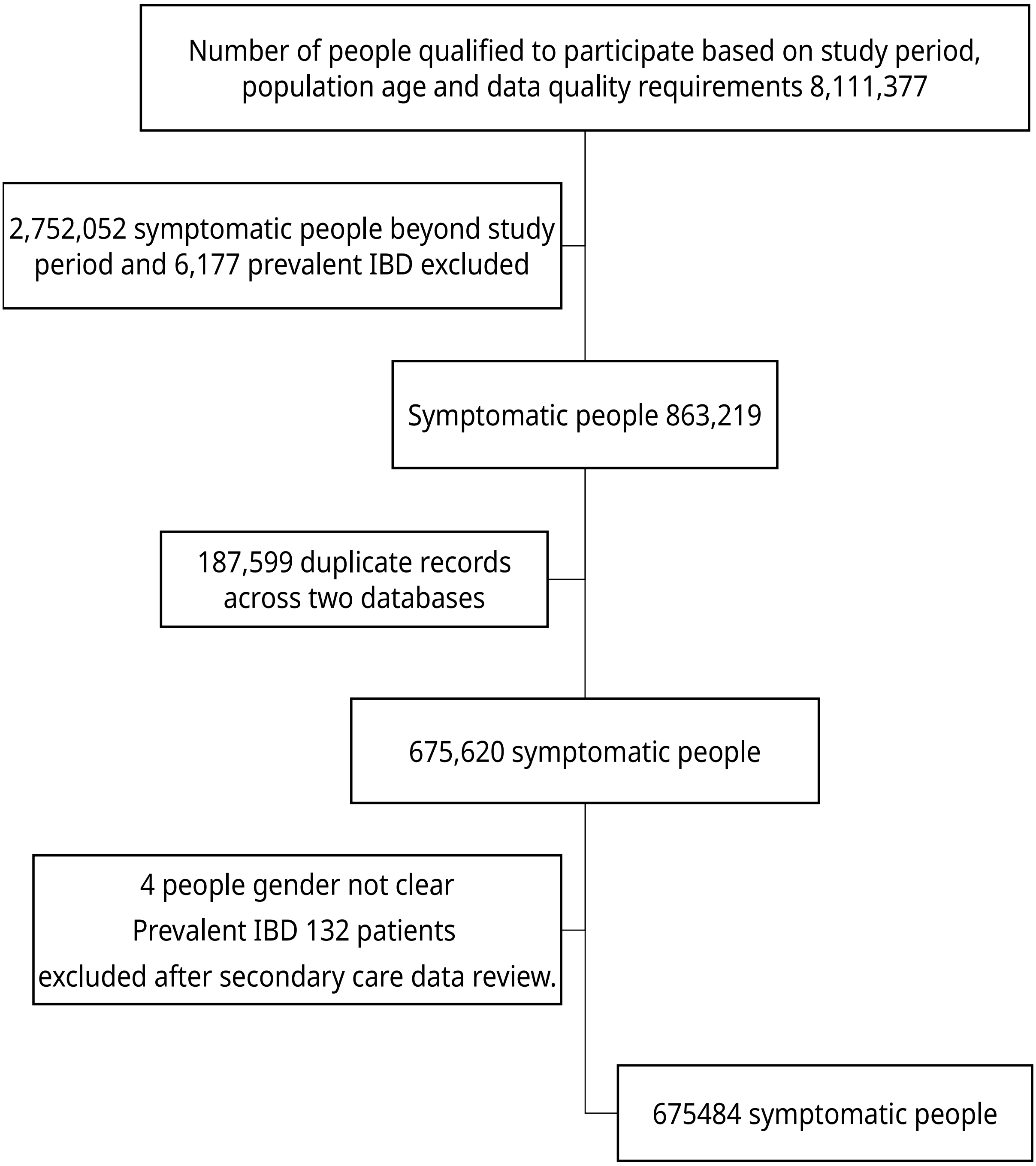
Number of people qualified to participate based on study period, population age and data quality requirements 8,111,377
2,752,052 symptomatic people beyond study period and 6,177 prevalent IBD excluded
Symptomatic people 863,219
187,599 duplicate records across two databases
675,620 symptomatic people
4 people gender not clear
Prevalent IBD 132 patients
excluded after secondary care data review.
675484 symptomatic people
